# Essential New Complexity-Based Themes for Patient-Centered Diagnosis and Treatment of Dementia and Predementia in Older People: Multimorbidity and Multilevel Phenomenology

**DOI:** 10.3390/jcm13144202

**Published:** 2024-07-18

**Authors:** Eli Wertman

**Affiliations:** 1 Department of Neurology, Hadassah University Hospital, The Hebrew University, Jerusalem 9190500, Israel; eli@merhav-clinic.co.il; 2 Section of Neuropsychology, Department of Psychology, The Hebrew University, Jerusalem 9190500, Israel; 3 Or’ad: Organization for Cognitive and Behavioral Changes in the Elderly, Jerusalem 9458118, Israel; 4 Merhav Neuropsychogeriatric Clinics, Nehalim 4995000, Israel

**Keywords:** dementia, predementia, elderly, complex system, heterogeneity, multimorbidity, remediability, functional neuroanatomy

## Abstract

Dementia is a highly prevalent condition with devastating clinical and socioeconomic sequela. It is expected to triple in prevalence by 2050. No treatment is currently known to be effective. Symptomatic late-onset dementia and predementia (SLODP) affects 95% of patients with the syndrome. In contrast to trials of pharmacological prevention, no treatment is suggested to remediate or cure these symptomatic patients. SLODP but not young onset dementia is intensely associated with multimorbidity (MUM), including brain-perturbating conditions (BPCs). Recent studies showed that MUM/BPCs have a major role in the pathogenesis of SLODP. Fortunately, most MUM/BPCs are medically treatable, and thus, their treatment may modify and improve SLODP, relieving suffering and reducing its clinical and socioeconomic threats. Regrettably, the complex system features of SLODP impede the diagnosis and treatment of the potentially remediable conditions (PRCs) associated with them, mainly due to failure of pattern recognition and a flawed diagnostic workup. We suggest incorporating two SLODP-specific conceptual themes into the diagnostic workup: MUM/BPC and multilevel phenomenological themes. By doing so, we were able to improve the diagnostic accuracy of SLODP components and optimize detecting and favorably treating PRCs. These revolutionary concepts and their implications for remediability and other parameters are discussed in the paper.

## 1. Introduction

Dementia and predementia are highly prevalent [1,2,3,4], with high personal, caregiver, societal, and economic costs [5,6]. They are predicted to increase two- to four-fold in the coming two to three decades [7]. Currently, no treatment is available for all types of dementia [8,9,10,11,12,13,14]; this has resulted in almost complete negligence of a regenerative treatment of symptomatic dementia [9]. The maximum prevention potential of less than 40% [15,16] suggests that the majority of patients with dementia will receive medical attention only when they are clinically symptomatic. Without reviving classical regenerative medical treatments [15,17,18], patients and caregivers are left to suffer for years without any hope for medical help.

There is an urgent need for a new disease-modifying strategy for symptomatic patients that should be patient-centered, clinically relevant, and unequivocally medically, cognitively, behaviorally, functionally, and socioeconomically significant.

This strategy should be based on identifying Multimorbidity (MUM), Brain Perturbative Conditions (BPCs), and Potential Remediable Conditions (PRCs) in patients with Symptomatic Late-Onset Dementia and Predementia (SLODP). SLODP is a unique nosological entity [15,19] with a high frequency of MUM that affects the brain [20]. Most MUMs are medically treatable [21], with treatment frequently reported to remediate dementia [22]. Thus, the reported failure to affect SLODP by treating systemic disorders is hard to accept [9], as is the lack of a compelling effect of treating on socioeconomic burden.

In this article, we present a review and the author’s personal view based on the experience of frequent diagnosis of coexisting treatable and PRCs of MUM and BPCs per patient in a large (N = 7000) population of subjects with SLODP. Many of them reported remediation when treated.

We discuss the major role of MUM and BPCs in the pathophysiology of SLODP and their significant potential target for its treatment. We also consider the reasons for the current low rate of diagnosis of remediable MUM/BPC/PRC conditions.

For optimizing the identification of MUM/BPC/PRC conditions in SLODP, there is a need to overcome its clinical heterogeneity and complexity. Based on the complex system (CxS) features of SLODP, we conceptualized MUM/BPCs and multilevel phenomenological themes in the diagnostic workup. Recent advances in behavioral/cognitive neurology, neuropsychology, neuropsychiatry, geriatrics, and information sciences helped us achieve this goal [23]. Two clinical vignettes are provided to demonstrate how these themes can be applied. Wider clinical and theoretical implications for the fields of SLODP are suggested. 

## 2. Background

This article represents the personal view of the author based on 30 years of clinical experience with 7000 patients with SLODP, whose data formed the basis for the insights and principles presented here. It is based on work carried out in a specific neuropsychogeriatric clinic. The information was gathered through a thorough diagnostic process (careful examination of each patient’s medical, cognitive, behavioral, and functional state), a review of previous discharge letters and consultation documents; a systems review and review of the patient’s cognitive and behavioral status; and an evaluation of changes in activities of daily living and independence, as well as geriatric functioning (e.g., vision, hearing, and sleep habits), medications, psychosocial state, and parallel progressive or transient systemic events (e.g., vertigo, syncope, falls, infections, and pharmacological needs). Collateral information was always used. Each patient was evaluated by a team consisting of a behavioral neurologist, geriatrician, neuropsychologist, geriatric or general psychiatrist (if needed), and nurse. Presentation and trajectory were determined. A diagnostic consensus was arrived at for the specific type of SLODP and PRCs.

A detailed document was prepared for each patient. It was always discussed with caregivers and, when necessary, with the patient’s primary care physician. Case management and follow-up were carried out as needed based on this plan.

The concepts described here have been accepted and discussed in many academic and clinical settings in Israel and abroad.

## 3. SLODP and MUM as Potential Bases for the Treatment of Dementia

Aging and SLODP have a high prevalence of MUM. Since SLODP is a nosological entity distinct from young-onset dementia, the epidemiological rates of MUM should be recognized. The pathological and pathophysiological effects of MUM on SLODP are discussed in order to realize their potential role in the treatment of the syndrome. 

### 3.1. SLODP as a Nosological Entity 

SLODP seems to be nosologically distinct from young-onset dementia (YOD), as suggested by its occurrence in approximately 95% of patients with dementia [2,3]; a strong correlation between incidence and aging, indicated by a 10% to 20% prevalence among individuals greater than 65 years of age [2,3] compared with 0.04% to 0.07% in a younger population [24,25,26]; a 40-fold increase in prevalence at approximately age 65 years [27,28] and a 250-fold increase in incidence from 85 to 100 years [29,30]; its increasing prevalence with advancing age compared with stability before age 65 years [27,28]; SLODP aging-related and unique individual neuropathological changes [31,32,33,34,35]; the decrease with age of its defining criteria [36,37]; its association with (a) genes that produce key proteins involved in the metabolism of cholesterol and lipids, (b) endocytocic and inflammatory and immune responses, and (c) favoring of APOE ꜫ4 alleles in contrast with the YOD-associated production and degradation of Aβ genes [38,39,40,41]; differences in the presentation of SLODP versus YOD (amnesia vs executive language, visuospatial, and motor function) [41]; greater heterogeneity [41,42,43]; static and functional neuroimaging features [44,45,46]; and the much greater frequency of MUM [47,48].

These differences suggest that SLODP is nosologically independent of YOD.

### 3.2. MUM: Potential Basis for Remediable Treatment of Patients with SLODP

MUM is the coexistence of chronic and acute conditions with the effects of medications, lifestyle behaviors, disability, and socioeconomic stressors [49,50]. MUM includes subclinical conditions that affect the patient’s medical state [51]. MUM is distinct from comorbidity, however, which emphasizes an index disease [52,53,54].

The relevance of MUM to the pathophysiology of SLODP is indicated by its high prevalence, its multifactorial contribution to cognitive loss [55,56], the fact that common neurodegenerative neuropathologies cannot be used to explain clinical/cognitive variation among older people [57,58], and the multiple heterogeneous brain lesions caused by MUM [59,60].

We suggest that MUM has a major role in the pathophysiology of SLODP and, therefore, may be a target for its treatment.

### 3.3. The Epidemiological and Causal Association of MUM and SLODP

The rate of MUM is higher in patients with SLODP compared with age-matched controls [61,62]. Additionally, the age-related increase in MUM runs parallel to the increased rate [61,62,63,64,65,66], severity, and progression of SLODP [40,63,67]; its rate of conversion to mild cognitive impairment (MCI) is parallel to that for SLODP [68]; and the number of MUMs in patients with Down syndrome is increased in those who have dementia [69].

The MUM effect seems to be causal rather than correlational, as indicated by the higher risk for dementia at a younger age of onset with MUM [70], the frequent association of most MUM conditions with specific cognitive-behavioral phenomena (e.g., dysexecutive memory and word-generation deficits) [71,72,73,74,75,76,77], the reported positive clinical effect of treatment of several MUM conditions [77,78,79,80], and the parallel clinical heterogeneity of SLODP and MUM effects.

The effects of MUM in SLODP suggest the existence of pathophysiological determinants in addition to those associated with each MUM component.

### 3.4. MUM—A Constitutional Feature of Aging 

MUM is a constitutional feature of aging. Its rate among patients older than 65 years is high (two conditions are seen in 60% to 98% of these patients [81,82] and 6 to 9 in 40% of patients [83]) and increases markedly with age [83,84]. It is universal [84] and increases constantly [85,86]. Hypertension, dyslipidemias, ischemic heart disease, and diabetes are the most prevalent MUM conditions [87].

The rate seems higher than reported due to contributing conditions, including acute medical states, underdiagnosed conditions (e.g., hypotension [88] or delirium [89]), masked coexisting conditions (e.g., as mild anemia or heart failure with a preserved ejection fraction [90,91]), metabolic conditions or sleep disorders in the presence of SLODP [92,93], underreported complications of major conditions (e.g., hypoglycemia in type 2 diabetes mellitus), subclinical conditions (e.g., subclinical hypothyroidism), low-severity conditions (e.g., moderate weight loss), diagnosed but partially treated MUM conditions (e.g., hypertension [94]), and some apparently unrelated conditions (e.g., periodontitis [95]).

Frequent clinical contingencies are associated with a further increase in MUM clusters in older people, including SLODP [96], frequent core medical disorders (e.g., heart failure [HF] [97], hypertension [98], diabetes mellitus [99], ischemic heart disease, stroke, AF [100], and other diseases [101,102,103]), as well as geriatric syndromes [104,105] (e.g., frailty [106] and other conditions [107]), prevalent syndromes of older people (e.g., gait disturbance [108], pain [109], and hearing loss [110]), neuropsychiatric disorders [111], and lifestyle behaviors (e.g., smoking [112], insomnia [113,114], decreased physical activity [115], as well as loneliness [116]), polypharmacy [117], poor quality of life [118], and loss of independence [119].

MUM is therefore not only correlated and causally related to SLODP, but it is also a constitutional feature of aging [120,121] and, like gravity, activates the downhill progression of older people to SLODP.

### 3.5. Specific Effects of MUM on the Development of SLODP 

#### 3.5.1. Direct Effects of MUM on Multilevel Neuropathology in SLODP

*Neuropathological effects of MUM.* The rate of mixed neuropathology is high in autopsy studies of AD (e.g., 60–78% in vascular-AD patients [122]) and other neurodegenerative disorders in patients with SLODP [122,123,124]. The number of neuropathological changes increases in the presence of coexisting conditions such as vitamin B12 deficiency, hypoglycemic events in type 2 diabetes, and other MUM conditions [20,125,126] that increase relentlessly with SLODP.

*Neuroimaging of global MUM effects*. Neuroimaging reveals structural changes (reduced total brain volume and grey matter, increased white matter intensity, and accelerated aging pathology) [127] and functional changes (widespread changes in specific cortical and subcortical areas [128]) and changes in the default mode network [129] associated with MUM in patients with SLODP.

*Coexisting MUM mechanisms in SLODP.* MUM is responsible for the coexistence of diverse lesions in brain and vascular tissue (from large to capillary vessels), which results in diverse pathophysiological mechanisms and distinct presentations (e.g., vascular dementia associated with subcortical ischemic infarcts, a cortical watershed, focal ischemia and MCI, cortical and subcortical dementias, and focal cognitive deficits) [130,131,132]. Metabolic disorders, medications, and other MUM neuropathologies add to these effects through large- or small-scale systems [133]. The multiple MUM conditions affect the brain both additively and synergistically [70,134].

#### 3.5.2. MUM-Associated Molecular Effects in SLODP 

MUM triggers many molecular biological mechanisms (e.g., oxidative stress, cellular senescence, inflammation, and mitochondrial dysfunction [135,136]) that contribute to hypoxia [137,138], sepsis [139], hyperglycemia [140], hypoglycemia [141], and hypothyroidism [142,143,144,145]. MUM molecular effects also have specificity for distinct subcellular changes, such as for COPD and CHF [146,147,148]. Specificity is reported in different studies [149]. The specificity of the effects of each MUM that may be associated with the molecular specificity of that MUM was also reported for tau/Aβ processes (e.g., different pathways in obesity [150] and oligemic hypoperfusion [151]). MUM has an integrated system effect on the neuropathophysiology of SLODP (AD [152], vascular dementia [153], and other proteinopathies [154,155]).

MUM enhances the pathophysiology of SLODP which is also associated with general processes that affect SLODP, such as aging and geriatric syndromes that lower resilience. The effects of MUM on aging include the acceleration of aging, especially in the brain [156,157], enhancement of the biological mechanisms of aging, and overlapping of the molecular mechanisms of aging, MUM, and cognitive decline [80,145]. Geriatric syndromes (such as frailty) are risk factors for SLODP, and neuropsychiatric conditions [158,159] are associated with MUM [103,160]. Molecular mechanisms that link geriatric syndromes (such as frailty) with cognitive decline [161] (e.g., oxidative stress and epigenetic changes [162]) are also activated by MUM [80].

Resilience—the ability to adapt successfully to adversity [163]—is elementary in the maintenance and recovery of function after biomedical or pathological challenges [164]. It is patient-dependent and individually activated [165,166]. Resilience is established through complex neurobiological processes that involve limbic structures and systems, the blood–brain barrier (BBB), and other structures and mechanisms [163,164,165,166,167]. MUM seems to affect these processes and cause a loss of resilience [168,169].

#### 3.5.3. Direct Phenomenological Effects of MUM in SLODP

MUM might modulate cognitive and behavioral deficits in SLODP directly, e.g., through fatigue (in systemic disease or a sleep disorder) [125], a confusional state (e.g., infection or low blood pressure), slowly developing cognitive changes (e.g., in hypoxemia, orthostatic hypotension, or hypoglycemic events), depression-like behavior, hearing loss, and more [125].

### 3.6. Summary—A Potential Role for MUM in the Pathophysiology and Treatment of SLODP

Most etiological diagnoses of dementia are degenerative and irreversible. No effective treatment [10] or prevention protocols [148,170] have yet been found. The high rate of MUM suggests its causal role in SLODP and high potential for treatment and remediability. We believe that this role accounts for the 10 recently published critical questions and challenges of a valid theory for SLODP and Alzheimer’s disease in older people [1]. We discuss the reasons for the very low rate of detecting PRCs. We assume that MUM/BPCs/PRCs are the rule rather than the exception in older people, and they should always be looked for in patients with SLODP. These will be a basis for introducing new concepts to the diagnostic workup.

## 4. Low Rate of Diagnosed Remediable Conditions in SLODP

It is hard to accept the low rates of treated PRCs (7–23%) and remediability of SLODP (0.6–4.0%) [21]. Also, in spite of a high rate of MUM and similar differential diagnoses [171,172,173] in SLODP, there has been no systematic study of the prevalence of PRCs and remediability after treatment [69]. The high rate (25–50%) of spontaneous reversal to normal cognition [174,175,176,177] and the high risk of progression to dementia in these patients [178], coupled with the reversion-progression course of MUM [179], indicate that a significant number of PRCs are missing in the workup of MCI.

A recently developed model of the diagnostic process developed by the US National Academies of Science, Engineering, and Medicine [180], as well as a universal model for medical diagnostic reasoning [181], suggest that the impediments in the diagnostic workup include difficulties in recognizing patterns among coexisting components of SLODP and associated MUM/BPCs, a faulty diagnostic work-up, and current attitudes that fail to perceive SLODP as an active medical disorder that needs evaluation and treatment.

### 4.1. Difficulty Recognizing Phenomenological and Etiological Patterns in SLODP

Pattern recognition is a skill that is used to identify meaningful regularities among objects, phenomena, events, etc., within a complex environment [182] using complex cognitive processes [183,184,185]. 

SLODP presents multiple coexisting cognitive (e.g., naming deficits [186,187,188]), behavioral (e.g., depression), and functional (e.g., deconditioning) elements [189]. These elements might be syndromes, i.e., they may have a workable differential diagnosis [180] in parallel with the global SLODP dementia syndromes [190].

For effective differential diagnoses for each SLODP subsyndrome, we must be able to recognize the pattern of each syndrome and subsyndrome, as well as every MUM condition.

Difficulty in pattern recognition is the rule in SLODP, as indicated by the high rate of misdiagnosis [191], overdiagnosis [192], and disagreement among different sets of criteria [193,194] for different types of dementia and MCI, as well as failure to identify delirium [195,196], apathy due to depression [197], and geriatric syndromes [190,198].

Pattern recognition in SLODP is crucial for analyzing its phenomenological presentation, subsyndromal trajectory, the relative severity of coexisting subsyndromes, relevant nondegenerative differential diagnoses of specific subsyndromes, prioritization of treatment options, and detection of complications during the disease course.

#### 4.1.1. The Complex System (CxS) Features of SLODP as Major Causes of Pattern Recognition Failure

SLODP is a highly complex disorder [190,198,199,200,201] with multiple complex interactions among its biological and environmental components [202,203,204], including MUM/BPCs [202] and multiple coexistent phenomenological subsyndromes.

**Figure 1:**
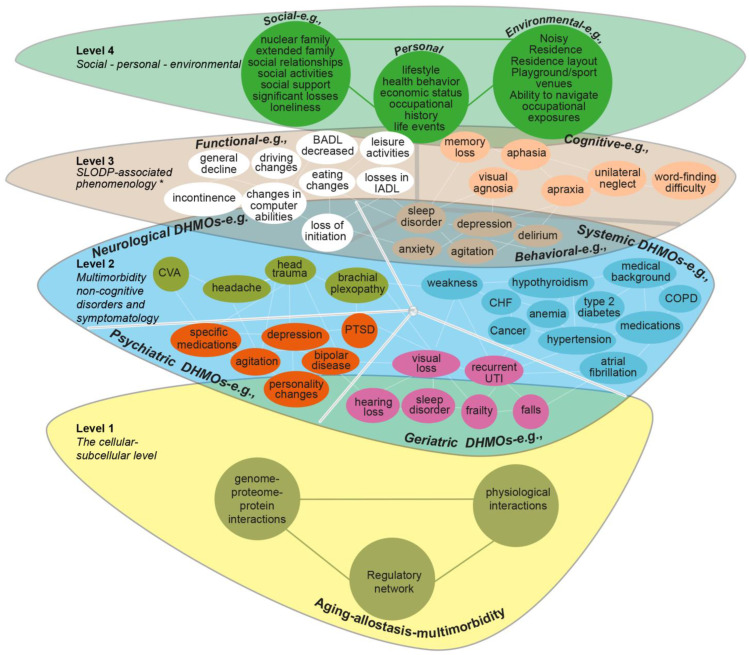
**Human Symptoms Disease Network for SLODP. Level 1:** Cellular–subcellular level, genome–proteome–protein interactions, physiological interactions, regulatory network, aging allostasis multimorbidity. **Level 2:** MUM noncognitive disorders and symptomatology. Neurological, e.g., cerebrovascular accident, brachial plexopathy. Systemic, e.g., weakness, hypothyroidism, anemia. Psychiatric, e.g., posttraumatic stress disorder, bipolar disease, agitation. Geriatric, e.g., visual loss, recurrent UTI, falls. **Level 3:** SLODP-associated phenomenology, e.g., visual agnosia, unilateral neglect: sleep disorder, depression, delirium, general functional decline, sphincter incontinence. **Level 4:** Social-environmental-personal/environmental network, e.g., loneliness/social isolation; environmental, e.g., playground/sports venues; personal/environmental, e.g., health behavior. * SLODP-associated phenomenology—all co-existing behavioral phenomena. **Abbreviations:** BADL, basic activities of daily living; CHF, congestive heart failure; COPD, chronic obstructive pulmonary disease; CVA, cerebrovascular accident; DHMO, disease hierarchical multilevel ontology; PTSD, posttraumatic stress disorder; UTI, urinary tract infection.

Clinical CxS disorders are frequently presented graphically [205,206]. The complex relationships among the clinical diseases themselves [204,207,208,209] are presented as a human symptoms disease network (HSDN). We present the HSDN for SLODP (Figure 1), which includes three complex networks—genome–proteome, disease–clinical MUM states, and social–environmental states [204,210]—and a fourth network that represents the cognitive–behavioral–functional components of the clinical presentation.

The interactions among components of the CxS are organized [202,211] with dynamic features (e.g., pleiotropy, robustness, and rewiring) [202]. Through a “self-organization” process, the infinite number of interactions of the CxS [212] settle “naturally” into “stable” configurations” [213,214] and emergent behavior (EB) [215], which is the whole-system behavior of the CxS [214,216]. In spite of the underlying processes, EB simultaneously develops autonomy [217] and represents a higher-scale superstructure that has a higher adaptive CxS state than the lower-level scale interactions [215,218,219].

EB is perceived as a global state that is difficult to reduce to its causal elemental multiple nondyadic, nonlinear interactive CxS components [214,215].

SLODP is a CxS whose EB is perceived as a slowly progressive gestalt of a global cognitive-behavioral-functional unitarian disorder. *As an EB, SLODP is characterized by the properties of a mega syndrome and not by the properties of any of the components of the lower-scale CxS behaviors from which it emerged* [215,218]. This description of SLODP as a “macro-level” EB shows that its characteristics supersede micro-level SLODP subsyndrome properties [219].

The traditional diagnostic approach to SLODP-EB ignores CxS SLODP subsyndromes and multiple MUM conditions [214,215]. The result is a reductionist dyadic (etiological–phenomenological) paradigm [220] and points to the current parsimonious unifactor-based diagnosis of disease [209] instead of the multifactorial basis of SLODP [208,209,210].

#### 4.1.2. Etiological and Phenomenological Complexity of SLODP

**Figure 2:**
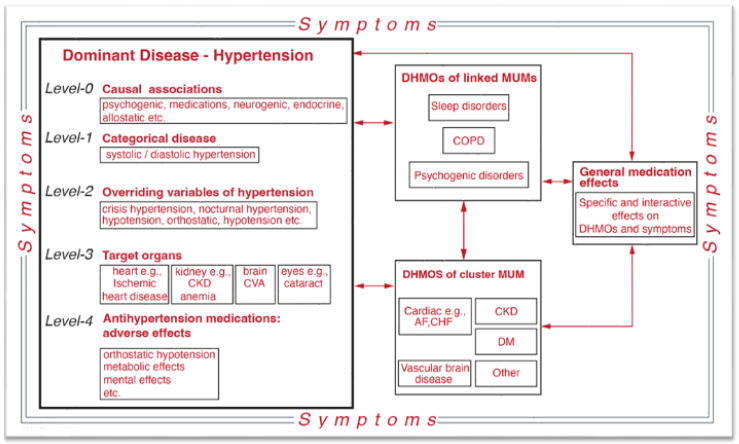
**The disease hierarchical multilevel ontology (DHMO) in SLODP.** The hypertension-associated ontology of SLODP is a multimorbidity-related brain perturbative condition (MUM/BPC) that models knowledge of medical conditions associated with hypertension in a hierarchical structure with wide interactions [279,280,281]. **LEFT:** Five typical levels of hypertension-associated conditions. **RIGHT:** Cluster- (common), linked- (possible), and medication-related DHMOs (see text). **Abbreviations:** AF, atrial fibrillation; BPC, brain perturbative conditions; CHF, congestive heart failure; CKD, chronic kidney disease; CVA, cerebrovascular attack; DHMO, disease hierarchical multilevel ontology; DM, diabetes mellitus; MUM, multimorbidity; SLODP, symptomatic late-onset dementia and predementia.

*Etiological complexity of SLODP.* The etiological complexity of SLODP is related to its multiple clinical and subclinical MUM components [221], suboptimally treated PRCs [222,223], intercurrent events [224,225,226,227,228,229], and other elements of MUM in SLODP detailed above [94,224,230,231,232,233,234]. This results in multiple clinical and molecular environments [202,235], high interconnectivity [236], and immense etiological complexities (Figure 2).

The high etiological complexity and associated EB cause difficulty in recognizing patterns of coexisting MUM conditions (due to masking of concurrent presentations) [233,237]; atypical presentations (e.g., apathetic hyperthyroidism [238] or heart failure with preserved ejection fraction [239]); and blurring of causality relationships [55], which results in attendance to the clinically more dominant conditions while ignoring the less dominant (though significant) PRCs. It also results in failure to prioritize the contribution of the various MUM conditions [86].

*The phenomenological complexity of SLODP*. This results from the dynamic interaction between multiple coexisting cognitive SLODP syndromes and subsyndromes. In addition, there are multiple various combinations of frequent behavioral components (e.g., apathy, sleep disorders, and depression) with high prevalence (20–77% [240,241,242]) and coexistence rates (5–8 coexisting psychiatric symptoms per patient [243] in 56% to 87% of patients, depending on the time of measurement) [241]. The same is true for geriatric co-occurring frequent conditions like frailty, social isolation, hearing loss, and functional disability [244], whose prevalence increases with age to about 50% [245,246,247].

Other features of EB of CxS help in the conceptualization of phenomenological pattern recognition in SLODP. They include the following:

1. Overlapping phenotypic expression of different diseases (e.g., depression and apathy) under psychiatric or neurological conditions [212,248];
2. Cascading failures within a complex system, wherein a failing unit may affect the whole system (e.g., a chronic confusional state aggravating subclinical to clinical cognitive deficits) [249,250];
3. CxS memory effect: the effect of past prior states on the current state (e.g., experiencing a cerebrovascular accident [CVA] or multiple sclerosis at a younger age) [251].
4. Nested components: phenomenological units that require a specific differential diagnosis and workup (e.g., depression in SLODP, fatigue, etc.) [252];
5. Dynamic network of multiplicity (i.e., the existence of many local intra-area interactions and a smaller number of inter-area interactions), which creates isolated cognitive deficits such as severe neglect dyslexia [253] with minimal dementia [252].

In summary, the multi-componential EB SLODP syndrome (Table 1) is perceived as a progressing global cognitive–behavioral–functional **unitarian** disorder. As a rule, the micro-level interacting MUM components are superseded by the EB of SLODP, which blurs them [215,217,254].

Thus, by definition, the mere diagnosis of dementia might be enough for detecting major MUM in SLODP but is not sufficient for identifying specific BPCs and PRCs [255].

Pattern recognition in SLODP is further complicated by the following:

- Clinical heterogeneity of SLODP [256]—the interindividual variability of symptoms. It reflects the number of relevant coexistent symptomatic units, their dominance in the clinical presentation, their phenotypic dimensions (e.g., coarse-like dementia, delirium or fine fatigues events, category-specific anomia, and mild anxiety), and their course. Disregarding the heterogeneity and variability of components of SLODP [18,34,42,256,257,258,259,260,261] prevents full pattern recognition of all of the coexisting phenomenological constituents of SLODP;
- Globalization of coexisting phenomena--the condensation of coexisting major mental syndromes (like dementia, depression, apathy) into a superficially single syndrome that masks its components [262]. This is in contrast to the immediate sharp recognition of patterns in motor-sensory syndromes such as gait disorder (e.g., pyramidal hemiparesis with extrapyramidal and orthopedic syndrome);
- Atypical CxS-related features of psychiatric/neuropsychiatric syndromes. These include the following [263]:
  - Polythetic features—behavioral syndromes that superficially reflect a similar syndromal entity (e.g., depression or systemic sickness behavior [264] or fatigue [265,266,267,268] that are perceived as depressive behavior [263]);
  - Endophenotypes—a specific behavioral disorder that has various internal phenotypes (e.g., several endophenotypes of depression or agitation) [269,270,271,272]. Endophenotypes are almost universal in the cognitive and behavioral syndromes of SLODP [273] and commonly indicate a PRC (e.g., an endocrine disorder or hypoglycemia) [274,275,276];
  - Blurring of the borders between the recognized syndromes and subsyndromes results from the involvement of several clinical-pathological levels in the syndrome. This disturbs the decision about which is the dominant component in the presentation (e.g., blurring the orders between MCI and depressive behavior, where both can cause social withdrawal, thus limiting the ability to decide whether it is caused by more pronounced MCI and minimal depression or the opposite [277,278].

Consequently, it is often difficult to discriminate between a specific SLODP subsyndrome with a distinct differential diagnosis or a component of the global syndrome of SLODP that is included in the global differential diagnosis. For example, distinct optic aphasia in SLODP [279] may represent hypoperfusion ischemia [280,281], a neoplasm infiltrating the parieto-temporal-occipital junction of the left hemisphere [282], or a part of the global SLODP single neuropathology. The same may be true for topographical disorientation [283], which may reflect an autonomous syndrome (e.g., a slowly progressing right parietal neoplasm, hypoperfusion ischemia, or a localized vascular event) or may be part of multifocal vascular dementia [283,284,285].

In summary, pattern recognition is crucial for differential diagnoses of global SLODP and its subsyndromes. Currently, CxS features of SLODP impede it from being fully practiced in the diagnostic workup.

### 4.2. Faulty Diagnostic Workup

Diagnostic medical decisions occur in the context of causal uncertainty [286,287,288,289,290,291] and diagnostic errors [292], especially in a CxS disorder like SLODP. The operation of medical diagnosis is best explained by dual process theories [293,294] that “integrate analytic and non-analytic models of decision making” [180].

Nonanalytic System 1 is fast, heuristic, automatic, highly contextualized, holistic, and unconscious [293,294,295]. It requires little working memory and is triggered by immediate stimuli and overlearned reactions (e.g., emergency conditions such as pulmonary edema, hypovolemic shock, or bleeding from a leg due to a car accident) [181,296]. In such cases, there is an immediate, almost automatic diagnosis and treatment. Associated diagnostic errors are due to cognitive and affective biases [297,298].

Analytic System 2 is much slower. It involves conscious, effortful reasoning guided by critical thinking [180,299,300] and relies heavily on working memory. It uses explicitly gathered information and is rule-based and reflective. It activates hypothetical thinking and counterfactual reasoning. It is rational, consequential, and controlled. The result is a working diagnosis (a single or differential diagnosis) [180]. Analytic System 2 is activated in conditions like chronic anemia or jaundice when there is no clue to the cause. It is essential for a diagnosis of CxS-SLODP [296].

The systems work interchangeably, usually with System 1 over-learned reactions seen first. When it fails, System 2 is activated as an executive control that overrides System 1 [300] when necessary. Because of SLODP’s CxS features [297], System 1 fails to detect core heterogeneous components of the phenomenological and etiological complexity of SLODP, and System 2 is inappropriately activated. Consequently, there is a reflexive reliance on the nonanalytic heuristic System 1. Therefore, there is a failure to create a valid definition of the syndromes and gather MUM-related information. Accordingly, any medical reasoning based on their findings will be inefficient [301] because of the heuristic pitfalls [302,303] and suboptimal decision-making strategies involved [304]. Thus, for most patients with SLODP, the diagnostic process is flawed [301], and no diagnostic alternatives are available [301,302,303,304,305].

A recent model of a diagnostic process emphasizes information gathering and clinical reasoning to reduce uncertainty. Both are deficient in SLODP [180]. Information gathering usually concentrates on macro-domains and ignores fine cognitive and behavioral details (e.g., specific category anomia); focuses on immediate presentation, thereby neglecting trajectory of development; collects superficial data on systemic, psychosocial, and functional background; relies more on neuropsychological testing than on direct clinical evaluation; and does not give enough time for a diagnostic workup.

Clinical reasoning may be hindered because current criteria for dementing disorders do not reflect the complexity of the EB of SLODP, usually ignore multiple coexistent macro syndromes and subsyndromes, and lack an integration step for cognitive-behavioral-functional-geriatric experts.

The faulty process of the diagnostic workup impedes full consideration of the phenomenological and etiological data of SLODP and their integration into a practical diagnosis of SLODP. Thus, the hierarchical organization of the syndrome and its causes and the identification of significant PRCs is not efficient.

### 4.3. Current Attitude Effects on the Diagnostic Workup of SLODP

The CxS features of SLODP call for an intensive diagnostic workup and require high motivation on the part of the diagnosticians; otherwise, it will be flawed.

Regretfully, such motivation is rather lacking in the diagnosis of SLODP [301] for at least two reasons. The first is an “age”-ist approach to disease in older people, especially in those with SLODP [301]. The second is the parsimony, “Occam’s razor” (the unifactorial basis for presenting medical disorders) [306,307]—the accepted diagnostic approach. This does not work for multifactorial conditions such as SLODP, however, and does not motivate further diagnostic efforts after a seemingly single most parsimonious etiology.

The difficulty of pattern recognition, a faulty diagnostic process, and current attitudes toward the aged bring unavoidable diagnostic biases to SLODP, including specific diagnostic biases and errors [180,302,303,304,308,309,310,311]; omission of critical cognitive features (e.g., for a memory-recollection-familiarity nature [312] verbal/nonverbal dichotomy [313], its autobiographical-episodic characteristics [314,315], rate of forgetting [316], or predictive features [317]); superficial integration of concurrent phenomenological units (e.g., the combination of cognitive decline, parkinsonian features, and visual hallucinations is usually diagnosed as Lewy body dementia) [318]. Nevertheless, it may represent subcortical ischemic vascular dementia with Parkinsonian features and a urinary tract infection that requires immediate intervention; premature diagnostic decisions [319] (such as the “Alzheimerization” reflex [320,321]); and overlooking post-diagnostic changes that may indicate PRCs.

The resulting heuristic pitfalls [302,303] and suboptimal decision-making strategies [309] impede the ability of the diagnostician to identify PRCs and result in a bias towards neurodegenerative irremediable diagnoses.

## 5. Proposed Complex System-Based Themes for a Diagnostic Workup of SLODP

Based on the discussion so far, changes in the diagnostic process of SLODP are mandatory. They should include improvement in technical and contextual proficiencies (e.g., appropriate time resources, a non-ageistic attitude, and debiasing). However, a deeper and yielding diagnostic workup is needed. We suggest a conceptual change in the themes of the workup to include the themes of its MUM-BPCs and multilevel phenomenological diagnosis processes.

### 5.1. MUM-BPC Themes

The MUM component of the MUM-BPC theme requires a thorough delineation of all MUM conditions of SLODP (e.g., systemic, geriatric, and neurological diseases [21,244]; psychiatric-psychological syndromes; and functional-social states that jointly affect clinical presentation.

**Figure 3:**
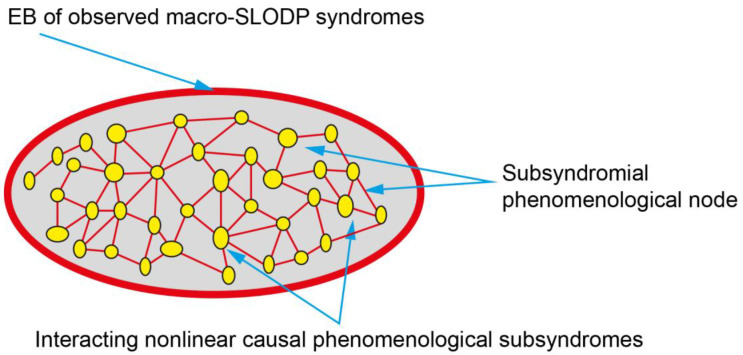
**SLODP as an emergent behavior of phenomenological complexity.** SLODP is a phenomenological complex disorder in which the various phenomenological subcomponents interact as in a complex system. The resulting self-organized Emergent Behavior is the observed dementia macro-syndrome (e.g., Alzheimer’s syndrome, Lewy body dementia syndrome) (red). The causality between the various components and the emergent behavior is nonlinear, which precludes the reducibility of EB to a single component. SLODP is an emergent behavior and, thus, by definition, masks the coexisting subsyndromes (see in the text). **Abbreviations:** EB, emergent behavior; LBD, Lewy body dementia; SLODP, symptomatic late-onset dementia.

The BPCs component is complementary to MUM in the process of identifying PRCs. MUM usually reflects major categories of diseases (such as hypertension or depression) that are not distinct enough for specific treatments in a complex multifactorial ailment like SLODP because they may obfuscate associated treatable BPC conditions and PRCs. The discrete, active, MUM-related BPCs (such as diabetes mellitus-related hypoglycemic events) cause functional and structural brain deficits [77,322,323,324,325,326,327,328,329,330] and specific cognitive changes [331,332]. BPCs may develop in clinical domains that are not part of the predominant MUM [333] (e.g., respiratory difficulty [334] or mild anemia [335] in hypothyroidism) [see DHMO in Figure 3].

The prevalence of BPCs is clearly greater than that of MUM in SLODP, and they affect the patient’s rate of clinical deterioration. BPCs are suboptimally underdiagnosed [336,337,338,339]. The diagnostic workup is relinquished once a MUM has been diagnosed because of an early closure type of error (e.g., nocturnal hypertension or orthostatic hypotension might not be sought when “settling” for hypertension).

BPCs have a high potential for responding to treatment [21]. Their treatment may move SLODP from the list of hopeless diseases and add it back to the list of treatable ones.

### 5.2. Multilevel Phenomenological Diagnosis Theme

**Figure 4:**
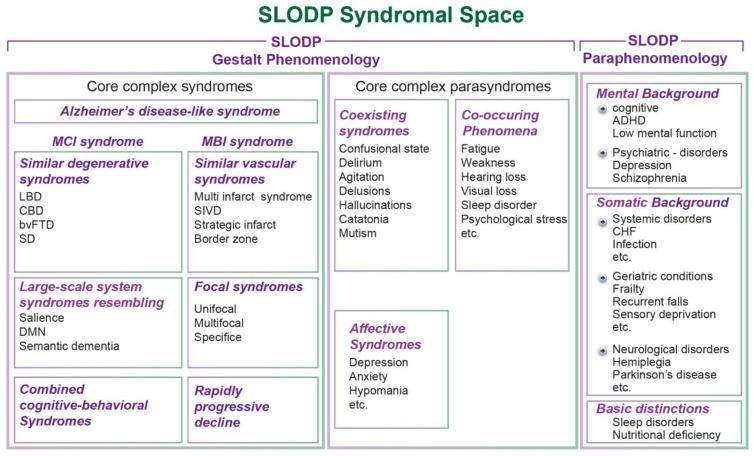
**SLODP syndromal space.** The SLODP syndromal space reflects a gestalt of phenomenological complex system-dependent emergent behavior [346]. The SLODP-gestalt phenomenology means the phenomenological syndromes that frequently develop in parallel in the same period of life and create a conceptual gestalt (law of common fate [347,348,349]), which tends to mask the subsyndromes of SLODP. SLODP gestalt phenomenology (global dominant core syndromes) and para-phenomenology (syndromes that are related to background cognitive and behavioral changes) are quite easily differentiated. SLODP gestalt phenomenology is combined of core-complex gross syndromes (e.g., AD-like syndrome, rapidly progressive dementia syndrome), and core-complex para-syndromes might coexist with the core-complex syndromes (e.g., confusional state, depression, fatigue). Figure 4 is helpful for the multilevel phenomenological diagnosis (see text). **Abbreviations:** AD, Alzheimer’s disease; ADHD, attention-deficit/hyperactivity disorder; bvFTD, behavioral variant frontotemporal dementia; CBD, corticobasal degeneration; LBD, Lewy body dementia; MBI, mild behavioral impairment; MCI, mild cognitive impairment; SD, semantic dementia; SIVD, subcortical ischemic vascular ischemia.

The phenomenological complexity of SLODP indicates the need to map every coexisting syndrome (see Figure 4) to ensure a complete differential diagnosis (see “Implications”).

Based on our experience, we suggest defining the phenomenological syndromal predifferential diagnosis using three graded sequential levels, each of which may point to more discrete subsyndromes with specific differential diagnoses. The three levels are as follows:

- SLODP macro level (SLODPML) syndromes. These include the dominant System 1 macro syndromes of the presenting SLODP organized into their respective central mental spheres: *cognitive* (e.g., SLODP and progressive aphasia), *behavioral* (e.g., depression and agitation), and *functional decline* (e.g., loss of independence and urinary incontinence). The relative contribution of these macro syndromes should be defined (e.g., dominant cognitive and minor depression syndromes vs. the opposite). This creates a conceptual framework of dominant and complementary nondominant syndromes that can help in concentrating on key elements for diagnosis and treatment;
- SLODP intermediate level (SLODPIL) syndromes. These include the sub-global SLODPML syndromes, based on rational System 2 definitions, such as for global SLODP–AD-like, rapidly progressive dementia or MCI and for depressed behavior (SLODPML), depression proper and loss of energy syndrome (e.g., fatigue or sickness behavior). Each SLODPIL syndrome needs a specific differential diagnosis; SLODPIL syndromes should be defined before a differential diagnosis is made in view of the low frequency of purely degenerative dementias and the high frequency of mixed brain dementias [340,341,342]. For example, the SLODPML syndrome of slowly progressive dementia, which frequently elicits a diagnosis of AD, should be defined as an AD-like syndrome with other possible etiological MUM conditions. Another example is a SLODPML syndrome of progressive aphasia, which usually elicits a diagnosis of degenerative PPA but should be differentiated within SLODPIL into specific syndromes, such as a semantic aphasia-like syndrome, nonfluent agrammatic and logopenic-like aphasias, or other aphasic syndromes with a wider differential diagnosis than merely a degenerative disease [343,344];
- SLODP subsyndromal level (SLODPSL) specification includes circumscribed cognitive deficits that are localized to specific small brain areas and are noticeable on a background of SLODPML or SLODPIL (such as optic aphasia or category-specific anomia). These may indicate an additional level of differential diagnosis (such as neoplastic pathologies) as a part of the main global etiology or a coexistent contributing MUM condition.

To identify SLODPSL, providers should be vigilant for finer complaints and atypical combinations of every neurobehavioral domain (e.g., social cognition, salient network features) in addition to more routine ones (e.g., memory, visual agnosia) to avoid the heuristic diagnosis of neurodegenerative disease [345].

Implementing the two new themes in the workup (Figure 1 and Figure 4) can help achieve a full differential diagnosis of SLODP.

## 6. Reducing the Threat of Dementia in Older People—Implications of the Complex-System Features of SLODP

CxS features of SLODP and the suggested diagnostic themes have implications for reducing the threat of SLODP through treatment and prevention. Other implications are for the revision of criteria for SLODP syndromes, development of DMTs and cost reduction, and development of a theoretical model of SLODP (e.g., the MUM-aging complexity pathophysiology [MACP] model).

### 6.1. Clinical Implications

#### 6.1.1. Implications: The Diagnostic Workup of SLODP

##### General Experience

The CxS features of SLODP demand penetration of its EB facade for identification of its building blocks of coexisting phenomenological syndromes and etiological MUM/BPC conditions and their dynamic interactions (Table 1) [207,350].

In our clinic, we use our Intensive Neuropsychogeriatric Evaluation, Treatment, and Prevention (INETAP) protocol, which applies the above-mentioned new themes for information gathering and medical reasoning [180]. A retrospective analysis of a randomly selected cohort of 100 patients with SLODP who completed a 2-year follow-up in our clinic between January 2018 and June 2019 revealed an average of 8.35 PRCs in each patient (Table 2). These patients had previously been evaluated at other institutions, where they received a diagnosis of untreatable SLODP [see Table 2].

Approximately 80% of the patients reported cognitive improvement or stabilization and had improved ability to carry out activities of daily living. We used five essential diagnostic stages.

##### The Five Essential Diagnostic Stages

These five stages include (see Table 3).

*Stage 1: Developing a schema of phenomenological SLODP syndromes and subsyndromes*. The goal of the phenomenological diagnosis of SLODP is to map every coexisting cognitive/behavioral and functional syndrome (see Figure 4) that affects the individual’s comportment. We suggested the above three sequential steps (SLODPML, SLODPIL, and SLODSL; see the subsection titled “Multilevel phenomenological diagnosis theme”). This stage includes optimal localization of cognitive findings in the brain [351].

*Stage 2: Identifying relevant etiological components of MUM/BPC*. This requires the inclusion of every coexisting clinical and subclinical component of the BPC contributing to MUM. DHMO routines are also helpful for accumulating all of the information about all of the coexisting components of the etiology of SLODP (Figure 2).

*Stage 3: Defining the trajectory of the evolving SLODP*. These phenomenological and etiological contingencies are usually masked in presentation. A defined trajectory may provide a better understanding of micropathophysiology and a better way to recognize active DHMO components [352,353] and may suggest additional therapeutic measures for the current condition [342,354].

*Stage 4: Establishing a hierarchy of the syndromal and etiological diagnostic framework*. This framework delineates all active cognitive, behavioral, and functional syndromes, as well as their differential diagnoses and interactions in the current presentation, including a blueprint for

- Presenting distinct coexisting clinical phenomenological syndromes according to their hierarchically relevant dominance in the presentation [351,355,356,357];
- The hierarchy of components of an etiological differential diagnosis of every phenomenological syndrome. For example, a slowly progressive Alzheimer’s-like syndrome may be related primarily to a major vascular component [358] and may be only minimally affected by hearing loss, even though both factors are related to it.

Stage 4 will refer to auxiliary tests, consultations, and at least one follow-up visit before the final diagnosis is made.

*Stage 5: Arriving at a final clinical diagnosis.* At this stage, diagnoses are defined and treatments suggested. The uniqueness of the clinical presentation, dynamic CxS post-diagnosis changes, de-biasing efforts, and allowance of the optimal time for diagnostic workup are achieved [180]. Attention should also be paid to frequent diagnostic dilemmas, such as the cause of functional decline (cognitive or noncognitive), the treatable cause of subacute cognitive deterioration in SLODP patients, the ambiguity of a specific auxiliary finding (e.g., the cause of subcortical ischemic changes) [359,360], and specific causes of BPCs.

##### Clinical Vignettes

Two clinical vignettes are presented as examples of how MUM/BPCs and multilevel phenomenology themes can be included in the diagnostic workup of SLODP.

1. Vignette 1: The significance of an isolated cognitive-specific syndrome in the diagnosis and treatment of SLODP

SP is a right-handed, 77-year-old Holocaust survivor who retired recently from his position as a high-ranking municipality officer. SP was referred to the clinic because of cognitive decline, which was diagnosed as progressive degenerative dementia, most probably nfaPPA.

His medical history included hypertension, ischemic heart disease with angina, and CHF treated with included furosemide, metoprolol, and captopril. No other systemic or neurological deficits were reported.

His physical examination results were noncontributory, except for a supine and standing blood pressure of 95/50 and 90/50, respectively, with an HR of 82 bpm and regular. His motor-sensory neurological examination was negative.

The evaluation was carried out in two stages:

*Stage 1*: Assessment of the cognitive syndrome. The patient and his wife reported a progressive decrease in his ability to concentrate and increased difficulty explaining his thoughts or remembering previous conversations over the last 2 years. He was independent in daily activities except for verbal communication. Other cognitive and behavioral symptoms were not reported.

Behavioral and cognitive examinations revealed a fully cooperative individual with appropriate affect and psychomotor activity. His speech was fluent, but his responses to open questions were incomprehensible. The word sequences made no sense, and the messages could not be understood. Some words sounded like neologisms. SP was fully able to repeat words and sentences and had good verbal comprehension, being able to point and carry out “yes/no” tasks. He named visual objects flawlessly. His memory was preserved, as indicated in episodic verbal and visual tests. Additionally, he was able to perform the specifics of long-term (3-day) orders and items on shopping lists. Other cognitive domains, including insight, were intact.

*Comment on Stage 1*: SP exhibited a progressive transcortical motor aphasia-like syndrome with difficulty in spontaneous language production, preserved comprehension, repetition, and object naming abilities. It did not seem to be degenerative nfaPPA in view of the fully preserved object-naming ability, expression of single words, absence of apraxia of speech, effort of speech, or pauses. A high rate of speech production and length of word clusters was preserved.

The preserved object-naming ability in the face of garbled and incomprehensible sentences suggests a deficit in the functional-level representation of a sentence production model [361]. At this level, nouns and verbs are selected and create a functional argument structure. Because the patient named nouns without difficulty, we added the evaluation of verb naming. The patient named only 2% of verbs correctly compared with 98% of the nouns. Thus, verb anomia was diagnosed as the cause of his sentence production deficit [362].

*Stage 2:* Establishing a differential and etiological diagnosis. A brain CT scan showed heavy calcification of the left dorsolateral frontal branches of the middle cerebral artery. FDG-PET images of the brain showed a localized hypometabolic area in the left posterior middle and inferior frontal gyri, which are considered part of the frontal dorsolateral border zone. A comprehensive etiological workup was negative, except for low blood pressure (mean: 92/47) during 24-hour ambulatory monitoring. A historical review revealed cardiac catheterization because of anginal pain and CHF 2 years ago. His blood pressure used to be between 140 and 150 mmHg (systolic) and 75 to 85 mmHg (diastolic). Post-catheterization afterload blood pressure reduction began and continued.

Based on these findings, he was diagnosed with chronic progressive verb anomia due to hypoperfusion ischemia of the dominant frontal dorsolateral border zone.

Accommodating treatment lowered his blood pressure to approximately 135/70 mm Hg. His speech improved significantly and remained stable for 3 years.

*Comment on Stage 2*: A microphenomenological analysis identified an isolated language syndrome and was helpful in excluding degenerative brain disease. It also pointed to a specific etiology and enabled regenerative treatment.

2. Vignette 2: Multimorbidity and multilevel phenomenology in the evaluation of SLODP.

YA is a right-handed, 78-year-old handyman who also works as a security officer in a cigarette factory. YA was referred to because of 2 years of a gradually progressive decrease in memory and functional changes that were diagnosed as AD. He and his wife complained about his memory difficulties—forgetting meetings, losing important items at home, misidentifying familiar roads while driving, lacking initiative, and being apathetic and a little impulsive. He was independent but now had difficulty managing finances and some home arrangements. He complained of fatigue.

Six months earlier, his MMSE and MoCA scores, measured in another memory clinic, were 26/30 and 22/30, respectively. YA was diagnosed with AD and treated with donepezil and memantine for 6 months without improvement.

His medical history was positive for essential hypertension, dyslipidemia, lower urinary tract symptoms, and vitamin B12 deficiency 10 years ago.

A systemic review revealed mild hearing loss, sleep difficulties (snoring, difficulty in maintaining sleep), fatigue, and excessive daytime sleepiness.

Motorsensory, neurological, and systemic examinations were noncontributory except for morbid obesity, elevated blood pressure (170/85 mmHg), and ischemic oculopathy.

Positive cognitive findings included decreased attention, decreased episodic and delayed recall of verbal memory with preserved recognition, and preserved spatial memory, as well as decreased complex visual memory, working memory, calculating ability, phonemic and semantic word generation, abstraction, and set-shifting ability. He was persevering in multiple-loop tasks. He had preserved naming ability, language functions, semantic knowledge, map knowledge, face and object recognition, and spatial distribution of attention. He was a little impulsive. His MMSE was 28/30, his CDR was 0. 5/3.0, and his GDS was 1/15.

Blood test results were negative except for a high LDL, vitamin D insufficiency, and low-normal vitamin B12. Comprehensive vascular assessment (based on 24-hour ambulatory blood pressure monitoring, 24-hour heart rate monitoring, carotid and vestibular artery ultrasound, and cardiac transthoracic echocardiography) demonstrated uncontrolled systolic and diastolic hypertension during his awake period (mean systolic: 182 ± 27 mmHg—maximum: 230 mmHg; minimum: 146 mmHg. Mean diastolic: 94 mmHg). A brain CT scan showed marked periventricular leukoaraiosis. Polysomnography revealed severe obstructive sleep apnea syndrome (SAS; apnea-hypopnea index [AHI]: 36/h) and nocturnal hypoxemia (O_2_ sat > 90%: 11% of sleeping time). A hearing test showed bilateral sensorineural deficits. His electroencephalogram (EEG) and urinalysis results were negative.

Multidomain MCI was diagnosed. The main parenchymal cause was subcortical ischemic vascular lesions due to uncontrolled hypertension and dyslipidemia, with a major contribution of obstructive SAS and nocturnal hypoxemia. Additional contributing factors included low vitamin B12 and vitamin D levels, as well as decreased hearing.

Treatment of these disorders was recommended. Consequently, the sleep disorder improved significantly, and the cognitive deficit improved and stabilized during the subsequent 2-year follow-up.

*Comment*: This patient presented with progressive memory decline formerly attributed to AD. He received treatment with a cholinesterase inhibitor that did not change the symptomatology. There was no hope for future remediability.

The MUM/BPCs-based evaluation identified major treatable PRCs. Awareness of the frequent role of MUM/BPCs in SLODP prompted a workup with high sensitivity to specific phenomenology and coexisting medical conditions. Indeed, the dysexecutive components of the clinical presentation in the absence of frequent Alzheimer’s disease deficits (e.g., semantic knowledge and naming deficits) and the presence of fatigue pointed towards several distinct phenomenological subsyndromes with their unique differential diagnoses. This clinical workup revealed 7 PRCs, 4 of which were quite major. He was treated for these PRCs, with favorable outcomes that lasted at least over 2 years of follow-up.

#### 6.1.2. Implications for the Treatment of SLODP

Some implications include:

- A complexity-reduction approach: Due to the difficulty in isolating unifactorial dyadic causality, treatment should be designed to reduce the overall syndrome complexity by treating every diagnosed PRC. This will relieve SLODP EB and encourage further exposure of PRCs for treatment;
- Prioritized guidelines for the treatment of MUM components in accordance with the principles of the American Geriatric Society [363], with special attention paid to potential treatment conflicts [236].

Additional implications associated with the CxS aspects of treatment are comprehensiveness (addressing all aspects of treated PRCs, including psychological, psychosocial, and rehabilitative aspects [364]), a patient-centered approach, and a resilience-enhancing approach [365].

#### 6.1.3. Implications for Case Management and Monitoring

The CxS features of SLODP mandate monitoring the completion of multiple MUM-related and general treatment recommendations because any one of them may be an Archimedean point for clinical improvement. Also, any deterioration in the general condition, even if it appears to have already been diagnosed, should be re-diagnosed and managed promptly to prevent permanent decompensation and mental deterioration. Even subtle behavioral changes should mandate a search for a recent active PRC [225].

### 6.2. Implications for the Course and Outcomes of SLODP: The Potential for Remediability

The CXS features of SLODP open new ways to remedy this assumed irreversible, relentlessly progressive condition [9,79,366,367,368,369]:

- Treatment of BPCs [50,370] has a direct effect on the recovery of brain parenchyma and cognitive decline (e.g., ischemic penumbra [371]) and on vascular risk factors (e.g., hypertension and AF) [54,163,372];
- The chaos theory “butterfly effect,” whereby a minute change in a complex system has a large effect elsewhere [373,374,375,376,377], is relevant to SLODP. Stabilization and improvement are seen by treating individual MUM conditions (e.g., mild CHF, subclinical hypothyroidism, or hypertension [345,377,378,379,380,381,382]) or using multidomain interventions [383];
- The CxS-related rewiring potential of the brain [384,385] by rehabilitative activity on top of mere medical treatment [384].

Consequently, because SLODP will usually present with CxS-associated PBCs and PRCs with a paradoxically improved prognosis and improved well-being, settling for stabilization and maintenance of SLODP should be replaced with the search for its remediability.

### 6.3. Implications for the Development of DMT and Cost Reduction

#### 6.3.1. The Potential for an Effective DMT

The current failure for DMT [10] is related to the lack of an identified draggable cause of AD [320], emphasized focus on a “silver bullet” or other small-molecule pharmacotherapeutic agent [10], and low specificity of targets for research (specific molecule due to multiple molecules associated with MUM [386,387,388,389,390,391,392]; core syndromes of degenerative disease due to CxS phenomenological presentation). Effective treatment of patients with MUM/BPC conditions might help overcome these obstacles for DMT in the specific degenerative process.

#### 6.3.2. The Potential for Cost Reduction

A considerable proportion of the economic and societal burden of SLODP is due to MUM/BPCs and their complications [393], which will be significantly lowered by treatment [394].

### 6.4. Implications for CxS Considerations in the Prevention of SLODP

CxS features of SLODP may have crucial effects in achieving better results to prevent SLODP. In medicine, prevention can be primary, secondary, or tertiary and marked by discrete transitions [395]. In contrast, SLODP is a continuous and slowly progressive condition, which makes it difficult to isolate specific preventable cognitive-behavioral events and stages [396,397].

Because of active MUM/BPCs CxS effects prior to and during symptomatic phases [398], we would rather use the terms “presymptomatic” and “parasymptomatic.” These imply the need to prevent any continuous progression of preclinical and clinical disease, not just discrete stages (e.g., MCI to dementia) because it leaves the patient in a better condition. These also imply the need to avoid disregarding every clinical change during the course of the disease, even if it seems unrelated to the primary disorder. In addition, due to the CxS features of SLODP, a wide, multimodal spectrum of preventive measures has potential effects, including social activities and cognitive training [399].

Both presymptomatic and parasymptomatic forms of prevention should include proactive anticipatory prevention measures, such as looking for hypoglycemic events in patients with diabetes [400,401,402]. Patients with hypertension should be pursued for hypotension due to overtreatment, nocturnal hypertension, interactions among medications, and associated conditions (e.g., AF).

In parasymptomatic stages, prevention measures should be periodically revised, updated, and matched with the CxS status at each point along the course. Every time can serve as a launching point for a protocol for preventing new PRCs and in-context conditions.

Prevention should be individually CxS patient-centered and not just based on statistics alone [403]. An ageist attitude should be avoided.

P4 medicine posits that the genomic and “omic” infrastructure should be the basis for disease prevention. Currently, genome-derived proactive programs are not ready for clinical practice [404] and will need to incorporate a CxS-based form of prevention.

### 6.5. Implications for New Criteria for SLODP

Current sets of criteria of dementia [16,372,405,406,407,408] might be inadequate for SLODP because of (1) decreasing specificity with age [377], (2) overlooking the MUM context [370], (3) ignoring the complexity of the syndrome; and (4) disregarding the heterogeneity of presentation. Consequently, they encourage a tendency to define global dementia as degenerative or vascular dementia [12,321,409,410]. This results in inaccurate diagnoses and inadequate clinical and basic research questions.

The CxS features of SLODP should lead to a change in research criteria, thereby promoting clinical benefit and improving research efficiency. New criteria should relate to the following:

- Age groups (onset after 65 years [411], young–old, old–old, and oldest–old populations [412,413]) that run parallel to the rise in MUM, the clinical and pathological heterogeneity [299];
- Specific executive components that take into account CxS features, EB phenomenology, nonlinear causality, and a high probability of PRCs.

The criteria may need specific emphasis according to the clinical phase of the disease.

- At the diagnostic phase, these criteria should:
  - Concentrate on clinical diagnosis and only later be supported by biomarkers of any type;
  - Operationalize the five stages of diagnosis detailed above, including providing practical guidance for acquiring a detailed history, interdisciplinary collaboration among behavior specialists, and a final consensus of joint diagnoses and recommendations.

- At the treatment phase, criteria should provide detailed recommendations for continuous case management to identify and treat emerging BPCs and guidelines for periodic re-evaluations in view of anticipated age-associated increasing MUM:
  - Guide the differential and final diagnosis of each syndrome through an investigation of the role of every coexisting MUM-related BPC;
  - Define terms of performance and time required for a CxS diagnostic workup and develop built-in debiasing procedures (e.g., methods of avoiding early closure and other diagnostic errors) [298].

The current research criteria [18] resulted in prejudice against older people by an ageistic [414] underestimating of their fitness for DMT studies [415], for which it perceives MUM, PBCs, and PRCs to be contraindicated for inclusion [415]. The exclusion of this largest dementia cohort from trials results in skewed data, flawed research designs, and failure to provide accessible patient care. Adequate CxS-based criteria should prevent this inequality and discrimination.

The CxS-based criteria for the workup and treatment of patients with SLODP should yield improvement in DMT research criteria [17] because it diminishes the masking “background noise” of treatable, nondegenerative molecular changes, uses better definitions of cognitive syndromes and contingencies for accurate research questions [42], and increases the spectrum of biological targets and biomarkers for research in SLODP in addition to AD (vascular specific conditions, and other degenerative conditions [12,42,416,417,418,419,420]).

The consideration of clinical and research-based diagnostic criteria for SLODP should be integrated with the primacy of the clinical treatment of every PRC. This will leave research criteria that are sufficient for identifying specific neurobiological constructs for DMT-directed research [17].

### 6.6. Implications for a MUM Aging Combined Pathophysiology (MACP) Model of SLODP

#### 6.6.1. The Need to Revise Models of Dementia in the Elderly

**Figure 5:**
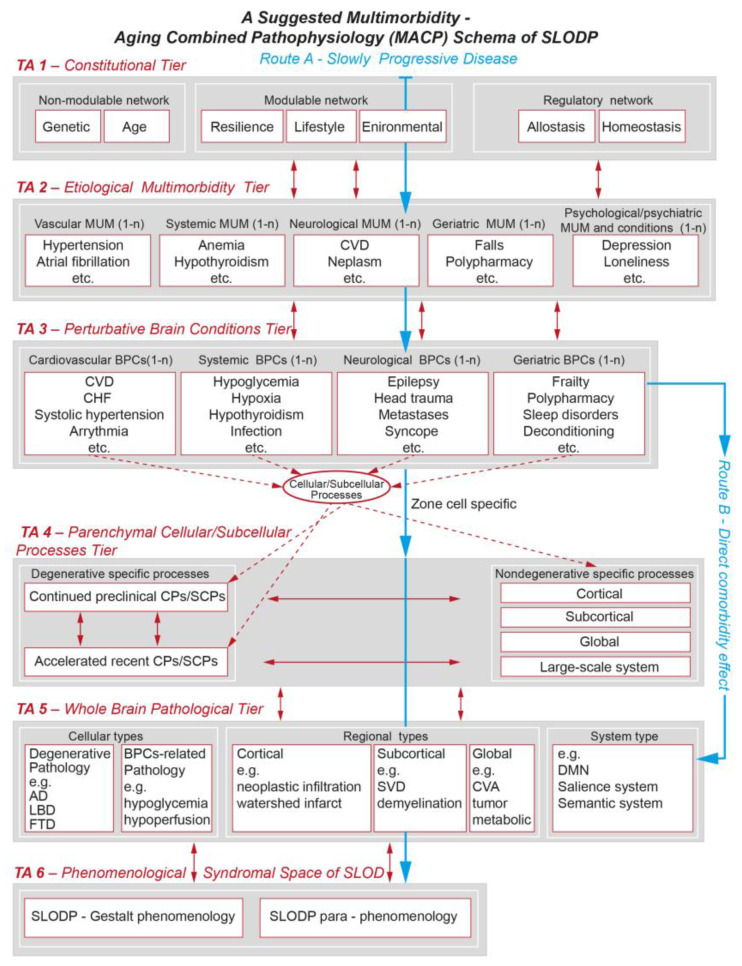
Suggested Multimorbidity-Aging Combined Pathophysiology (MACP) schema for SLODP: a primal sketch. CxS interactions occur between various LA and TA levels and between LAs and TAs. (see text for a full explanation of the model). Each TA demonstrates the components of a slowly progressive disease (Route A). TA 1: Constitutional tier. Networks that affect basal body systems and the initiation and progression of SLODP through priming or direct effects on specific pathophysiological mechanisms. They include non-modulable networks (e.g., genetics [424,425] and aging [425,426,427,428]), modulable networks (e.g., resilience [169], lifestyle [429,430,431,432,433], and environment [404]), and regulatory networks (e.g., allostasis [434] and homeostasis [435]). TA 2: Etiological multimorbidity tier: including vascular, systemic, neurological, geriatric, behavioral/psychiatric, and other MUMs (e.g., air pollution, noise). TA 3: Perturbative states tier. Presents discrete BPS-MUM-related pathophysiological conditions. TA 4: Parenchymal–cellular/subcellular processes tier: Including cell-specific processes and SCPs in the various neuropathological zones. TA 5: Whole-brain pathologies tier: Includes *degenerative cellular pathologies*, brain perturbative condition-(BPC)-related pathologies, *regional pathologies*-cortical, subcortical, global, *and system pathologies* (e.g., default mode and salience networks) Route B-induced pathologies occurring through a direct comorbidity effect. TA 6: Phenomenological syndromal space of SLODP, including SLODP gestalt core phenomenology and paraphrenomenology (see text and Figure 4). Abbreviations: AD, Alzheimer’s disease; AF, atrial fibrillation; BPC, brain perturbative condition; CHF, congestive heart failure; CP, cellular process; CVA, cerebrovascular attack; CVD, cerebrovascular disease; DMN, default mode network; FTD, frontotemporal dementia; LA, longitudinal axis; LBD, Lewy body dementia; MCAP, multimodal characterization of aging pathology combined; MMC, multimodal characterization; MS, multiple sclerosis; MUM, multimorbidity; PTSD, posttraumatic stress disorder; SCP, subcellular process; SLODP, symptomatic late-onset dementia/predementia; SVD, small vessel disease; T2D, type 2 diabetes mellitus; TA, transverse axis.

The current models of dementia are based mainly on AD and vascular dementia [12,365,421] and, thus, exclude SLODP in patients with clinical heterogeneity and MUM [17]. These models are associated with biomolecular constructs [17,422,423] and overlook factors contributing to SLODP [418]. We suggest the MACP model of SLODP, which associates CxS features with its clinical and pathophysiological trajectories (Figure 5). A full discussion of this model will be published separately.

#### 6.6.2. CxS and the MACP Model of SLODP

The MACP model of SLODP reflects the heterogeneous CxS features of SLODP—constitutional, etiological, pathophysiological, pathological, and phenomenological. These are graphically presented on a grid of longitudinal and transverse axes and their interactions (Figure 5). We believe that the MCAP model will facilitate a better understanding of the role of specific and integrated components of MUM-related BPCs in SLODP and lead to better prevention and the development of an effective new DMT.

#### 6.6.3. The MUM Clinical, Pathological, and Cellular CxS Bases of MCAP

##### Indications of the Role of MUM in the Clinical Pathophysiology of SLODP

These indications are (1) a ***parallel***, age-dependent increase in the prevalence of MUM and SLODP [61,62,66]; (2) a dose-response correlation between the ***age-dependent number*** of MUM conditions and the severity of SLODP [436,437,438], the low rate of SLODP in elderly individuals without a significant MUM load [125,437,438]; and (3) an association of age-related MUM with ***acceleration in the progression*** of SLODP along with a greater rate of specific cognitive changes [439].

The lack of an age-related MUM acceleration of the progression of SLODP in YOD [440] suggests two phases of SLODP progression:

*Phase 1* includes preclinical, slowly progressive allostatic and homeostatic processes (spAHP). The allostatic component consists mainly of brain aging processes (such as neurogliovascular micropathology [441] and cellular senescence [390,426]. Energy failure due to mitochondrial dysfunction [442] and the homeostatic component are reactions to early presymptomatic medical conditions, such as the cellular part that reflects the beginning of a disease state [443]. spAHP occurs usually before the age of 65 [443]. Aβ- and tau-associated processes have a dominant role in this phase [444,445].

**Figure 6:**
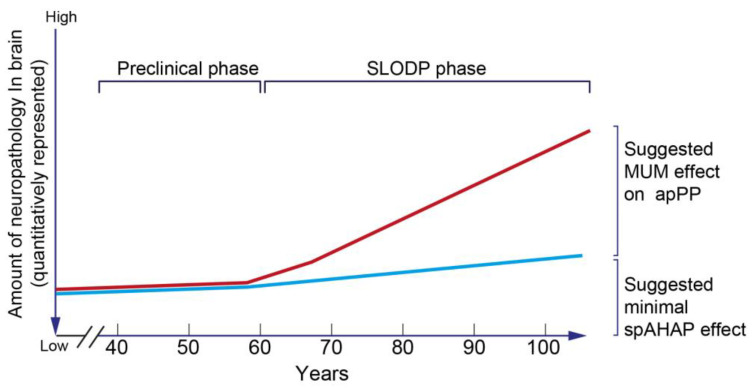
The suggested effect of MUM on the amount of neuropathology in the brain along progression. The blue line represents apAHAP with a hypothesized amount of neuropathology associated only with allostatic and homeostatic aging processes. The red curve represents apPP with a hypothesized progressive increase in neuropathology associated with MUM effects. **Abbreviations:** spAHAP, slowly progressive allostatic and homeostatic aging processes; apPP, accelerated progressive pathophysiological processes.

*Phase 2* includes accelerated progressive pathophysiological processes (acPPPs). The acPPP phase of SLODP has an additional age-associated, MUM-related pathophysiology (Figure 6). We posit that acPPP would be much lower without MUM [40,439,446,447,448].

##### MUM Pathological Complexity in the Heterogeneous Pathophysiology of SLODP

The large number and variety of coexistent MUM/BPCs in SLODP (e.g., vascular disorders, sepsis, lung disease, and many other conditions [270,449,450,451,452,453,454,455,456,457,458] cause specific neuropathological CxS with additive or synergistic inter-effects and result in heterogeneous pathological processes and clinical cognitive presentations.

##### The Effect of MUM on the Multi-Pathophysiological Channels of AD Components in SLODP

1. AD pathological changes do not reflect a single pathophysiology.

AD lesions occur very frequently in geriatric patients with dementia [459] and traditionally have led to the conclusion that AD is the main cause of dementia in this population [1,10]. This might not be true, however, because

- Aβ and tau are not specific to SLODP and are present in other neurological conditions [460,461,462] and geriatric conditions [463,464,465,466];
- There is a wide range of MUM/BPC conditions that are highly prevalent in SLODP and associated with an increase in amyloid and tau in the brain, including hypertension [467], type 2 diabetes mellitus [468], hyper- and hypoglycemia [469], hypoxia [470], and sepsis [471], as well as other states [150,472,473,474,475,476,477,478,479,480,481,482,483,484];
- The tau/Aβ pathology of AD may be the consequence—and not the cause—of the pathophysiological process, especially in older people [459].
- MUM/BPCs have a role in aging processes such as cellular senescence [390].

2. Activation of multiple subcellular processes by MUM/BPCs in AD and SLODP

Coexisting MUM/BPCs activate multiple pathophysiological subcellular processes in AD/SLODP and SLODP. We propose that the MUM/BPC Aβ/tau pathology in SLODP results from the general physiological role of Aβ and tau in normal physiological and pathological subcellular processes (SCPs). This is based on the following considerations:

- MUM/BPCs associated physiologically with Aβ/tau that regulated SCPs in the presence of abnormal Aβ and tau have physiological roles in a wide spectrum of normal molecular essential SCPs [444,485], such as energy metabolism, adaptive cellular stress responses, autophagy [486], intracellular degradation pathways, cytoskeleton dynamics, organelle organization, and neurotransmission, as well as cellular communication [487], regulation of axonal transport [488], neurogenesis, synaptogenesis [489,490], response to injury [444], mitochondrial mobility [491], and more (for a review, see Wang and Holtzman, 2020) [444];
- MUM/BPCs associated with Aβ/tau pathophysiologically regulate d SCPs in SLODP [136,151,444]. MUM/BPCs in SLODP (such as hypoxia [136,137], sepsis [492], hyperglycemia [493], hypoglycemia [140], and hypothyroidism [141,494]) cause deviations from the normal physiological roles of Aβ/tau and early changes that reflect homeostatic failure [152,421,495]. The spectrum of SCPs that involve these Aβ/tau processes in SLODP is wide [496,497] and includes anti-inflammatory processes [498,499], insulin resistance [500], organelle-specific processes (e.g., in mitochondria and synapses) [501,502], and physiological pathways [503,504,505]. Amyloid degradation and tau phosphorylation pathways are also affected. Because many of these processes are involved in AD neuro-pathology [506], the result is a MUM/BPC-related accumulation of AD pathology in SLODP.

3. The effects of MUM/BPCs on SCPs in SLODP are distinct:

MUM/BPCs are associated with distinct SCPs even if there is a similar pathobiochemical state (for example, in hypoxic COPD and CHF [507,508]). Other examples of distinct brain SCPs are seen in obesity [150], oligemic hypoperfusion [509], and systemic causes of a patient’s demise [510,511]. The specific SCPs involved in each individual depend on the profile of MUM/BPC disorders and their relative effect on the activation of specific SCPs [512,513] and the individual’s constitutional background.

##### The MUM/BPC Syncytium of SCPs in SLODP

The plethora of SCPs in MUM/BPCs creates a highly complex syncytium of molecular interactions in SLODP. A syncytium framework is based on (1) the plethora of omic, genetic, and biochemical findings in SLODP that cannot be explained by a parsimoniously coherent mechanism [10]; (2) the heterogeneity of cellular and SCP neuropathological changes in AD in SLODP [514] (e.g., the specific cellular units involved [514,515,516]; white matter elements [516,517]); (3) non-tau/Aβ pathophysiological mechanisms (as seen in chronic hypoperfusion [518] and additional frequent medical states [450,516,517,519]); and (4) secondary pathological effects (e.g., BBB and the capillary changes in hypertension [516] and type 2 diabetes mellitus [322]) or the spreading of tau or Aβ proteins in AD [520].

The variability of the MUM/BPCs syncytial cellular/SCPs CxS creates coexisting microenvironments with resulting heterogeneous molecular cascades. This explains the frequent existence of polymorphisms of Aβ/tau ultramicroscopic structures [521,522,523] and the multiple types of filaments in degenerative conditions such as taupathies [524,525,526].

We posit two forms of Aβ/tau pathology in SLODP: (1) polyformic early Aβ/tau forms, which are detected by advanced methods and are formed by early specific syncytial microenvironments and (2) late unified Aβ/tau forms that can be detected by current conventional methods and are the final end product of more advanced SCPs [139,388,390,527,528,529,530,531,532].

#### 6.6.4. The MACP Model of SLODP

- The demands from the model

These include (1) single-disease MUM pathophysiological cascades and (2) parallel tiers that allow complex direct and indirect interactions among the MUM cascades. The model should express the complexity of SLODP and enable its pathological and clinical heterogeneity.

- The grid features of the MACP model

The CxS features of SLODP can be organized on a grid on which (1) the longitudinal axes represent MUM pathophysiological cascades and (2) five basic transverse axes are placed to represent the interactions among coexisting longitudinal axes of MUM/BPCs [Figure 5]. Usually, there are several coexisting longitudinal axes. Even a single MUM/BPC longitudinal axis can divide into a few pathophysiological routes (e.g., an ischemic stroke with complete infarct in a specific arterial territory and hypoperfusion ischemia in another [19]; the combination of cortical effects and the hypothalamic-pituitary axis of hypoxemia [533]; the effects of hypothyroidism on a default mode network and associated brain area, including the olfactory bulb [450,534] and obstructive sleep apnea syndrome with specific areas of gray matter loss [535]). The number of the various interactions and the complexity increase, respectively.

- Core functional tiers of the CAMP model (see Figure 5):

These are the six basic transverse axes interaction tiers crossing coexisting longitudinal cascades of MUM/BPCs:

*The constitutional components tier.* This includes networks of basal body systems that affect SLODP either through indirect priming or direct effects. They are associated with specific genome-proteome and protein reactions [208,429]. The main constitutional elements are non-modulable, modulable, and a regulatory network (see [Figure 5]). These elements affect the early allostatic and homeostatic stages of the spAHP process and are later affected by MUM and BPCs.

*Etiological MUM tier.* Covers diseases contributing to or functioning as risk factors in the pathophysiology of SLODP, including vascular (e.g., hypertension, CHF, AF), systemic (e.g., T2D, hypothyroidism, anemia, neoplastic disorder, specific medical or surgical background, medications), neurological (noncognitive conditions, e.g., epilepsy, CVA), geriatric (e.g., frailty, malnutrition, sleep disorder, polypharmacy), behavioral/psychiatric (e.g., depression, PTSD), and other MUMs (e.g., air pollution, noise).

*Perturbative states tier.* Presents discrete BPS-MUM–related pathophysiological conditions that cause a deviation in the function of the brain or other body systems that affect the brain from the basal state. These are well-differentiated conditions with specific differential diagnoses that respond to specific medical treatments. The etiological MUM and the perturbative tiers interact to cause an integrated global effect on the brain (e.g., on general energy metabolism [536] or entropy) [537], in addition to the MUM/PBC-specific effects. Their impact increases with progressing age and SLODP.

*The parenchymal subcellular processes (SCPs tier)*: Includes cell-specific processes encompassing continued preclinical SCPs (subcellular processes, e.g., changes in the neuroglial vasculature or changes in chronic hypoxia changes), recent accelerated SCPs (e.g., hypoperfusion, hypoglycemia), and zone-specific processes, e.g., cortical processes (cortical infarction, laminar necrosis, neoplastic infiltration, etc.), subcortical processes (subcortical infarctions, small-vessel disease, multiple sclerosis [MS], etc.), and global processes (CVA, space-occupying neoplasms, chronic subdural hematoma, etc.).

*Whole-brain pathologies tier.* The variety of multiple lesions results from multiple disease-associated structural pathologies (Figure 5) [341,538]. The distribution of lesions varies among patients and includes *degenerative cellular pathologies* (Alzheimer’s disease [AD], Lewy body disease [LBD], frontotemporal dementia [FTD], etc.) and brain perturbative condition-(BPC)-related pathologies (lesions resembling those seen after hypoglycemia or metabolic, drug-induced, or hypoperfusion lesions); *regional pathologies*—cortical (neoplastic infiltration, cortical infarctions, or microbleeds, etc.), subcortical (due to small-vessel disease or cobalamin deficiency), and global (space-occupying neoplasms, chronic subdural hematoma, etc.); *systemic pathologies*—caused by hub systems (default mode in hypothyroidism [539] and salience networks in the behavioral variant of FTD [536], semantic systems, etc.); and *Route B-induced pathologies* occurring through a direct comorbidity effect.

*The phenomenological syndromal space of SLODP* includes SLODP gestalt core phenomenology and paraphrenomenology. This phenomenological stratum includes the observable components of the syndromal space of SLODP, its gestalt phenomenology, and its paraphenomenology [see text and Figure 4].

CxS interactions occur between various LA and TA levels and between LAs and TAs.

The MACP scheme represents the multifactorial pathophysiology of SLODP. The schema may prompt the establishment of an integrated strategic approach using defined targets and stimulate the exploration of new research avenues and treatment options. We referred to the schema as a primal sketch akin to its use in visual cognition [540] so that the model continuously revised as additional knowledge is accumulated as we hope.

## 7. Summary and Conclusions

SLODP is a devastating, highly prevalent condition in older patients for which there is currently no treatment. Perceiving SLODP as irreversible and normal at an advanced age, patients and their caregivers have been left to suffer progressively for years without any help from the medical community. There is an urgent need for a new disease-modifying, regenerative, clinically relevant strategy for patients with SLODP that is directed to the pathophysiology of the condition and will significantly alleviate symptoms, improve daily functioning, relieve the burden for caretakers, and reduce socioeconomic costs. In our experience, the best immediate approach to SLODP is to treat the PRCs that are identified among coexisting multiple MUM/BPCs in the SLODP population because most of them are medically treatable. As mentioned previously in this article, MUM/BPC effects seem to be causal rather than correlational.

In the face of a very high rate of MUM/BPCs in patients with SLODP, it is hard to accept the low rate at which PRCs are identified and the very low rate of reversibility and remediability. According to a recently designed universal model for diagnostic reasoning, this is mainly because of (1) difficulty in recognizing patterns of coexisting cognitive/behavioral subsyndromes and multiple etiological interacting components of the SLODP EB that impedes the diagnosis; (2) a faulty diagnostic workup and faulty medical reasoning due to built-in imperfect information-gathering methods, which result in an impaired combination of heuristic and rational effortful reasoning in the diagnostic workup; and (3) ageism, which makes it difficult to perceive SLODP as an active medical disorder that needs evaluation and treatment.

To overcome these drawbacks, we proposed CxS-based themes as conceptual bases for the diagnostic workup:

- The MUM-BPC themes, according to which MUM usually reflects major categories of diseases, such as hypertension, and BPCs are discrete, active, MUM-related conditions such as diabetes mellitus-related hypoglycemic events. In addition to MUM conditions, BPCs are much more prevalent and usually treatable, which might stabilize or improve SLODP. Their diagnosis is very often obfuscated by MUM or not looked for because currently, medical charts rarely, if ever, enumerate every coexisting BPC;
- The multilevel phenomenological diagnosis theme suggested for recognition of all coexisting syndromal and subsyndromal components of SLODP consists of a structured multilevel phenomenological penetration of the global macro-syndrome EB of SLODP. Levels include SLODPML (e.g., dementia-like syndrome), SLODPIL (e.g., AD-like, SIVD-like, or rapidly progressive dementia), and SLODPSL (e.g., category-specific anomia or optic aphasia).

The suggested new CxS-based diagnostic approach to SLODP prevents the omission of existing syndromes and etiologies, widens the differential diagnosis, and enriches the etiological possibilities. This results in the identification of several PRCs per patient.

Additional implications of conceptualizing SLODP as a CxS are remediability, cost reduction, effective prevention, the need for new executive criteria, a pathophysiological understanding of SLODP, and research and development of disease-modifying treatments.

## 8. Limitations and Future Directions

The major limitations of our approach are, among others, its single (though large) multi-professional clinic basis, costs, and mandate for a change in concepts and paradigms in almost every area related to dementia.

Because the results of the approach might be very valuable, it may be essential to embark on several randomized controlled clinical trials in a few centers and carry them out simultaneously. Such studies should include clinical and neurobiological components. If the results are positive, then the next step will be to carry out studies designed to improve efficiency and lower costs. Only then will it be possible to investigate the full effectiveness of the process mentioned herein.

## Figures and Tables

**Table 1:** **Clinical relevant phenomenological interactions of Complex-system SLODP.** The table presents a wide spectrum of components of CxS of SLODP, including Nodal, Internodal, and Context sensitivity factors.

The Clinical Relevant Interactions between Nodal Components in the Phenomenology of SLODP		
a. Nodal and basal components	1. heterogeneity and coexistence in a single patient	i. primary phenomenological components, specific core SLODP phenomena, e.g., cognitive (amnesic syndrome optic aphasia), psychiatric (apathy, agitation) functional (changes in personal hygiene, difficulty in running the house)
		ii. modulating components of phenomenological presentation, specific associated phenomena that may affect core ones, e.g., systemic (fatigue, medical stress-dyspnea), geriatric (frailty, hearing loss), neurological (dysarthria, gait disorder), psychological (mourning, psychological stress), social (social isolation), environmental (new living place, noise)
	2. high variability of in the frame-features of the phenomenological components	i. behavioral/cognitive frame-e.g., gloal or domain specific; mixed phenomenological presentations (memory, affect, language, function) mixed intra-domain components (coexiating language syndromes-anomia, sentence production, comprehension) relative severity of global and coexisting deficits
		ii. temporal frame-e.g., onset (chronic, subacute, acute, hyperacute) course (progreesive, transient, relaping-remitting, fluctuative, stepwise) relative onset and course (subacute/acute on chronic progressive)
	3. Instability of the cognitive/behavioral syndrome	e.g., temporal fluctuations(global syndrome/specific parts instability of phenomenology
	4. background effects	i. signal/noise effects, e.g., blurred distinction between background conduct and recent changes para-SLODP changes (aging, social, non-SLODP functional loss, deconditioning) minimal behavioral impairment
		ii. culture effect-might appear to lower function in the elderly in spite of preserved cognitive abilities, e.g., may lower test findings in spite of preserved cognition
		iii. Silent cognitive deficits-e.g., early “transparent” prefrontal disease very slowly progressive changes
		iv. a limited and routine span of life functioning that mask ADL challenges
	5.observer factors	i. low-awareness to changes states-that prevent an adequate report e.g., anosognosia, family/community factors
		ii. age-istic and stigmatic approach
		iii. the polythetic nature of neuropsychiatric disease
b. interactions between the multiple nodal components of SLODP-CxS	1. between nodal components	
	2. contextual- modulating effects—(e.g., emotional or social states)	
	3. brain hard-wired network regulatory effects, e.g., pain, hearing loss	

**Table 2:** Potentially remediable conditions in a sample of 100 SLODP patients. **Abbreviations**: ADHD, attention-deficit/hyperactivity disorder; AF, atrial fibrillation; COPD, chronic obstructive pulmonary disease; DM, diabetes mellitus; ICA, internal carotid artery; IHD, ischemic heart disease; PMLS, periodic leg movement of sleep; SAS, sleep apnea syndrome; SLOD, symptomatic late-onset dementia.

**Vascular brain changes**		**Systemic relevant disorders**		**Sleep disorders**	
Clinical/Significant- imaging changes	69	Anemia/polycytemia	11	SAS significant	58
ICA significant disease	5	Coagulation disorders	8	Nocturnal hypoxemia	28
**Cardiac risk Conditions**		COPD	12	Insomnia-nocturia due	9
IHD	17	B12 deficiency/- partially treatment	33	Insomnia-no etiology	8
Congestive heart-failure	11	Thiamin deficiency	2	PMLS	4
Bradyarrythmias	17	Active dysthyroidism	15	Parasomnia- significant	1
AF	18	Medication effects	35		
Aortic Stenosis	5				
**Vascular risk factors**		**Affective disorders**		**Other**	
Uncontrolled- hypertension	76	Depression	46	Hearing loss	50
Hypotension	43	Anxiety	27	Visual untreated deficits	12
Orthostatic hypotension	24	Acute/ subacute stress	43	Deconditioning	24
High hyperlipidemia	86	Bipolar disorder	1	Adult ADHA	10
Uncontrolled DM	51			Alcoholic disorder	3
Hypoglycemic events	6			Caffeine effect	2
Morbid Obesity	7				

**Table 3:** Suggested Stages of Diagnosis of SLODP.

Stage of Evaluation	Goal	Examples
Stage 1. Phenomenological definition		
1.1. Macrolevel (SLODPML) coexisting syndromes	- Detecting dominant immediate impression of observed cognitive-behavioral-functional global macro-syndromes of the presentation - Ordering according to dominant and complementary gross roles in the presentation	- Cognitive-dementia-like syndrome, progressive aphasia, memory deficit, naming deficit, confusional state - Behavioral-depressed behavior, agitation, psychosis, visual hallucinations, anxiety - Functional-social behavior, home duties, hearing loss, lack of initiative
1.2. Intermediate level (SLODPIL) syndromes	- Preparing sub-global specific cognitive-behavioral-functional syndromes for focused differential diagnosis	- Dementia-like syndrome - AD-like syndrome - Rapidly progressive dementia - Depression-like behavior - depression-proper - sickness-behavior - fatigue
1.3. Subsyndromal level (SLODPSL) subsyndromes	- Identifying circumscribed cognitive deficits, which are localized to small areas in the brain and are noticeable on the background of 1.1 and 1.2 - Achieving a wider MUM-related differential diagnosis	
Stage 2. Identifying relevant MUM/BPC etiological components	- Gathering every coexistent relevant clinical and subclinical component of MUM/BPCs - Creating a matrix for relating phenomenological syndromes to potential etiologies	- CHF, subclinical Hypothyroidism, past history of malignancy weight loss, relevant medications
Stage 3. Defining the trajectory of the evolving SLODP	- Drawing the trajectories of phenomenological deficits and medical events from the onset - Identifying, in addition to stage 2, of furthermore active etiological states - Maximizing phenomenological etiological contingencies - To enhance etiological diagnosis and treatment options	- Changes of cognitive-behavioral-functional states in parallel with sepsis events, myocardial infarction, and hypoglycemic events - Along the course of the Evolving syndrome
Stage 4. Establishing a hierarchy of syndromal and etiological data for diagnostic framework	- Analyzing coexistent phenomenological syndromes according to their hierarchical relevant dominance and their specific etiological differential diagnosis - Integrating all sources of information - Accomplishing auxiliary tests	- Dominant slowly progressive AD-like with major vascular component and minimal hearing loss - Sleep Apnea Syndrome with chronic obstructive pulmonary disease
Stage 5. Arrival at final clinical diagnosis	- Deciding upon treatment	- According to found PRCs

## Data Availability

Data sharing is not applicable to this article as it is an opinion/review article, and no new data were fully created or analyzed in it.

## Abbreviations

acPP	accelerated pathophysiological progressive process
AF	atrial fibrillation
BADL	basic activities of daily living
BBB	blood-brain barrier
BPC	brain perturbative condition
CAMP	complexity-based aging-multimorbidity pathophysiology
CHF	congestive heart failure
COPD	chronic obstructive pulmonary disease
CVA	cerebrovascular accident
CxS	complex system
DHMO	disease hierarchical multilevel ontology
DMT	disease-modifying treatment
EB	emergent behavior
FTD	frontotemporal dementia
HDN	human disease network
HF	heart failure
HSDN	human symptoms disease network
INETAP	Intensive Neuropsychogeriatric Evaluation Treatment and Prevention
LBD	Lewy body dementia
MCI	mild cognitive impairment
MUM	multimorbidity
nfaPPA	nonfluent agrammatic PPA
PPA	primary progressive aphasia
PRC	potentially remediable condition
SCP	subcellular process
SLODP	symptomatic late-onset dementia and predementia
SLODPg	symptomatic late-onset dementia and predementia—global
SLODPil	symptomatic late-onset dementia and predementia—intermediate-level
SLODPml	symptomatic late-onset dementia and predementia—macro level
SLODPsl	symptomatic late-onset dementia and predementia—subsyndromes
spAHAP	slowly progressive allostatic homeostatic aging process
UTI	urinary tract infection
YOD	young-onset dementia

## References

[1] Khachaturian A.S., Hayden K.M., Mielke M.M., Tang Y., Lutz M.W., Gold M., Kukull W.A., Mohs R., Gauthier S., Luis Molinuevo J. (2018). New thinking about thinking, part two: Theoretical articles for Alzheimer’s and dementia. Alzheimer’s Dement..

[2] Lopez O.L., Kuller L.H. (2019). Epidemiology of aging and associated cognitive disorders: Prevalence and incidence of Alzheimer’s disease and other dementias. Handbk. Clin. Neurol..

[3] Dinesh D., Shao Q., Palnati M., McDannold S., Zhang Q., Monfared A.A.T., Jasuja G.K., Davila H., Xia W., Moo L.R. (2023). The epidemiology of mild cognitive impairment, Alzheimer’s disease and related dementia in U.S. veterans. Alzheimer’s Dement..

[4] Dauphinot V., Potashman M., Levitchi-Benea M., Su R., Rubino I., Krolak-Salmon P. (2022). Economic and caregiver impact of Alzheimer’s disease across the disease spectrum: A cohort study. Alzheimer’s Res. Ther..

[5] El-Hayek Y.H., Wiley R.E., Khoury C.P., Daya R.P., Ballard C., Evans A.R., Karran M., Molinuevo J.L., Norton M., Atri A. (2019). Tip of the iceberg: Assessing the global socioeconomic costs of Alzheimer’s disease and related dementias and strategic implications for stakeholders. J. Alzheimer’s Dis..

[6] Nichols E., Steinmetz J.D., Vollset S.E., Fukutaki K., Chalek J., Abd-Allah F., Abdoli A., Abualhasan A., Abu-Gharbieh E., Akram T.T. (2022). Estimation of the global prevalence of dementia in 2019 and forecasted prevalence in 2050: An analysis for the Global Burden of Disease Study 2019. Lancet Public Health.

[7] Gjøra L., Strand B.H., Bergh S., Borza T., Brækhus A., Engedal K., Johannessen A., Kvello-Alme M., Krokstad S., Livingston G. (2021). Current and future prevalence estimates of mild cognitive impairment, dementia, and its subtypes in a population-based sample of people 70 years and older in Norway: The HUNT study. J. Alzheimer’s Dis..

[8] Clarfield A.M. (2003). The decreasing prevalence of reversible dementias, an updated meta-analysis. Arch. Intern. Med..

[9] Cummings J., Feldman H.H., Scheltens P. (2019). The “rights” of precision drug development for Alzheimer’s disease. Alzheimer’s Res. Ther..

[10] Golde T.E. (2023). Disease-modifying therapies for Alzheimer’s disease: More questions than answers. Neurotherapeutics.

[11] Lee E.C., Hong D.Y., Lee D.H., Park S.W., Lee J.Y., Jeong J.H., Kim E.Y., Chung H.M., Hong K.S., Park S.P. (2022). Inflammation and rho-associated protein kinase-induced brain changes in vascular dementia. Biomedicines.

[12] Beishon L., Panerai R.B. (2021). The neurovascular unit in dementia: An opinion on current research and future directions. Front. Aging Neurosci..

[13] Magrath Guimet N., Zapata-Restrepo L.M., Miller B.L. (2022). Advances in treatment of frontotemporal dementia. J. Neuropsychiatry Clin. Neurosci..

[14] Livingston G., Sommerlad A., Orgeta V., Costafreda S.G., Huntley J., Ames D., Ballard C., Banerjee S., Burns A., Cohen-Mansfield J. (2017). Dementia prevention, intervention, and care. Lancet Comm..

[15] Blotenberg I., Hoffmann W., Thyrian J.R. (2023). Dementia in Germany: Epidemiology and prevention potential. Dtsch. Ärzteblatt Int..

[16] Jack C.R., Bennett D.A., Beknow K., Carrillo M.C., Dunn B., Haeberlein S.B., Holtzman D.M., Jagust W., Jessen F., Karlawish J. (2018). NIA-AA research framework: Toward a biological definition of Alzheimer’s disease. Alzheimer’s Dement..

[17] Dubois B., Villain N., Freson G.B., Rabinovici G.D., Sabbagh M., Cappa S., Bejanin A., Bombois S., Epelbaum S., Teichmann M. (2021). Clinical diagnosis of Alzheimer’s disease: Recommendations of the International Working Group. Lancet Neurol..

[18] Tellechea P., Pujol N., Esteve-Belloch P., Echeveste B., García-Eulate M.R., Arbizu J., Riverol M. (2018). Early-and late-onset alzheimer disease: Are they the same entity?. Neurología.

[19] Love S., Perry A., Ironside J., Budka H. (2018). Greenfield’s Neuropathology: Volume 1.

[20] Loscalzo J., Fauci A.S., Kasper D.L., Hauser S.L., Longo D.L., Jameson J.L. (2022). Harrison’s Principles of Internal Medicine.

[21] Bevins E.A., Peters J., Léger G.C. (2021). The diagnosis and management of reversible dementia syndromes. Curr. Treat. Options Neurol..

[22] Watts G. (2012). Why the exclusion of older people from clinical research must stop. BMJ.

[23] Devineni B., Onyike C.U. (2015). Young-onset dementia epidemiology applied to neuropsychiatry practice. Psychiatr. Clin. N. Am..

[24] Hendriks S., Peetoom K., Bakker C., Van Der Flier W.M., Papma J.M., Koopmans R., Verhey F.R., De Vugt M., Köhler S., Withall A. (2021). Global prevalence of young-onset dementia: A systematic review and meta-analysis. JAMA Neurol..

[25] Chiari A., Vinceti G., Adani G., Tondelli M., Galli C., Fiondella L., Costa M., Molinari M.A., Filippini T., Zamboni G. (2021). Epidemiology of early onset dementia and its clinical presentations in the province of Modena, Italy. Alzheimer’s Dement..

[26] Prince M., Bryce R., Albanese E., Wimo A., Ribeiro W., Ferri C.P. (2013). The global prevalence of dementia: A systematic review and meta-analysis. Alzheimer’s Dement..

[27] Ferri C.P., Prince M., Brayne C., Brodaty H., Fratiglioni L., Ganguli M., Hall K., Hasegawa K., Hendrie H., Huang Y. (2005). Global prevalence of dementia: A delphi consensus study. Lancet.

[28] Andreu-Reinón M.E., Huerta J.M., Gavrila D., Amiano P., Mar J., Tainta M., Ardanaz E., Larumbe R., Navarro C., Colorado-Yohar S.M. (2020). Incidence of dementia and associated factors in the EPIC-Spain dementia cohort. J. Alzheimer’s Dis..

[29] Cao Q., Tan C.C., Xu W., Hu H., Cao X.P., Dong Q., Tan L., Yu J.T. (2020). The prevalence of dementia: A systematic review and meta-analysis. J. Alzheimer’s Dis..

[30] Gelber R.P., Launer L.J., White L.R. (2012). The Honolulu-Asia Aging Study: Epidemiologic and neuropathologic research on cognitive impairment. Curr. Alzheimer Res..

[31] Yang Z., Slavin M., Sachdev P. (2013). Dementia in the oldest old. Nat. Rev. Neurol..

[32] Nelson P.T., Brayne C., Flanagan M.E., Abner E.L., Agrawal S., Attems J., Castellani R.J., Corrada M.M., Cykowski M.D., Di J. (2022). Frequency of LATE neuropathologic change across the spectrum of Alzheimer’s disease neuropathology: Combined data from 13 community-based or population-based autopsy cohorts. Acta Neuropathol..

[33] Panegyres P.K. (2021). The clinical spectrum of young onset dementia points to its stochastic origins. J. Alzheimer’s Dis. Rep..

[34] Mehta R.I., Schneider J.A. (2021). What is ‘Alzheimer’s disease’? The neuropathological heterogeneity of clinically defined Alzheimer’s dementia. Curr. Opin. Neurol..

[35] Mantovani E., Zucchella C., Schena F., Romanelli M.G., Venturelli M., Tamburin S. (2020). Towards a redefinition of cognitive frailty. J. Alzheimer’s Dis..

[36] Pioggiosi P., Forti P., Ravaglia G., Berardi D., Ferrari G., De Ronchi D. (2004). Different classification systems yield different dementia occurrence among nonagenarians and centenarians. Dement. Geriatr. Cogn. Discord.

[37] Chouraki V., Seshadri S. (2014). Genetics of Alzheimer’s disease. Adv. Genet..

[38] Jeong S. (2017). Molecular and cellular basis of neurodegeneration in Alzheimer’s disease. Mol. Cells.

[39] Nikolac-Perkovic M., Pivac N. (2019). Genetic markers of Alzheimer’s disease. Frontiers in Psychiatry: Artificial Intelligence, Precision Medicine, and Other Paradigm Shifts.

[40] Hakasama M.L., Vilela L.R., Marengoni A., Calderón-Larrañaga A., Leoutsakos J.-M.S., Rikkert M.G.M.O., Melis R.J.F. (2017). Comorbidity and progression of late onset alzheimer’s disease: A systematic review. PLoS ONE.

[41] Ryan J., Fransquet P., Wrigglesworth J., Lacaze P. (2018). Phenotypic heterogeneity in dementia: A challenge for epidemiology and biomarker studies. Front. Public Health.

[42] Shim H., Ly M.J., Tighe S.K. (2015). Brain imaging in the differential diagnosis of young-onset dementias. Psychiatr. Clin..

[43] Sciancalepore F. (2021). Neuroimaging and neuropsychological differences between Early-Onset alzheimer and Late-Onset alzheimer. Alzheimer’s Disease and Treatment. MedDocs Publ..

[44] Rissardo J.P., Caprara A.L. (2017). Functional and structural neuroimaging in Alzheimer’s disease: An overview. Hamdan Med. J..

[45] Cho H., Seo S.W., Kim J.H., Kim C., Ye B.S., Kim G.H., Noh Y., Kim H.J., Yoon C.W., Seong J.-K. (2013). Changes in subcortical structures in early-versus late-onset alzheimer’s disease. Neurobiol. Aging.

[46] Calvin C.M., Conroy M.C., Moore S.F., Kuźma E., Littlejohns T.J. (2022). Association of multimorbidity, disease clusters, and modification by genetic factors with risk of dementia. JAMA Netw. Open.

[47] Chen T.B., Yiao S.Y., Sun Y., Lee H.J., Yang S.C., Chiu M.J., Chen T.-F., Lin K.-N., Tang L.-Y., Lin C.-C. (2017). Comorbidity and dementia: A nationwide survey in Taiwan. PLoS ONE.

[48] Bunn F., Goodman C., Burn A.M. (2015). Multimorbidity and frailty in people with dementia. Nurs. Stand..

[49] Callahan C.M., Schubert C.C. (2014). The complexities of comorbidity in dementia. Nat. Rev. Neurol..

[50] Karlamangla A., Tinetti M., Guralnik J., Studenski S., Wetle T., Reuben D. (2007). Comorbidity in older adults: Nosology of impairment, diseases, and conditions. J. Gerontol..

[51] Feinstein A.R. (1970). The pre-therapeutic classification of co-morbidity in chronic disease. J. Chronic Dis..

[52] Valdera J.M., Starfield B., Sibbald B., Salisbury C., Roland M. (2009). Defining comorbidity: Implications for understanding health and health services. Ann. Fam. Med..

[53] Dunn R., Clayton E., Wolverson E., Hilton A. (2022). Conceptualising comorbidity and multimorbidity in dementia: A scoping review and syndemic framework. J. Multimorb. Comorbidity.

[54] Boyle P.A., Yang J., Yu L., Leurgans S.E., Capuano A.W., Schneider J.A., Wilson R.S., Bennett D.A. (2017). Varied effects of age-related neuropathologies on the trajectory of late life cognitive decline. Brain.

[55] Boyle P.A., Yu L., Wilson R.S., Leurgans S.E., Schneider J.A., Bennett D.A. (2018). Person-specific contribution of neuropathologies to cognitive loss in old age. Ann. Neurol..

[56] Boyle P.A., Wilson R.S., Yu L., Barr A.M., Honer W.G., Schneider J.A., Bennett D.A. (2013). Much of late life cognitive decline is not due to common neurodegenerative pathologies. Ann. Neurol..

[57] Boyle P.A., Yu L., Leurgans S.E., Wilson R.S., Brookmeyer R., Schneider J.A., Bennett D.A. (2019). Attributable risk of Alzheimer’s dementia attributed to age-related neuropathologies. Ann. Neurol..

[58] Vetrano D.L., Roso-Llorach A., Fernández S., Guisado-Clavero M., Violán C., Onder G., Fratiglioni L., Calderón-Larrañaga A., Marengoni A. (2020). Twelve-year clinical trajectories of multimorbidity in a population of older adults. Nat. Commun..

[59] McAleese K.E., Colloby S.J., Thomas A.J., Al-Sarraj S., Ansorge O., Neal J., Roncaroli F., Love S., Francis P.T., Attems J. (2021). Concomitant neurodegenerative pathologies contribute to the transition from mild cognitive impairment to dementia. Alzheimer’s Dement..

[60] Van Oostrom S.H., Gijsen R., Stirbu I., Korevaar J.C., Schellevis F.G., Picavet H.S.J., Hoeymans N. (2016). Time trends in prevalence of chronic diseases and multimorbidity not only due to aging: Data from general practices and health surveys. PLoS ONE.

[61] Puth M.T., Weckbecker K., Schmid M., Münster E. (2017). Prevalence of multimorbidity in Germany: Impact of age and educational level in a cross-sectional study on 19, 294 adults. BMC Public Health.

[62] Wang Y.M., Song M., Wang R., Shi L., He J., Fan T.-T., Chen W.-H., Wang L., Yu L.-L., Gao Y.-Y. (2017). Insomnia and multimorbidity in the community elderly in China. J. Clin. Sleep Med..

[63] Clague F., Mercer S.W., McLean G., Reynish E., Guthrie B. (2017). Comorbidity and polypharmacy in people with dementia: Insights from a large, population-based cross-sectional analysis of primary care data. Age Ageing.

[64] Fereshtehnjad S.M., Damangir S., Cermakova P., Airland D., Eriksdotter M., Religa D. (2014). Comorbidity profile in dementia with Lewy bodies versus Alzheimer’s disease: Linkage study between the Swedish Dementia Registry and the Swedish National Patient Registry. Alzheimer’s Res. Ther..

[65] Danielsen R., Thorgeirsson G., Einarsson H., Ólafsson Ö., Aspelund T., Harris T.B., Launer L., Gudnason V. (2017). Prevalence of heart failure in the elderly and future projections: The AGES-Reykjavik study. Scand. Cardiovasc. J..

[66] Dove A., Marseglia A., Shang Y., Grande G., Vetrano D.L., Laukka E.J., Fratiglioni L., Xu W. (2022). Cardiometabolic multimorbidity accelerates cognitive decline and dementia progression. Alzheimer’s Dement..

[67] Katabathula S., Davis P.B., Xu R. (2023). Comorbidity-driven multi-modal subtype analysis in mild cognitive impairment of Alzheimer’s disease. Alzheimer’s Dement..

[68] Bayen E., Possin K.L., Chen Y., de Langavant L.C., Yaffe K. (2018). Prevalence of aging, dementia, and multimorbidity in older adults with Down syndrome. J. Am. Med. Assoc. Neurol..

[69] Hassen C.B., Fayosse A., Landré B., Raggi M., Bloomberg M., Sabia S., Singh-Manoux A. (2022). Association between age at onset of multimorbidity and incidence of dementia: 30 year follow-up in Whitehall II prospective cohort study. BMJ.

[70] Kadmabi S., Abdallah M., Loh K.P. (2020). Multimorbidity, function, and cognition in aging. Clin. Geriatr. Med..

[71] Liu Y., Dong Y.H., Lyu P.Y., Chen W.H., Li R. (2018). Hypertension-induced cerebral small vessel disease leading to cognitive impairment. Chin. Med. J..

[72] Cannon J.A., Moffitt P., Perez-Moreno A.C., Walters M.R., Broomfield N.M., McMurray J.J., Quinn T.J. (2017). Cognitive impairment and heart failure: Systematic review and meta-analysis. J. Card. Fail..

[73] Zilliox L.A., Chadrasekaran K., Kwan J.Y., Russell J.W. (2016). Diabetes and cognitive impairment. Curr. Diabetes Rep..

[74] Doorduijn A.S., Visser M., van de Rest O., Kester M.I., de Leeuw F.A., Boesveldt S., Fieldhouse J.L.P., Heuvel E.G.H.M.v.D., Teunissen C.E., Scheltens P. (2019). Associations of AD biomarkers and cognitive performance with nutritional status: The NUDAD Project. Nutrients.

[75] Ahles T.A., Root J.C. (2018). Cognitive effects of cancer and cancer treatments. Annu. Rev. Clin. Psychol..

[76] Naismith S.L., Duffy S.L., Cross N., Grunstein R., Terpening Z., Hoyos C. (2020). Nocturnal hypoxemia is associated with altered parahippocampal functional brain connectivity in older adults at risk for dementia. J. Alzheimer’s Dis..

[77] Falvey J.R., Gustavson A.M., Price L., Papazian L., Stevens-Lapsley J.E. (2019). Dementia, comorbidity, and physical function in the program of all inclusive care for the elderly. J. Geriatr. Phys. Ther..

[78] Little M.O. (2018). Reversible dementias. Clin. Geriatr. Med..

[79] Deschaintre Y., Richard F., Leys D., Pasquier F. (2009). Treatment of vascular risk factors is associated with slower decline in Alzheimer disease. Neurology.

[80] Anderson G.F. (2010). Chronic Care: Making the Case for Ongoing Care.

[81] Centers for Medicare Medicaid Services Chronic Conditions among Medicare Beneficiaries. https://www.cms.gov/Research-Statistics-Data-and-Systems/Statistics-Trends-and-Reports/Chronic-Conditions/Downloads/2011Chartbook.pdf.

[82] Tonelli M., Wiebe N., Straus S., Fortin M., Guthrie B., James M.T., Klarenbach S.W., Tam-Tham H., Lewanczuk R., Manns B.J. (2017). Multimorbidity, dementia and health care in older people: A population-based cohort study. CMAJ Open.

[83] Barnett K., Mercer S.W., Norbury M., Watt G., Wyke S., Guthrie B. (2012). Epidemiology of multimorbidity and implications for health care, research, and medical education: A cross-sectional study. Lancet.

[84] King D.E., Xiang J., Pilkerton C.S. (2018). Multimorbidity trends in United States, Adults: 1988–2014. J. Am. Board Fam. Med..

[85] Kingston A., Robinson L., Booth H., Knapp M., Jagger C., MODEM Project (2018). Projections of multi-morbidity in the older population in England to 2035: Estimates from the population ageing and care simulation (PACSim) model. Age Aging.

[86] Skou S.T., Mair F.S., Fortin M., Guthrie B., Nunes B.P., Miranda J.J., Boyd C.M., Pati S., Mtenga S., Smith S.M. (2022). Multimorbidity. Nat. Rev. Dis. Primers.

[87] Bell K., Doust J., McGeechan K., Horvath A.R., Barratt A., Hayen A., Semsarian C., Irwig L. (2021). The potential for overdiagnosis and underdiagnosis because of blood pressure variability: A comparison of the 2017 ACC/AHA, 2018 ESC/ESH and 2019 NICE hypertension guidelines. J. Hypertens..

[88] Titlestad I., Haugarvoll K., Solvang S.E.H., Norekvål T.M., Skogseth R.E., Andreassen O.A. (2024). Delirium is frequently underdiagnosed among older hospitalised patients despite available information in hospital medical records. Age Ageing.

[89] Takeshima T., Yamamoto Y., Iwasaki K., Ha C., Oishi M., Sato A., Sonoyama Y., Honda N., Niida H., Takeda J. (2023). Prevalence, treatment status, medical costs, quality of life, and productivity loss in Japanese adult patients with anemia: A real-world database study. J. Med. Econ..

[90] Khamroev E.E., Nurboev F.E., Pulatova S.K. (2022). Chronic heart failure: Features clinical manifestations in the elderly. Br. Med. J..

[91] Lindman B.R., Patel J.N. (2016). Multimorbidity in older adults with aortic stenosis. Clin. Geriatr. Med..

[92] Ruel G., Martin S.A., Lévesque J.F., Wittert G.A., Adams R.J., Appleton S.L., Shi Z., Taylor A.W. (2018). Association between multimorbidity and undiagnosed obstructive sleep apnea severity and their impact on quality of life in men over 40 years old. Glob. Health Epidemiol. Genom..

[93] Bunn F., Burn A.M., Robinson L., Poole M., Rait G., Brayne C., Schoeman J., Norton S., Goodman C. (2017). Healthcare organisation and delivery for people with dementia and comorbidity: A qualitative study exploring the views of patients, carers and professionals. BMJ Open.

[94] Lee C.Y., Chang C.C., Lin C.S., Yeh C.C., Hu C.J., Wu C.Z., Chen T.L., Liao C.C. (2020). Risk of dementia in patients with periodontitis and related protective factors: A nationwide retrospective cohort study. J. Clin. Periodontol..

[95] Sadarangani T., Perissinotto C., Boafo J., Zhong J., Yu G. (2022). Multimorbidity patterns in adult day health center clients with dementia: A latent class analysis. BMC Geriatr..

[96] Siga O., Wizner B., Piotrowicz K., Fedyk-Łukasik M., Grodzicki T. (2017). The prevalence and determinants of multimorbidity in hospitalized patients with heart failure. Folia Med. Crac..

[97] Jafar T.H., Tan N.C., Allen J.C., Finkelstein E.A., Goh P., Moey P., Quah J.H.M., Hwang S.W., Bahadin J., Thiagarajah A.G. (2018). Management of hypertension and multiple risk factors to enhance cardiovascular health in Singapore: The Sing hypertension cluster randomized trial. Trials.

[98] Katsiki N., Anagnostis P., Kotsa K., Goulis D.G., Mikhailidis D.P. (2019). Obesity, metabolic syndrome and the risk of microvascular complications in patients with diabetes mellitus. Curr. Pharm. Des..

[99] Boudoulas K.D., Triposkiadis F., Gumina R., Addison D., Iliescu C., Boudoulas H. (2022). Cardiovascular disease, cancer, and multimorbidity interactions: Clinical implications. Cardiology.

[100] Geltser B.I., Kurpatov G., Kotelnikov V.N., Zayats Y.V. (2018). Chronic obstructive pulmonary disease and cerebrovascular disease: Functional and clinical aspect of comorbidity. Ter. Arkhiv.

[101] Widdifield J., Ivers N.M., Bernatsky S., Jaakkimainen L., Bombardier C., Thorne J.C., Ahluwalia V., Paterson J.M., Young J., Wing L. (2017). Primary care screening and comorbidity management in rheumatoid arthritis in Ontario, Canada. Arthritis Care Res..

[102] Kennedy G.J., Castro J., Chang M., Chauhan-James J., Fishman M. (2016). Psychiatric and medical comorbidity in the primary care geriatric patient: An update. Curr. Psychiatry Rep..

[103] Baré M., Herranz S., Roso-Llorach A., Jordana R., Violán C., Lleal M., Roura-Poch P., Arellano M., Estrada R., Nazco G.J. (2021). Multimorbidity patterns of chronic conditions and geriatric syndromes in older patients from the MoPIM multicentre cohort study. BMJ Open.

[104] Makizako H. (2019). Frailty and sarcopenia as a geriatric syndrome in community-dwelling older adults. Int. J. Environ. Res. Public Health.

[105] Lee P.G., Cigolle C., Blaum C. (2009). The co-occurrence of chronic diseases and geriatric syndromes: The health and retirement study. J. Am. Geriatr. Soc..

[106] Verano D.L., Palmer K., Marengoni A., Marzetti E., Lattanzio F., Roller-Wirnsberger R., Samaniego L.L., Rodríguez-Mañas L., Bernabei R., Onder G. (2019). Frailty and multimorbidity: A systematic review and meta-analysis. J. Gerontol. A Biol. Sci. Med. Sci..

[107] Ortiz P.J., Tello T., Aliaga E.G., Casas P.M., Peinado J.E., Miranda J.J., Varela L.F. (2018). Effect of multimorbidity on gait speed in well-functioning older people: A population-based study in Peru. Geriatr. Gerontol. Int..

[108] Cravello L., Di Santo S., Varrassi G., Benincasa D., Marchettini P., de Tommaso M., Shofany J., Assogna F., Perotta D., Palmer K. (2019). Chronic pain in the elderly with cognitive decline: A narrative review. Pain Ther..

[109] McKee M.M., Stransky M.L., Reichard A. (2018). Hearing loss and associated medical conditions among individuals 65 years and older. Disabil. Health J..

[110] Triolo F., Sjöberg L., Calderón-Larrañaga A., Belvederi Murri M., Vetrano D.L., Fratiglioni L., Dekhtyar S. (2023). Late-life depression and multimorbidity trajectories: The role of symptom complexity and severity. Age Ageing.

[111] Vij N., Chandramani-Shivalingappa P., Van Westphal C., Hole R., Bodas M. (2018). Cigarette smoke-induced autophagy impairment accelerates lung aging, COPD-emphysema exacerbations and pathogenesis. Am. J. Physiol. Cell. Physiol..

[112] Idalino S.C.C., Canever J.B., Cândido L.M., Wagner K.J.P., de Souza Moreira B., Danielewicz A.L., Moreira B.d.S., Danielewicz A.L., de Avelar N.C.P. (2023). Association between sleep problems and multimorbidity patterns in older adults. BMC Public Health.

[113] Nicholson K., Rodrigues R., Anderson K.K., Wilk P., Guaiana G., Stranges S. (2020). Sleep behaviours and multimorbidity occurrence in middle-aged and older adults: Findings from the Canadian Longitudinal Study on Aging (CLSA). Sleep Med..

[114] He L., Biddle S.J., Lee J.T., Duolikun N., Zhang L., Wang Z., Zhao Y. (2021). The prevalence of multimorbidity and its association with physical activity and sleep duration in middle aged and elderly adults: A longitudinal analysis from China. Int. J. Behav. Nutr. Phys. Act..

[115] Stickley A., Koyanagi A. (2018). Physical multimorbidity and loneliness: A population-based study. PLoS ONE.

[116] Stafford G., Villén N., Roso-Llorach A., Troncoso-Mariño A., Monteagudo M., Violán C. (2021). Combined multimorbidity and polypharmacy patterns in the elderly: A cross-sectional study in primary health care. Int. J. Environ. Res. Public Health.

[117] Zhang N. (2019). Comorbidity is a risk factor for poor quality of life in people with dementia. Evid.-Based Nurs..

[118] St John P.D., Tyas S.L., Menec V., Tate R., Griffith L. (2019). Multimorbidity predicts functional decline in community-dwelling older adults: Prospective cohort study. Can. Fam. Physician.

[119] Nguyen H., Manolova G., Daskalopoulou C., Vitoratou S., Prince M., Prina A.M. (2019). Prevalence of multimorbidity in community settings: A systematic review and meta-analysis of observational studies. J. Comorbidity.

[120] Kuzuya M. (2019). Era of geriatric medical challenges: Multimorbidity among older patients. Geriatr. Gerontol. Int..

[121] Agrawal S., Schneider J.A. (2022). Vascular pathology and pathogenesis of cognitive impairment and dementia in older adults. Cereb. Circ.-Cogn. Behav..

[122] Toledo J.B., Arnold S.E., Raible K., Brettschneider J., Xie S.X., Grossman M., Monsell S.E., Kukull W.A., Trojanowski J.Q. (2013). Contribution of cerebrovascular disease in autopsy confirmed neurodegenerative disease cases in the National Alzheimer’s Coordinating Centre. Brain.

[123] Yang S.K., Chen W., Su C.H., Liu C.H. (2018). Incidence and comorbidity of dementia with Lewy bodies: A population-based cohort study. Behav. Neurol..

[124] Jankovic J., Mazziotta J.C., Pomeroy S.L. (2021). Bradley’s Neurology in Clinical Practice E-Book.

[125] Nichols E., Merrick R., Hay S.I., Himali D., Himali J.J., Hunter S., Keage H.A.D., Latimer C.S., Scott M.R., Steinmetz J.D. (2023). The prevalence, correlation, and co-occurrence of neuropathology in old age: Harmonisation of 12 measures across six community-based autopsy studies of dementia. Lancet Healthy Longev..

[126] Shang X., Zhang X., Huang Y., Zhu Z., Zhang X., Liu J., Wang W., Tang S., Yu H., Ge Z. (2022). Association of a wide range of individual chronic diseases and their multimorbidity with brain volumes in the UK Biobank: A cross-sectional study. EClinicalMedicine.

[127] Beason-Held L.L., Fournier D., Shafer A.T., Fabbri E., An Y., Huang C.W., Bilgel M., Wong D.F., Ferrucci L., Resnick S.M. (2022). Disease burden affects aging brain function. J. Gerontol. Ser. A.

[128] Wang J.H., Goh J.O.S., Chang Y.L., Chen S.C., Li Y.Y., Yu Y.P., Lo R.Y. (2022). Multimorbidity and regional volumes of the default mode network in brain aging. Gerontology.

[129] Cheng Y.W., Chiu M.J., Chen Y.F., Cheng T.W., Lai Y.M., Chen T.F. (2020). The contribution of vascular risk factors in neurodegenerative disorders: From mild cognitive impairment to Alzheimer’s disease. Alzheimer’s Res. Ther..

[130] Takeda S., Rakugi H., Morishita R. (2020). Roles of vascular risk factors in the pathogenesis of dementia. Hypertens. Res..

[131] Javanshiri K., Haglund M., Englund E. (2019). Cardiovascular disease, diabetes mellitus, and hypertension in Lewy body disease: A comparison with other dementia disorders. J. Alzheimer’s Dis..

[132] Kumar M., Modi S., Rana P., Kumar P., Kanwar R., Sekhri T., D’Souza M., Khushu S. (2018). Alteration in intrinsic and extrinsic functional connectivity of resting state networks associated with subclinical hypothyroidism. J. Neuroendocrinol..

[133] Qiu C., Fratiglioni L. (2015). A major role for cardiovascular burden in age-related cognitive decline. Nat. Rev. Cardiol..

[134] Fabbri E., Zoli M., Gonzalez-Freire M., Salive M.E., Studenski S.A., Ferrucci L. (2015). Aging and multimorbidity: New tasks, priorities, and frontiers for integrated gerontological and clinical research. J. Am. Med. Dir. Assoc..

[135] Merril C.R., Zullo S., Ghanbari H., Herman M., Kleinman J., Bigelow L., Bartko J., Sabourin D. (1996). Possible relationship between conditions associated with chronic hypoxia and brain mitochondrial DNA deletions. Arch. Biochem. Biophys..

[136] Hughes M.J., McGettrick H.M., Sapey E. (2020). Shared mechanisms of multimorbidity in COPD, atherosclerosis and type-2 diabetes: The neutrophil as a potential inflammatory target. Eur. Respir. Rev..

[137] Lall R., Mohammed R., Ojha U. (2019). What are the links between hypoxia and Alzheimer’s disease?. Neuropsychiatr. Dis. Treat..

[138] Molnar L., Fülesdi B., Németh N., Molnár C. (2018). Sepsis-associated encephalopathy: A review of the literature. Neurol. India.

[139] Yan C., Zhou Y., Chen Q., Luo Y., Zhang J.H., Huang H., Shao A. (2020). Dysfunction of the neurovascular unit in diabetes-related neurodegeneration. Biomed. Pharmacother..

[140] Rehni A.K., Dave K.R. (2018). Impact of hypoglycemia on brain metabolism during diabetes. Mol. Neurobiol..

[141] Torres-Manzo A.P., Franco-Colín M., Blas-Valdivia V., Pineda-Reynoso M., Cano-Europa E. (2018). Hypothyroidism causes endoplasmic reticulum stress in adult rat hippocampus: A mechanism associated with hippocampal damage. Oxidative Med. Cell. Longev..

[142] Xu C., Zhou L., Wu K., Li Y., Xu J., Jiang D., Gao L. (2019). Abnormal glucose metabolism and insulin resistance are induced via the IRE1a/XBP-1 pathway in subclinical hypothyroidism. Front. Endocrinol..

[143] Wang X., Rostas J.A. (1996). Effect of hypothyroidism on the subcellular distribution of Ca+2/calmodulin-stimulated protein kinase II in chicken brain during posthatch development. J. Neurochem..

[144] Calderón-Larrañaga A., Vetrano D.L., Ferrucci L., Mercer S.W., Marengoni A., Onder G. (2019). Multimorbidity and functional impairment–bidirectional interplay, synergistic effects and common pathways. J. Int. Med..

[145] Brassington K., Selemidis S., Bozinovski S., Vlahos R. (2019). New frontiers in the treatment of comorbid cardiovascular disease in chronic obstructive pulmonary disease. Clin. Sci..

[146] Mueller K., Thiel F., Taskin B., Beutner F., Teren A., Dubovoy V.K., Möller H.E., Villringer A., Schroeter M.L. (2023). Brain dysconnectivity with heart failure. Brain Commun..

[147] Dridi H., Liu Y., Reiken S., Liu X., Argyrousi E.K., Yuan Q., Miotto M.C., Sittenfeld L., Meddar A., Soni R.K. (2023). Heart failure-induced cognitive dysfunction is mediated by intracellular Ca2+ leak through ryanodine receptor type 2. Nat. Neurosci..

[148] Lebouvier T., Chen Y., Duriez P., Pasquier F., Bordet R. (2020). Antihypertensive agents in Alzheimer’s disease: Beyond vascular protection. Expert Rev. Neurother..

[149] Poblador-Plou B., Calderón-Larrañaga A., Marta-Moreno J., Hancco-Saavedra J., Sicras- Mainar A., Soljak M., Prados-Torres A. (2014). Comorbidity of dementia: A cross-sectional study of primary care older patients. BMC Psychiatry.

[150] Picone P., Di Carlo M., Nuzzo D. (2020). Obesity and Alzheimer’s disease: Molecular bases. Eur. J. Neurosci..

[151] Koike M.A., Green K.N., Blurton-Jones M., LaFerla F.M. (2010). Oligemic hypoperfusion differentially affects Tau and amyloid-b. Am. J. Pathol..

[152] De Strooper B., Karran E. (2016). The cellular phase of Alzheimer’s disease. Cell.

[153] Kuang H., Zhou Z.F., Zhu Y.G., Wan Z.K., Yang M.W., Hong F.F., Yang S.L. (2021). Pharmacological treatment of vascular dementia: A molecular mechanism perspective. Aging Dis..

[154] Kovacs G.G. (2019). Are comorbidities compatible with a molecular pathological classification of neurodegenerative diseases?. Curr. Opin. Neurol..

[155] Marsh A.P. (2019). Molecular mechanisms of proteinopathies across neurodegenerative disease: A review. Neurol. Res. Pract..

[156] Tian Y.E., Cropley V., Maier A.B., Lautenschlager N.T., Breakspear M., Zalesky A. (2022). Biological aging of human body and brain systems. medRxiv.

[157] Isaev N.K., Genrikhs E.E., Oborina M.V., Stelmashook E.V. (2018). Accelerated aging and aging process in the brain. Rev. Neurosci..

[158] Damiano C., Onder G., Zazzara M.B., Carfì A., Zucchelli A., Marengoni A., Vetrano D.L. (2022). Frailty, multimorbidity patterns and mortality in institutionalized older adults in Italy. Aging Clin. Exp. Res..

[159] Nguyen Q.D., Wu C., Odden M.C., Kim D.H. (2019). Multimorbidity patterns, frailty, and survival in community-dwelling older adults. J. Gerontol. Ser. A.

[160] Hanlon P., Nicholl B.I., Jani B.D., Lee D., McQueenie R., Mair F.S. (2018). Frailty and pre-frailty in middle-aged and older adults and its association with multimorbidity and mortality: A prospective analysis of 493 737 UK Biobank participants. Lancet Public Health.

[161] Xue Q.L., Buta B., Ma L., Ge M., Carlson M. (2019). Integrating frailty and cognitive phenotypes: Why, how, now what?. Curr. Geriatr. Rep..

[162] de Morais Fabrício D., Chagas M.H.N., Diniz B.S. (2020). Frailty and cognitive decline. Transl. Res..

[163] Feder A., Fred-Torres S., Southwick S.M., Charney D.S. (2019). The biology of human resilience: Opportunities for enhancing resilience across the life span. Biol. Psychiatry.

[164] Whitson H.E., Cohen H.J., Schmader K., Morey M.C., Kuchel G., Colon-Emeric C. (2018). Physical resilience: Not simply the opposite of frailty. J. Am. Geriatr. Soc..

[165] Katzman R., Terry R., DeTeresa R., Brown T., Davies P., Fuld P., Renbing X., Peck A. (1988). Clinical, pathological, and neurochemical changes in dementia: A subgroup with preserved mental status and numerous neocortical plaques. Ann. Neurol..

[166] Bennett D.A., Wilson R.S., Boyle P.A., Buchman A.S., Schneider J.A. (2012). Relation of neuropathology to cognition in persons without cognitive impairment. Ann. Neurol..

[167] Cathomas F., Murrough J.W., Nestler E.J., Han M.H., Russo S.J. (2019). Neurobiology of resilience: Interface between mind and body. Biol. Psychiatry.

[168] Vetrano D.L., Calderón-Larrañaga A., Marengoni A., Onder G., Bauer J.M., Cesari M., Ferrucci L., Fratiglioni L. (2018). An international perspective on chronic multimorbidity: Approaching the elephant in the room. J. Gerontol. Ser. A.

[169] Aiello Bowles E.J., Crane P.K., Walker R.L., Chubak J., LaCroix A.Z., Anderson M.L., Rosenberg D., Keene C.D., Larson E.B. (2019). Cognitive resilience to Alzheimer’s disease pathology in the human brain. J. Alzheimer’s Dis..

[170] Deckers K. (2017). The Role of Lifestyle Factors in Primary Prevention of Dementia: An Epidemiological Perspective. Doctoral Thesis.

[171] Vassilaki M., Aakre J.A., Cha R.H., Kremers W.K., St. Sauver J.L., Mielke M.M., Geda Y.E., Machulda M.M., Knopman D.S., Petersen R.C. (2015). Multimorbidity and risk of mild cognitive impairment. J. Am. Geriatr. Soc..

[172] Mubashir T., Abrahamyan L., Niazi A., Piyasena D., Arif A.A., Wong J., Osorio R.S., Ryan C.M., Chung F. (2019). The prevalence of obstructive sleep apnea in mild cognitive impairment: A systematic review. BMC Neurol..

[173] Tangalos E.G., Petersen R.C. (2018). Mild cognitive impairment in geriatrics. Clin. Geriatr. Med..

[174] Malek-Ahmadi M. (2016). Reversion from mild cognitive impairment to normal cognition. Alzheimer’s Dis. Assoc. Disord..

[175] Canevelli M., Grande G., Lacorte E., Quarchioni E., Cesari M., Mariani C., Bruno G., Vanacore N. (2016). Spontaneous reversion of mild cognitive impairment to normal cognition: A systematic review of literature and meta-analysis. J. Am. Med. Dir. Assoc..

[176] Shimada H., Lee S., Makizako H. (2019). Reversible predictors of reversion from mild cognitive impairment to normal cognition: A 4-year longitudinal study. Alzheimer’s Res. Ther..

[177] Aerts L., Heffernan M., Kochan N.A., Crawford J.D., Draper B., Trollor J.N., Sachdev P.S., Brodaty H. (2017). Effects of MCI subtype and reversion on progression to dementia in a community sample. Neurology.

[178] Roberts R.O., Knopman D.S., Mielke M.M., Cha R.H., Pankratz V.S., Christianson T.J., Geda Y.E., Boeve B.F., Ivnik R.J., Tangalos E.G. (2014). Higher risk of progression to dementia in mild cognitive impairment cases who revert to normal. Neurology.

[179] Grande G., Cucumo V., Cova I., Ghiretti R., Maggiore L., Lacorte E., Galimberti D., Scarpini E., Clerici F., Pomati S. (2016). Reversible mild cognitive impairment: The role of comorbidities at baseline evaluation. J. Alzheimer’s Dis..

[180] Balogh E.P., Miller B.T., Ball J.R. (2015). Improving Diagnosis in Health Care.

[181] Croskerry P. (2009). A universal model of diagnostic reasoning. Acad. Med..

[182] Duda R.O., Hart P.E. (1973). Pattern Classification and Scene Analysis.

[183] Pi Y., Liao W., Liu M., Lu J. (2008). Theory of cognitive pattern recognition. Pattern Recognit. Tech. Technol. Appl..

[184] Ripley B.D. (2007). Pattern Recognition and Neural Networks.

[185] Volynets S., Smirnov D., Saarimäki H., Nummenmaa L. (2020). Statistical pattern recognition reveals shared neural signatures for displaying and recognizing specific facial expressions. Soc. Cogn. Affect. Neurosci..

[186] McKinnon E.T., Fridriksson J., Basilakos A., Hickok G., Hillis A.E., Spampinato M.V., Gleichgerrcht E., Rorden C., Jensen J.H., Helpern J.A. (2018). Types of naming errors in chronic post-stroke aphasia are dissociated by dual stream axonal loss. Sci. Rep..

[187] Rohrer J.D., Knight W.D., Warren J.E., Fox N.C., Rossor M.N., Warren J.D. (2008). Word-finding difficulty: A clinical analysis of the progressive aphasias. Brain.

[188] Laws K.R., Adlington R.L., Gale T.M., Moreno-Martínez F.J., Sartori G. (2007). A meta-analytic review of category naming in Alzheimer’s disease. Neuropsychologia.

[189] Vu M., Mangal R., Stead T., Lopez-Ortiz C., Ganti L. (2022). Impact of Alzheimer’s disease on caregivers in the United States. Health Psychol. Res..

[190] Sanford A.M., Morley J.E., Berg-Weger M., Lundy J., Little M.O., Leonard K., Malmstrom T.K. (2020). High prevalence of geriatric syndromes in older adults. PLoS ONE.

[191] Howard R., Schott J. (2021). When dementia is misdiagnosed. Int. J. Geriatr. Psychiatry.

[192] Langa K.M., Burke J.F. (2019). Preclinical Alzheimer disease—Early diagnosis or overdiagnosis?. JAMA Intern. Med..

[193] Cerullo E., Quinn T.J., McCleery J., Vounzoulaki E., Cooper N.J., Sutton A.J. (2021). Interrater agreement in dementia diagnosis: A systematic review and meta-analysis. Int. J. Geriatr. Psychiatry.

[194] Weinstein A.M., Gujral S., Butters M.A., Bowie C.R., Fischer C.E., Flint A.J., Herrmann N., Kennedy J.L., Mah L., Ovaysikia S. (2022). Diagnostic precision in the detection of mild cognitive impairment: A comparison of two approaches. Am. J. Geriatr. Psychiatry.

[195] Lange P.W., Lamanna M., Watson R., Maier A.B. (2019). Undiagnosed delirium is frequent and difficult to predict: Results from a prevalence survey of a tertiary hospital. J. Clin. Nurs..

[196] Wongviriyawong T., Sura-arunsumrit P., Chaiwat O., To-Adithep P., Ramlee R., Srinonprasert V. (2019). Diagnosis of postoperative delirium in older adults using the Confusion Assessment Method for the intensive care unit in non-intensive care unit settings: A test modification might improve its diagnostic performance. Geriatr. Gerontol. Int..

[197] Lanctôt K.L., Ismail Z., Bawa K.K., Cummings J.L., Husain M., Mortby M.E., Robert P. (2023). Distinguishing apathy from depression: A review differentiating the behavioral, neuroanatomic, and treatment-related aspects of apathy from depression in neurocognitive disorders. Int. J. Geriatr. Psychiatry.

[198] Inouye S.K., Studenski S., Tinetti M.E., Kuchel G.A. (2007). Geriatric syndromes: Clinical, research, and policy implications of a core geriatric concept. J. Am. Geriatr. Soc..

[199] Mirabnahrazam G., Ma D., Beaulac C., Lee S., Popuri K., Lee H. (2023). & Alzheimer’s Disease Neuroimaging Initiative. Predicting time-to-conversion for dementia of Alzheimer’s type using multi-modal deep survival analysis. Neurobiol. Aging.

[200] Zeng X.X., Zeng J.B. (2023). Systems Medicine as a Strategy to Deal with Alzheimer’s Disease. J. Alzheimer’s Dis..

[201] Seo D.O., Holtzman D.M. (2024). Current understanding of the Alzheimer’s disease-associated microbiome and therapeutic strategies. Exp. Mol. Med..

[202] Hu J.X., Thomas C.E., Brunak S. (2016). Network biology concepts in complex disease comorbidities. Nat. Rev. Genet..

[203] Barabasi A.L., Gulbahce N., Loscalzo J. (2011). Network medicine: A network-based approach to human disease. Nature.

[204] Barabasi A.L. (2007). Network medicine: From obesity to the “diseasome”. N. Eng. J. Med..

[205] Mittal K., Katare D.P. (2016). Shared links between type 2 diabetes mellitus and Alzheimer’s disease: A review. Diabetes Metab. Syndr..

[206] Al-Harazi O., Al Insaif S., Al-Ajlan M.A., Kaya N., Dzimiri N., Colak D. (2016). Integrated genomic and network-based analyses of complex diseases and human disease network. J. Genet. Genom..

[207] Hidalgo C.A., Blumm N., Barabási A.L., Christakis N.A. (2009). A dynamic network approach for the study of human phenotypes. PLoS ONE.

[208] Divo M.J., Casanova C., Marin J.M., Pinto-Plata V.M., De-Torres J.P., Zulueta J.J., Cabrera C., Zagaceta J., Sanchez-Salcedo P., Berto J. (2015). COPD comorbidities network. Eur. Respir. J..

[209] Faner R., Gutiérrez-Sacristán A., Castro-Acosta A., Grosdidier S., Gan W., Sánchez-Mayor M., Lopez-Campos J.L., Pozo-Rodriguez F., Sanz F., Mannino D. (2015). Molecular and clinical diseasome of comorbidities in exacerbated COPD patients. Eur. Respir. J..

[210] Patrinos G.P. (2023). Computational tools and repositories for precision therapeutics in the post-genomic era. 4th Belgrade Bioinformatics Conference.

[211] Ladyman J., Lambert J., Wiesner K. (2013). What is a complex system?. Eur. J. Philos. Sci..

[212] Silbersweig D., Loscalzo J. (2017). Precision psychiatry meets network medicine: Network psychiatry. JAMA Psychiatry.

[213] Rind D. (1999). Complexity and climate. Science.

[214] Seely A.J., Christou N.V. (2000). Multiple organ dysfunction syndrome: Exploring the paradigm of complex nonlinear systems. Crit. Care. Med..

[215] Cohen I.R., Harel D. (2007). Explaining a complex living system: Dynamics, multi-scaling and emergence. J. R. Soc. Interface.

[216] Kesic S. (2016). Systems biology, emergence and antireductionism. Saudi J. Biol. Sci..

[217] Bedau M. (2002). Downward causation and the autonomy of weak emergence. Principia.

[218] Sturmberg J.P. (2014). Multimorbidity and chronic disease: An emergent perspective. J. Eval. Clin. Pract..

[219] Deguet J., Demazeau Y., Magnin L. (2006). Elements about the emergence issue: A survey of emergence definition. Complexus.

[220] Fisher E.M., Wineman N.M. (2009). Conceptualizing compensatory responses: Implications for treatment and research. Biol. Res. Nurs..

[221] Hassaine A., Salimi-Khorshidi G., Canoy D., Rahimi K. (2020). Untangling the complexity of multimorbidity with machine learning. Mech. Aging Dev..

[222] Santos D., Dhamoon M.S. (2020). Trends in antihypertensive medication use among individuals with a history of stroke and hypertension: 2005 to 2016. J. Am. Med. Assoc..

[223] Verhestraeten C., Heggermont W.A., Maris M. (2020). Clinical inertia in the treatment of heart failure: A major issue to tackle. Heart Fail. Rev..

[224] Jazzar U., Shan Y., Klaassen Z., Freedland S.J., Kamat A.M., Raji M.A., Masel T., Tyler D.S., Baillargeon J., Kuo Y.-F. (2020). Impact of Alzheimer’s disease and related dementia diagnosis following treatment for bladder cancer. J. Geriatr. Oncol..

[225] Hodgson N., Gitlin L.N., Winter L., Czekanski K. (2011). Undiagnosed illness and neuropsychiatric behaviors in community residing older adults with dementia. Alzheimer Dis. Assoc. Disord..

[226] Mintzer J.E., Mirski D.F., Hoernig K.S. (2000). Behavioral and psychological signs and symptoms of dementia: A practicing psychiatrist’s viewpoint. Dialogues Clin. Neurosci..

[227] Hommel A.L.A.J., Faber M.J., Weerkamp N.J., van Dijk J.G., Bloem B.R., Koopmans R.T. (2016). Prevalence and prescribed treatments of orthostatic hypotension in institutionalized patients with Parkinson’s disease. J. Park. Dis..

[228] Kristanto A., Adiwinata R., Suminto S., Kurniawan B.N., Christianty F., Sinto R. (2016). Nocturnal hypertension: Neglected issue in comprehensive hypertension management. Acta Med. Indones..

[229] Torre C., Guerreiro J.P., Romano S., Miranda A., Longo P., Alão S., Conceição J., Laires P. (2018). Real-world prevalence of mild to moderate hypoglycemic episodes in type 2 diabetes in Portugal: Results from the HIPOS-PHARMA study. Prim. Care Diabetes.

[230] Steinman M.A., Lee S.J., John B.W., Boscardin W.J., Miao Y., Fung K.Z., Moore K.L., Schwartz J.B. (2012). Patterns of multimorbidity in elderly veterans. J. Am. Geriatr. Soc..

[231] Beck M.K., Westergaard D., Jensen A.B., Groop L., Brunak S. (2017). Temporal order of disease pairs affects subsequent disease trajectories: The case of diabetes and sleep apnea. Biocomputing.

[232] Rawtaer I., Gao Q., Nyunt M.S.Z., Feng L., Chong M.S., Lim W.S., Lee T.-S., Yap P., Yap K.B., Ng T.P. (2017). Psychosocial risk and protective factors and incident mild cognitive impairment and dementia in community dwelling elderly: Findings from the Singapore Longitudinal Aging Study. J. Alzheimer’s Dis..

[233] Steffens D.C., Maytan M., Helms M.J., Plassman B.L. (2005). Prevalence and clinical correlates of neuropsychiatric symptoms in dementia. Am. J. Alzheimer’s Dis. Other Dement..

[234] Sadavoy J. (2009). An integrated model for defining the scope of psychogeriatrics: The five Cs. Int. Psychogeriatr..

[235] Gurke R., Etyemez S., Prvulovic D., Thomas D., Fleck S.C., Reif A., Geisslinger G., Lötsch J. (2019). A data science-based analysis point at distinct patterns of lipid mediator plasma concentrations in patients with dementia. Front. Psychiatry.

[236] Langenberg C., Hingorani A.D., Whitty C.J. (2023). Biological and functional multimorbidity—From mechanisms to management. Nat. Med..

[237] Cheung J.T., Yu R., Wu Z., Wong S.Y., Woo J. (2018). Geriatric syndromes, multimorbidity, and disability overlap and increase healthcare use among older Chinese. BMC Geriatr..

[238] Ali H., Sarfraz S., Hassan L., Ali H. (2021). Atrial fibrillation as an initial presentation of apathetic thyroid storm. Cureus.

[239] Redfield M.M., Borlaug B.A. (2023). Heart failure with preserved ejection fraction: A review. JAMA.

[240] Steinberg M., Shao H., Zandi P., Lyketsos C.G., Welsh-Bohmer K.A., Norton M.C., Breitner J.C., Steffens D.C., Tschanz J.T. (2008). Point and 5-year period prevalence of neuropsychiatric symptoms in dementia: The Cache County Study. Int. J. Geriatr. Psychiatry.

[241] Fernandez-Martinez M., Castro J., Molano A., Zarranz J.J., Rodrigo R.M., Ortega R. (2008). Prevalence of neuropsychiatric symptoms in Alzheimer’s disease and vascular dementia. Curr. Alzheimer’s Res..

[242] Kverno K.S., Rabins P.V., Blass D.M., Hicks K.L., Black B.S. (2008). Prevalence and treatment of neuropsychiatric symptoms in hospice-eligible nursing home residents with advanced dementia. J. Gerontol. Nurs..

[243] Tyrrell M., Hillerås P., Skovdahl K., Fossum B., Religa D. (2016). Voices of spouses living with partners with neuropsychiatric symptoms related to dementia. Alzheimer’s Dement..

[244] Halter J.B., Ouslander J.G., Studenski S., High K.P., Asthana S., Supiano M.A., Ritchie C.S., Schmader K. (2022). Hazzard’s Geriatric Medicine and Gerontology.

[245] Jacobs J.M., Maaravi Y., Cohen A., Bursztyn M., Ein-Mor E., Stessman J. (2012). Changing profile of health and function from age 70 to 85 years. Gerontology.

[246] Castell M.V., Sánchez M., Julián R., Queipo R., Martín S., Otero Á. (2013). Frailty prevalence and slow walking speed in persons age 65 and older: Implications for primary care. BMC Fam. Pract..

[247] Afilao J. (2011). Frailty in patients with cardiovascular disease: Why, when, and how to measure. Curr. Cardiovasc. Risk Rep..

[248] Leung D.K., Chan W.C., Spector A., Wong G.H. (2021). Prevalence of depression, anxiety, and apathy symptoms across dementia stages: A systematic review and meta-analysis. Int. J. Geriatr. Psychiatry.

[249] Mullally W.J., Ronthal M., Huff K., Geschwind N. (1989). Chronic confusional state. N. J. Med..

[250] Buldyrev S.V., Parshani R., Paul G., Stanley H.E., Havlin S. (2010). Catastrophic cascade of failures in interdependent networks. Nature.

[251] Webber M.J., Tibbitt M.W. (2022). Dynamic and reconfigurable materials from reversible network interactions. Nat. Rev. Mater..

[252] Ladyman J., Wiesner K. (2020). What Is a Complex System?.

[253] Moore M.J., Demeyere N. (2019). Neglect dyslexia as a word-centered impairment: A single case study. Cortex.

[254] Consorti F., Torre D., Luzi D., Pecoraro F., Ricci F., Tamburis O. (2023). The challenge of clinical reasoning in chronic multimorbidity: Time and interactions in the Health Issues Network model. Diagnosis.

[255] Siegenfeld A.F., Bar-Yam Y. (2020). An introduction to complex systems science and its applications. Complexity.

[256] Ahmed S.S., Mohammed A.A. (2020). Effects of thyroid dysfunction on hematological parameters: Case controlled study. Ann. Med. Surg..

[257] Qiu Y., Jacobs D.M., Messer K., Salmon D.P., Feldman H. (2019). Cognitive heterogeneity in probable Alzheimer disease: Clinical and neuropathologic features. Neurology.

[258] Ferrari C., Sorbi S. (2021). The complexity of Alzheimer’s disease: An evolving puzzle. Physiol. Rev..

[259] Mukherjee S., Mez J., Trittschuh E.H., Saykin A.J., Gibbons L.E., Fardo D.W., Wessels M., Bauman J., Moore M., Choi S.-E. (2020). Genetic data and cognitively defined late-onset alzheimer’s disease subgroups. Mol. Psychiatry.

[260] Jellinger K.A. (2022). Recent update on the heterogeneity of the Alzheimer’s disease spectrum. J. Neural Transm..

[261] Habes M., Grothe M.J., Tunc B., McMillan C., Wolk D.A., Davatzikos C. (2020). Disentangling heterogeneity in Alzheimer’s disease and related dementias using data-driven methods. Biol. Psychiatry.

[262] Matsuoka T., Manabe T., Akatsu H., Hashizume Y., Yamamoto S., Ogawa N., Kanesaka T., Taniguchi C., Yamamoto T., Mizukami K. (2019). Factors influencing hospital admission among patients with autopsy-confirmed dementia. Psychogeriatrics.

[263] Fellowes S. (2022). The value of categorical Polythetic diagnoses in psychiatry. Br. J. Philos. Sci..

[264] Turkheimer F.E., Veronese M., Mondelli V., Cash D., Pariante C.M. (2023). Sickness behaviour and depression: An updated model of peripheral-central immunity interactions. Brain Behav. Immun..

[265] Polikandrioti M., Kalafatakis F., Koutelekos I., Kokoularis D. (2019). Fatigue in heart failure outpatients: Levels, associated factors, and the impact on quality of life. Arch. Med. Sci. Atheroscler. Dis..

[266] Davis J.M., Myasoedova E., Gunderson T.M., Crowson C.S. (2020). Multimorbidity and fatigue in rheumatoid arthritis: A cross-sectional study of a population-based cohort. Rheumatol. Ther..

[267] Waldman L., Morrison L.J. (2014). Sleep disorders and fatigue: Special issues in the older adult with cancer. Cancer J..

[268] Polat F., Karasu F. (2022). Effect of sleep hygiene training given to elderly individuals on daytime sleepiness and fatigue: A randomized controlled trial. Perspect. Psychiatr. Care.

[269] Le-Niculescu H., Roseberry K., Gill S.S., Levey D.F., Phalen P.L., Mullen J., Williams A., Bhairo S., Voegtline T., Davis H. (2021). Precision medicine for mood disorders: Objective assessment, risk prediction, pharmacogenomics, and repurposed drugs. Mol. Psychiatry.

[270] Zanardi R., Prestifilippo D., Fabbri C., Colombo C., Maron E., Serretti A. (2021). Precision psychiatry in clinical practice. Int. J. Psychiatry Clin. Pract..

[271] Maes M. (2022). Precision nomothetic medicine in depression research: A new depression model, and new endophenotype classes and pathway phenotypes, and a digital self. J. Pers. Med..

[272] Hasler G., Drevets W.C., Manji H.K., Charney D.S. (2004). Discovering endophenotypes for major depression. Neuropsychopharmacology.

[273] Archer D.B., Eissman J.M., Mukherjee S., Lee M.L., Choi S.E., Scollard P., Trittschuh E.H., Mez J.B., Bush W.S., Kunkle B.W. (2024). Longitudinal change in memory performance as a strong endophenotype for Alzheimer’s disease. Alzheimer’s Dement..

[274] Agrawal N., Faruqui R., Bodani M. (2020). Oxford Textbook of Neuropsychiatry.

[275] Summergrad P., Silbersweig D.A., Muskin P.R., Querques J. (2020). Textbook of Medical Psychiatry.

[276] Heilman M.K.M., Valenstein E. (2012). Clinical Neuropsychology.

[277] Teo A.R., Nelson S., Strange W., Kubo H., Katsuki R., Kurahara K., Kanba S., Kato T.A. (2020). Social withdrawal in major depressive disorder: A case-control study of hikikomori in Japan. J. Affect. Disord..

[278] Gardener H., Levin B., DeRosa J., Rundek T., Wright C.B., Elkind M.S., Sacco R.L. (2021). Social connectivity is related to mild cognitive impairment and dementia. J. Alzheimer’s Dis..

[279] Ferreira C.T., Giusiano B., Ceccaldi M., Poncet M. (1997). Optic aphasia: Evidence of the contribution of different neural systems to object and action naming. Cortex.

[280] Ferro D.A., Mutsaerts H.J., Hilal S., Kuijf H.J., Petersen E.T., Petr J., van Veluw S.J., Venketasubramanian N., Yeow T.B., Biessels G.J. (2020). Cortical microinfarcts in memory clinic patients are associated with reduced cerebral perfusion. J. Cereb. Blood Flow. Metab..

[281] Shi J., Meng R., Konakondla S., Ding Y., Duan Y., Wu D., Wang B., Luo Y., Ji X. (2017). Cerebral watershed infarcts may be induced by hemodynamic changes in blood flow. Neurol. Res..

[282] Papagno C., Pascuzzo R., Ferrante C., Casarotti A., Riva M., Antelmi L., Gennari A., Mattavelli G., Bizzi A. (2023). Deficits in naming pictures of objects are associated with glioma infiltration of the inferior longitudinal fasciculus: A study with diffusion MRI tractography, volumetric MRI, and neuropsychology. Hum. Brain Mapp..

[283] Aguirre G.K., D’Esposito M. (1999). Topographical disorientation: A synthesis and taxonomy. Brain.

[284] Hirayama K., Taguchi Y., Sato M., Tsukamoto T. (2003). Limbic encephalitis presenting with topographical disorientation and amnesia. J. Neurol. Neurosurg. Psychiatry.

[285] Gil-Neciga E., Alberca R., Boza F., Montes E., Sánchez B., Lozano R.G., García D. (2002). Transient topographical disorientation. Eur. Neurol..

[286] Vilares I., Kording K. (2011). Bayesian models: The structure of the world, uncertainty, behavior, and the brain. Ann. N. Y. Acad. Sci..

[287] Bhise V., Rajan S.S., Sittig D.F., Morgan R.O., Chaudhary P., Singh H. (2017). Defining and measuring diagnostic uncertainty in medicine: A systematic review. J. Gen. Intern. Med..

[288] Tonelli M.R., Upshur R.E. (2019). A philosophical approach to addressing uncertainty in medical education. Acad. Med..

[289] Poiliti M.C., Han P.K., Col N.F. (2007). Communicating the uncertainty of harms and benefits of medical interventions. Med. Decis. Mak..

[290] Osler W. (1961). Aphorisms from His Bedside Teaching and Writings.

[291] Kim K., Lee Y.M. (2018). Understanding uncertainty in medicine: Concepts and implications in medical education. Korean J. Med. Educ..

[292] Dhawale T., Steuten L.M., Deeg H.J. (2017). Uncertainty of physicians and patients in med decision making. Biol. Blood Marrow Transpl..

[293] Evans J.S.B.T., Stanovich K.E. (2013). Dual-process theories of higher cognition: Advancing the debate. Perspect. Psychol. Sci..

[294] Evans J.S.B.T. (2008). Dual-processing accounts of reasoning, judgment, and social cognition. Annu. Rev. Psychol..

[295] Kahneman D. (2011). Thinking, Fast and Slow.

[296] Cahill S., Clark M., O’Connell H., Lawlor B., Coen R.F., Walsh C. (2008). The attitudes and practices of general practitioners regarding dementia diagnosis in Ireland. Int. J. Geriatr. Psychiatry.

[297] Skinner T.R., Scott I.A., Martin J.H. (2016). Diagnostic errors in older patients: A systematic review of incidence and potential causes in seven prevalent diseases. Int. J. Gen. Med..

[298] Abimanyi-Ochom J., Bohingamu Mudiyanselage S., Catchpool M., Firipis M., Wanni Arachchige Dona S., Watts J.J. (2019). Strategies to reduce diagnostic errors: A systematic review. BMC Med. Inform. Decis. Mak..

[299] Chen X., Liu X., Parker B.J., Zhen Z., Weiner K.S. (2023). Functionally and structurally distinct fusiform face area (s) in over 1000 participants. NeuroImage.

[300] Thammasitboon S., Cutrer W.B. (2013). Diagnostic decision-making and strategies to improve diagnosis. Curr. Probl. Pediatr. Adolesc. Health Care.

[301] Wangler J., Jansky M. (2020). Dementia diagnostics in general practitioner care: Do general practitioners have reservations? The findings of a qualitative study in Germany. Wien. Med. Wochenschr..

[302] Klein J.G. (2005). Five pitfalls in decisions about diagnosis and prescribing. BMJ.

[303] Kahneman D., Tversky A. (1973). On the psychology of prediction. Psychol. Rev..

[304] Croskerry P., Singhal G., Mamede S. (2012). Cognitive debiasing 1: Origins of bias and theory of debiasing. BMJ Qual. Saf..

[305] Phung T.K.T., Andersen B.B., Kessing L.V., Mortensen P.B., Waldemar G. (2009). Diagnostic evaluation of dementia in the secondary health care sector. Dement. Geriatr. Cogn. Disord..

[306] Drachman D.A. (2000). Occam’s razor, geriatric syndromes, and the dizzy patient. Ann. Intern. Med..

[307] Jeffreys W.H., Berger J.O. (1992). Ockham’s razor and bayesian analysis. Am. Sci..

[308] Clark A. (2013). Whatever next? Predictive brains, situated agents, and the future of cognitive science. Behav. Brain Sci..

[309] Croskerry P., Singhal G., Mamede S. (2012). Cognitive debiasing 2: Impediments to and strategies for change. BMJ Qual. Saf..

[310] Croskerry P. (2003). The importance of cognitive errors in diagnosis and strategies to minimize them. Acad. Med..

[311] Croskerry P., Abbass A.A., Wu A.W. (2008). Doctors feel: Affective issues in patients’ safety. Lancet.

[312] Bastin C., Besson G., Simon J., Delhaye E., Geurten M., Willems S., Salmon E. (2019). An integrative memory model of recollection and familiarity to understand memory deficits. Behav. Brain Sci..

[313] Mock N., Balzer C., Gutbrod K., De Haan B., Jäncke L., Ettlin T., Trost W. (2022). Lesion-symptom mapping corroborates lateralization of verbal and nonverbal memory processes and identifies distributed brain networks responsible for memory dysfunction. Cortex.

[314] Marselli G., Favieri F., Casagrande M. (2023). Episodic and Semantic Autobiographical Memory in Mild Cognitive Impairment (MCI): A Systematic Review. J. Clin. Med..

[315] Irish M. (2023). Autobiographical memory in dementia syndromes—An integrative review. Wiley Interdiscip. Rev. Cogn. Sci..

[316] Brewin C.R. (2018). Memory and forgetting. Curr. Psychiatry Rep..

[317] Browning C.A., Thompson C.L., Kochan N.A., Brodaty H., Sachdev P.S., Henry J.D. (2023). Prospective memory function predicts future cognitive decline and incident dementia. J. Gerontol. Ser. B.

[318] Prasad S., Katta M.R., Abhishek S., Sridhar R., Valisekka S.S., Hameed M., Kaur J., Walia N. (2023). Recent advances in Lewy body dementia: A comprehensive review. Disease-a-Month.

[319] Sharps M.J., Hess A.B., Ranes B. (2007). Mindless decision making and environmental issues: Gestalt/feature-intensive processing and contextual reasoning in environmental decisions. J. Psychol..

[320] Mullane K., Williams M. (2019). The de-Alzheimerization of age-related dementias: Implications for drug targets and approaches to effective therapeutics. Curr. Opin. Pharmacol..

[321] Royall D. (2003). The “alzheimerization” of dementia research. J. Am. Geriatr. Soc..

[322] Banks W.A. (2020). The blood-brain barrier interface in diabetes mellitus: Dysfunctions, mechanisms and approaches to treatment. Curr. Pharm. Des..

[323] Raz L., Bhaskar K., Weaver J., Marini S., Zhang Q., Thompson J.F., Espinoza C., Iqbal S., Maphis N.M., Weston L. (2019). Hypoxia promotes tau hyperphosphorylation with associated neuropathology in vascular dysfunction. Neurobiol. Dis..

[324] Halder S.K., Milner R. (2020). Mild hypoxia triggers transient blood–brain barrier disruption: A fundamental protective role for microglia. Acta Neuropathol. Commun..

[325] Li H., Xia N. (2020). The role of oxidative stress in cardiovascular disease caused by social isolation and loneliness. Redox Biol..

[326] Divo M., Celli B.R. (2020). Multimorbidity in patients with chronic obstructive pulmonary disease. Clin. Chest Med..

[327] Fan J., Yang J., Jiang Z. (2018). Prediction of central nervous system side effects through drug permeability to blood-brain barrier and recommendation algorithm. J. Comput. Biol..

[328] Sweeney M.D., Zhao Z., Montagne A., Nelson A.R., Zlokovic B.V. (2019). Blood-brain barrier: From physiology to disease and back. Physiol. Rev..

[329] Noe C.R., Noe-Letschnig M., Handschuh P., Noe C.A., Lanzenberger R. (2020). Dysfunction of the blood-brain barrier—A key step in neurodegeneration and dementia. Front. Aging Neurosci..

[330] Galea I. (2021). The blood–brain barrier in systemic infection and inflammation. Cell. Mol. Immunol..

[331] Sun W., Zhuo S., Wu H., Cai X. (2023). Association between Coronary Heart Disease, Heart Failure, and Risk of Alzheimer’s Disease: A Systematic Review and Meta-Analysis. Ann. Indian Acad. Neurol..

[332] Verhulst C.E., Fabricius T.W., Nefs G., Kessels R.P., Pouwer F., Teerenstra S., Tack C.J., Broadley M.M., Kristensen P.L., McCrimmon R.J. (2022). Consistent effects of hypoglycemia on cognitive function in people with or without diabetes. Diabetes Care.

[333] Liu L., Eisen H.J. (2014). Epidemiology of heart failure and scope of the problem. Cardiol. Clin..

[334] Laroche C.M., Cairns T., Moxham J., Green M. (1988). Hypothyroidism presenting with respiratory muscle weakness. Am. Rev. Respir. Dis..

[335] Muthulakshmi N., Mala S.L. (2021). A study of prevalence of anemia among hypothyroid women during pregnancy. Int. Arch. Integr. Med..

[336] Farmakis D., Chrysohoou C., Giamouzis G., Giannakoulas G., Hamilos M., Naka K., Tzeis S., Xydonas S., Karavidas A., Parissis J. (2020). The management of atrial fibrillation in heart failure: An expert panel consensus. Heart Fail. Rev..

[337] Enright P.L., McClelland R.L., Newman A.B., Gottlieb D.J., Lebowitz M.D., for the Cardiovascular Health Study Research Group (1999). Underdiagnosis and undertreatment of asthma in the elderly. Chest.

[338] Dombrowsky A., Borg B., Xie R., Kirklin J.K., Chen H., Balentine C.J. (2018). Why is hyperparathyroidism underdiagnosed and undertreated in older adults?. Clin. Med. Insights Endocrinol. Diabetes.

[339] Frost M., Wraae K., Gudex C., Nielsen T., Brixen K., Hagen C., Andersen M. (2012). Chronic disease in elderly men: Underreporting and underdiagnosis. Age Ageing.

[340] Jellinger K.A., Attems J. (2015). Challenges of multimorbidity of the aging brain: A critical update. J. Neural Transm..

[341] Nelson P.T., Head E., Schmitt F.A., Davis P.R., Neltner J.H., Jicha G.A., Abner E.L., Smith C.D., Van Eldik L.J., Kryscio R.J. (2011). Alzheimer’s disease is not “brain aging”: Neuropathological, genetic, and epidemiological human studies. Acta Neuropathol..

[342] Haug N., Sorger J., Gisinger T., Gyimesi M., Kautzky-Willer A., Thurner S., Klimek P. (2021). Decompression of multimorbidity along the disease trajectories of diabetes mellitus patients. Front. Physiol..

[343] González R., Rojas M., Rosselli M., Ardila A. (2022). Linguistic profiles of variants of primary progressive aphasia. J. Commun. Disord..

[344] Patel N., Peterson K.A., Ingram R.U., Storey I., Cappa S.F., Catricala E., Halai A., Patterson K.E., Lambon Ralph M.A., Rowe J.B. (2022). A ‘Mini Linguistic Examination’to classify primary progressive aphasia. Brain Commun..

[345] Henskens L.H., Kroon A.A., van Oostenbrugge R.J., Gronenschild E.H., Hofman P.A., Lodder J., de Leeuw P.W. (2009). Associations of ambulatory blood pressure levels with white matter hyperintensity volumes in hypertensive patients. Am. J. Hypertens..

[346] Kim B., Reif E., Wattenberg M., Bengio S., Mozer M.C. (2021). Neural networks trained on natural scenes exhibit gestalt closure. Comput. Brain Behav..

[347] Gundlach H. (2020). Gestalt Psychology. Oxford Research Encyclopedia of Psychology.

[348] Van Kleeck M.H., Kosslyn S.M. (1989). Gestalt laws of perceptual organization in an embedded figures task: Evidence for hemispheric specialization. Neuropsychologia.

[349] Sabary S., Devyatko D., Kimchi R. (2020). The role of visual awareness in processing of global structure: Evidence from the perceptual organization of hierarchical patterns. Cognition.

[350] Tononi G., Edelman G.M., Sporns O. (1998). Complexity and coherency: Integrating information in the brain. Trends Cogn. Sci..

[351] Westlin C., Theriault J.E., Katsumi Y., Nieto-Castanon A., Kucyi A., Ruf S.F., Brown S.M., Pavel M., Erdogmus D., Brooks D.H. (2023). Improving the study of brain-behavior relationships by revisiting basic assumptions. Trends Cogn. Sci..

[352] Jørgensen I.F., Aguayo-Orozco A., Lademann M., Brunak S. (2020). Age-stratified longitudinal study of Alzheimer’s and vascular dementia patients. Alzheimer’s Dement..

[353] Aksman L.M., Lythgoe D.J., Williams S.C., Jokisch M., Mönninghoff C., Streffer J., Jöckel K., Weimar C., Marquand A.F. (2016). Making use of longitudinal information in pattern recognition. Hum. Brain Mapp..

[354] Bhagwat N., Viviano J.D., Voineskos A.N., Chakravarty M.M. (2018). Alzheimer’s disease neuroimaging initiative: Modeling and prediction of clinical symptom trajectories in Alzheimer’s disease using longitudinal data. PLoS ONE Comput. Biol..

[355] Fujisawa K., Tsunoda S., Hino H., Shibuya K., Takeda A., Aoki N. (2015). Alzheimer’s disease or Alzheimer’s syndrome? A longitudinal computed tomography neuroradiological follow-up study of 56 cases diagnosed clinically as Alzheimer’s disease. Psychogeriatrics.

[356] Robertson A.D., Messner M.A., Shirzadi Z., Kleiner-Fisman G., Lee J., Hopyan J., Lang A.E., Black S.E., MacIntosh B.J., Masellis M. (2016). Orthostatic hypotension, cerebral hypoperfusion, and visuospatial deficits in Lewy body disorders. Park. Relat. Disord..

[357] Koga S., Roemer S.F., Kasanuki K., Dickson D.W. (2019). Cerebrovascular pathology presenting as corticobasal syndrome: An autopsy case series of “Vascular CBS”. Park. Relat. Disord..

[358] Göthlin M., Eckerström M., Lindwall M., Rolstad S., Eckerström C., Jonsson M., Kettunen P., Svensson J., Wallin A. (2020). Latent cognitive profiles differ between incipient Alzheimer’s disease and dementia with subcortical vascular lesions in a memory clinic population. J. Alzheimer’s Dis..

[359] Wang D.Q., Wang L., Wei M.M., Xia X.S., Tian X.L., Cui X., Li X. (2020). Relationship between type 2 diabetes and white matter hyperintensity: A systematic review. Front. Endocrinol..

[360] Conen D., Rodondi N., Müller A., Beer J.H., Ammann P., Moschovitis G., Auricchio A., Hayoz D., Kobza R., Shah D. (2019). Relationships of overt and silent brain lesions with cognitive function in patients with atrial fibrillation. J. Am. Coll. Cardiol..

[361] Garrett M.F., Caplan D., Lecours A.R., Smith A. (1984). The organization of processing structure for language production: Applications to aphasic speech. Biological Perspectives of Language.

[362] Macoir J., Routhier S., Auclair-Ouellet N., Wilson M.A., Hudon C. (2023). Validation of and normative data of the DVAQ-30, a new video-naming test for assessing verb anomia. Arch. Clin. Neuropsychol..

[363] Writing Committee (2023). Birtcher, K.K.; Allen, L.A.; Anderson, J.L.; Bonaca, M.P.; Gluckman, T.J.; Hussain, A.; Kosiborod, M.; Mehta, L.S.; Virani, S.S. 2022 ACC expert consensus decision pathway for integrating atherosclerotic cardiovascular disease and multimorbidity treatment: A framework for pragmatic, patient-centered care: A report of the American College of Cardiology Solution Set Oversight Committee. J. Am. Coll. MCardiology.

[364] Katayama O., Lee S., Bae S., Makino K., Shinkai Y., Chiba I., Harada K., Shimada H. (2020). Modifiable risk factor possession patterns of dementia in elderly with MCI: A 4-year repeated measures study. J. Clin. Med..

[365] James B.D., Bennett D.A. (2019). Causes and patterns of dementia: An update in the era of redefining Alzheimer’s disease. Annu. Rev. Public Health.

[366] Solomon J.G. (1979). Remediable causes of dementia. Va. Med..

[367] Weytingh M.D., Bossuyt P.M.M., Van Crevel H. (1995). Reversible dementia: More than 10% or less than 1%? A quantitative review. J. Neurol..

[368] Task Force Sponsored by the National Institute on Aging (1980). Senility reconsidered: Treatment possibilities for mental impairment in the elderly. J. Am. Med. Assoc..

[369] Mahler M.E., Cummings J.L., Benson D.F. (1987). Treatable dementias. West. J. Med..

[370] Chen M., Maleski J.J., Sawmiller D.R. (2011). Scientific truth or false hope? Understanding Alzheimer’s disease from an aging perspective. J. Alzheimer’s Dis..

[371] Hillis A.E., Gold L., Kannan V., Cloutman L., Kleinman J.T., Newhart M., Heidler-Gary J., Davis C., Aldrich E., Llinas R. (2008). Site of the ischemic penumbra as a predictor of potential for recovery of functions. Neurology.

[372] Dubois B., Feldman H.H., Jacova C., Hampel H., Molinuevo J.L., Blennow K., DeKosky S.T., Gauthier S., Selkoe D., Bateman R. (2014). Advancing research diagnostic criteria for Alzheimer’s disease: The IWG-2 criteria. Lancet Neurol..

[373] Thietart R.A., Forgues B. (1995). Chaos theory and organization. Organ. Sci..

[374] Jesmi A.A., Jouybari L.M., Sanagoo A. (2020). Application of chaos theory in the patient’s safety. J. Nurs. Midwifery Sci..

[375] Vogt P.K., Hart J.R., Yates J.R. (2016). A butterfly effect in cancer. Mol. Cell. Oncol..

[376] Hiver P., Al-Hoorie A.H., Evans R. (2022). Complex dynamic systems theory in language learning: A scoping review of 25 years of research. Stud. Second Lang. Acquis..

[377] Chen G., Danca M.F., Xiaosong Y., Martinez G.J., Yu H. (2018). Research frontier in chaos theory and complex networks. Entropy.

[378] Clatici V.G., Satolli F., Alin-Laurentiu T.A.T.U., Voicu C., Draganita A.M.V., Lotti T. (2018). Butterfly effect: The concept and the implications in dermatology, acne and rosacea. Maedica.

[379] Chau C.T., Prielipp R.C., Wahr J.A. (2018). prevention of thrombophlebitis in peripheral intravenous catheters: The butterfly effect. Anesth. Analg..

[380] Van Leijsen E.M., de Leeuw F.E., Tuladhar A.M. (2017). Disease progression and regression in sporadic small vessel disease: Insights from neuroimaging. Clin. Sci..

[381] Uiterwijk R., Staals J., Huijts M., de Leeuw P.W., Kroon A.A., van Oostenbrugge R.J. (2017). MRI progression of cerebral small vessel disease and cognitive decline in patients with hypertension. Am. J. Hypertens..

[382] Firbank M.J., Wiseman R.M., Burton E.J., Saxby B.K., O’Brien J.T., Ford G.A. (2007). Brain atrophy and white matter hyperintensity change in older adults and relationship to blood pressure. J. Neurol..

[383] Lehtisalo J., Palmer K., Mangialasche F., Solomon A., Kivipelto M., Ngandu T. (2021). Changes in lifestyle, behaviors, and risk factors for cognitive impairment in older persons during the first wave of the coronavirus disease 2019 pandemic in Finland: Results from the FINGER study. Front. Psychiatry.

[384] Balconi M., Angioletti L. (2021). Neuroenhancement of the Executive Functions in Addiction. Advances in Substance and Behavioral Addiction: The Role of Executive Functions.

[385] Tobin M.K., Musaraca K., Disouky A., Shetti A., Bheri A., Honer W.G., Kim N., Dawe R.J., Bennett D.A., Arfanakis K. (2019). Human hippocampal neurogenesis persists in aged adults and Alzheimer’s disease patients. Cell Stem Cell.

[386] Koydemir S., Sökmez A.B., Schütz A. (2021). A meta-analysis of the effectiveness of randomized controlled positive psychological interventions on subjective and psychological well-being. Appl. Res. Qual. Life.

[387] Wu T., Lin D., Cheng Y., Jiang S., Riaz M.W., Fu N., Mou C., Ye M., Zheng Y. (2022). Amyloid cascade hypothesis for the treatment of alzheimer’s disease: Progress and challenges. Aging Dis..

[388] Takeda S. (2019). Tau propagation as a diagnostic and therapeutic target for dementia: Potentials and unanswered questions. Front. Neurosci..

[389] Motoi Y., Hanger D.P., Hasegawa M. (2020). Tau Propagation Mechanisms: Cell Models, Animal Models, and Beyond. Front. Neurosci..

[390] Walton C.C., Begelman D., Nguyen W., Andersen J.K. (2020). Senescence as an amyloid cascade: The amyloid senescence hypothesis. Front. Neurosci..

[391] Chouliaras L., Kumar G.S., Thomas A.J., Lunnon K., Chinnery P.F., O’Brien J.T. (2020). Epigenetic regulation in the pathophysiology of Lewy body dementia. Prog. Neurobiol..

[392] Calabresi P., Di Lazzaro G., Marino G., Campanelli F., Ghiglieri V. (2023). Advances in understanding the function of alpha-synuclein: Implications for Parkinson’s disease. Brain.

[393] Cantarero-Prieto D., Leon P.L., Blazquez-Fernandez C., Juan P.S., Cobo C.S. (2020). The economic cost of dementia: A systematic review. Dementia.

[394] Soley-Bori M., Ashworth M., Bisquera A., Dodhia H., Lynch R., Wang Y., Fox-Rushby J. (2021). Impact of multimorbidity on healthcare costs and utilisation: A systematic review of the UK literature. Br. J. Gen. Pract..

[395] Piepoli M.F., Abreu A., Albus C., Ambrosetti M., Brotons C., Catapano A.L., Corra U., Cosyns B., Deaton C., Graham I. (2020). Update on cardiovascular prevention in clinical practice: A position paper of the European Association of Preventive Cardiology of the European Society of Cardiology. Eur. J. Prev. Cardiol..

[396] Hughes J.C., Ingram T.A., Jarvis A., Denton E., Lampshire Z., Wernham C. (2017). Consent for the diagnosis of preclinical dementia states: A review. Maturitas.

[397] Naue U. (2008). ‘Self-care without a self’: Alzheimer’s disease and the concept of personal responsibility for health. Med. Health Care Philos..

[398] Yu J.T., Xu W., Tan C.C., Andrieu S., Suckling J., Evangelou E., Pan A., Zhang C., Jia J., Feng L. (2020). Evidence-based prevention of Alzheimer’s disease: Systematic review and meta-analysis of 243 observational prospective studies and 153 randomised controlled trials. J. Neurol. Neurosurg. Psychiatry.

[399] Saito T., Murata C., Saito M., Takeda T., Kondo K. (2018). Influence of social relationship domains and their combinations on incident dementia: A prospective cohort study. J. Epidemiol. Community. Health.

[400] Van Meijel L.A., De Vegt F., Abbink E.J., Rutters F., Schram M.T., Van Der Klauw M.M., Wolffenbuttel B.H., Siegelaar S., DeVries J.H., Sijbrands E.J. (2020). High prevalence of impaired awareness of hypoglycemia and severe hypoglycemia among people with insulin-treated type 2 diabetes: The Dutch Diabetes Pearl Cohort. BMJ Open Diabetes Res. Care.

[401] Scheen A.J. (2021). Careful use to minimize adverse events of oral antidiabetic medications in the elderly. Expert Opin. Pharmacother..

[402] Shariff A., Sridhar S.B., Bittar H.R., Hamad A., Ahmed R., Kadour G. (2019). Frequency and predisposing factors for drug-induced hypoglycemia in patients with type-2 diabetes mellitus. J. Res. Pharm. Pract..

[403] Patou F., Ciccone N., Thorpe J., Maier A. (2020). Designing P4 healthcare interventions for managing cognitive decline and dementia: Where are we at?. J. Eng. Des..

[404] Dintica C.S., Bahorik A., Xia F., Kind A., Yaffe K. (2023). Dementia risk and disadvantaged neighborhoods. JAMA Neurol..

[405] McKhann G.M., Knopman D.S., Chertkow H., Hyman B.T., Jack C.R., Kawas C.H., Klunk W.E., Koroshetz W.J., Manly J.J., Mayeux R. (2011). The diagnosis of dementia due to Alzheimer’s disease: Recommendations from the National Institute on Aging-Alzheimer’s Association workgroups on diagnostic guidelines for Alzheimer’s disease. Alzheimer’s Dement..

[406] McKhann G., Drachman D., Folstein M., Katzman R., Price D., Stadlan E.M. (1984). Clinical diagnosis of Alzheimer’s disease: Report of the NINCDS-A D.R.DA Work Group under the auspices of Department of Health and Human Services Task Force on Alzheimer’s Disease. Neurology.

[407] American Psychiatric Association (2000). Diagnostic and Statistical Manual of Mental Disorders.

[408] Dubois B., Feldman H.H., Jacova C., DeKosky S.T., Barberger-Gateau P., Cummings J., Delacourte A., Galasko D., Gauthier S., Jicha G. (2007). Research criteria for the diagnosis of AD: Revising the NINCDS-A D.R.D.A. criteria. Lancet Neurol..

[409] Duara R., Barker W. (2023). Heterogeneity in Alzheimer’s disease diagnosis and progression rates: Implications for therapeutic trials. Neurotherapeutics.

[410] Devi G., Scheltens P. (2018). Heterogeneity of Alzheimer’s disease: Consequence for drug trials?. Alzheimer’s Res. Ther..

[411] Amidei C.B., Fayosse A., Dumurgier J., Machado-Fragua M.D., Tabak A.G., van Sloten T., Kivimäki M., Dugravot A., Sabia S., Singh-Manoux A. (2021). Association between age at diabetes onset and subsequent risk of dementia. JAMA.

[412] Yang P.S., Sung J.H., Jang E., Yu H.T., Kim T.-H., Uhm J.-S., Kim J.-Y., Pak H.-N., Lee M.-H., Lip G.Y.H. (2020). The effect of integrated care management on dementia in atrial fibrillation. J. Clin. Med..

[413] Pierce A.L., Kawas C.H. (2017). Dementia in the oldest old: Beyond Alzheimer disease. PLoS Med..

[414] He J., Morales D.R., Guthrie B. (2020). Exclusion rates in randomized controlled trials of treatments for physical conditions: A systematic review. Trials.

[415] Van Dyck C.H., Swanson C.J., Aisén P., Bateman R.J., Chen C., Gee M., Kanekiyo M., Li D., Reyderman L., Cohen S. (2023). Lucania in early Alzheimer’s disease. N. Engl. J. Med..

[416] Tiwari V., Shukla S. (2023). Lipidomics and proteomics: An integrative approach for early diagnosis of dementia and Alzheimer’s disease. Front. Genet..

[417] Yu L., Petyuk V.A., Gaiteri C., Mostafavi S., Young-Pearse T., Shah R.C., Buchman A.S., Schneider J.A., Piehowski P.D., Sontag R.L. (2018). Targeted brain proteomics uncover multiple pathways to Alzheimer’s dementia. Ann. Neurol..

[418] Hosoki S., Hansra G.K., Jayasena T., Poljak A., Mather K.A., Catts V.S., Rust R., Sagare A., Kovacic J.C., Brodtmann A. (2023). Molecular biomarkers for vascular cognitive impairment and dementia. Nat. Rev. Neurol..

[419] Beven-Jones W.R., Cope T.E., Jones P.S., Kaalund S.S., Passamonti L., Allinson K., Green O., Hong Y.T., Fryer T.D., Arnold R. (2020). Neuroinflammation and protein aggregation colocalize across the frontotemporal dementia spectrum. Brain.

[420] Bousiges O., Blanc F. (2022). Biomarkers of dementia with Lewy bodies: Differential diagnostic with Alzheimer’s disease. Int. J. Mol. Sci..

[421] Sweeney M.D., Montagne A., Sagare A.P., Nation D.A., Schneider L.S., Chui H.C., Harrington M.G., Pa J., Law M., Wang D.J.J. (2019). Vascular dysfunction: The disregarded partner of Alzheimer’s disease. Alzheimer’s Dement..

[422] Skrobot O.A., Black S.E., Chen C., DeCarli C., Erkinjuntti T., Ford G.A., Kalaria R.N., O’Brien J., Pantoni L., Pasquier F. (2018). Progress toward standardized diagnosis of vascular cognitive impairment: Guidelines from the Vascular Impairment of Cognition Classification Consensus Study. Alzheimer’s Dement..

[423] Uchida Y., Kan H., Sakurai K., Oishi K., Matsukawa N. (2023). Contributions of blood–brain barrier imaging to neurovascular unit pathophysiology of Alzheimer’s disease and related dementias. Front. Aging Neurosci..

[424] Reitz C., Pericak-Vance M.A., Foroud T., Mayeux R. (2023). A global view of the genetic basis of Alzheimer disease. Nat. Rev. Neurol..

[425] Andrews S.J., Renton A.E., Fulton-Howard B., Podlesny-Drabiniok A., Marcora E., Goate A.M. (2023). The complex genetic architecture of Alzheimer’s disease: Novel insights and future directions. EBioMedicine.

[426] Martin-Segura A., Benvegnù S. (2020). Molecular alteration contributing to brain aging. J. Neurosci. Res..

[427] Guo J., Huang X., Dou L., Yan M., Shen T., Tang W., Li J. (2022). Aging and aging-related diseases: From molecular mechanisms to interventions and treatments. Signal Transduct. Target. Ther..

[428] Aman Y., Schmauck-Medina T., Hansen M., Morimoto R.I., Simon A.K., Bjedov I., Palikaras K., Simonsen A., Johansen T., Tavernarakis N. (2021). Autophagy in healthy aging and disease. Nat. Aging.

[429] Nehls M. (2016). Unified theory of Alzheimer’s disease (UTAD): Implications for prevention and curative therapy. J. Mol. Psychiatry.

[430] Grande G., Qiu C., Fratiglioni L. (2020). Prevention of dementia in an ageing world: Evidence and biological rationale. Ageing Res. Rev..

[431] Li H., Li S., Yang H., Zhang Y., Zhang S., Ma Y., Hou Y., Zhang X., Niu K., Borné Y. (2022). Association of ultraprocessed food consumption with risk of dementia: A prospective cohort study. Neurology.

[432] Veronese N., Soysal P., Demurtas J., Solmi M., Bruyère O., Christodoulou N., Ramalho R., Fusar-Poli P., Lappas A.S., Pinto D. (2023). Physical activity and exercise for the prevention and management of mild cognitive impairment and dementia: A collaborative international guideline. Eur. Geriatr. Med..

[433] Stuart K.E., Padgett C. (2020). A systematic review of the association between psychological stress and dementia risk in humans. J. Alzheimer’s Dis..

[434] Schulkin J., Sterling P. (2019). Allostasis: A brain-centered, predictive mode of physiological regulation. Trends Neurosci..

[435] Frere S., Slutsky I. (2018). Alzheimer’s disease: From firing instability to homeostasis network collapse. Neuron.

[436] Formiga F., Fort I., Robles M.J., Riu S., Sabartes O., Barranco E., Catena J. (2009). Comorbidity and clinical features in elderly patients with dementia: Differences according to dementia severity. J. Nutr. Health Aging.

[437] Peters R., Booth A., Rockwood K., Peters J., D’Este C., Anstey K.J. (2019). Combining modifiable risk factors and risk of dementia: A systematic review and meta-analysis. BMJ Open.

[438] Doraiswamy P.M., Leon J., Cummings J.L., Marin D., Neumann P.J. (2002). Prevalence and impact of medical comorbidity in Alzheimer’s disease. J. Gerontol..

[439] Fabbrie E., An Y., Zoli M., Tanaka T., Simonsick E.M., Kitner-Triolo M.H., Studenski S.A., Resnick S.M., Ferrucci L. (2016). Association between accelerated multimorbidity and age-related cognitive decline in older Baltimore: Longitudinal study of ageing participants without dementia. J. Am. Geriatr. Soc..

[440] Tai X.Y., Veldsman M., Lyall D.M., Littlejohns T.J., Langa K.M., Husain M., Ranson J., Llewellyn D.J. (2022). Cardiometabolic multimorbidity, genetic risk, and dementia: A prospective cohort study. Lancet Healthy Longev..

[441] Solis Jr E., Hascup K.N., Hascup E.R. (2020). Alzheimer’s disease: The link between amyloid-β and neurovascular dysfunction. J. Alzheimer’s Dis..

[442] Azam S., Haque M.E., Balakrishnan R., Kim I.S., Choi D.K. (2021). The ageing brain: Molecular and cellular basis of neurodegeneration. Front. Cell Dev. Biol..

[443] Lloret A., Monllor P., Esteve D., Cervera-Ferri A., Lloret A. (2019). Obesity as a risk factor for Alzheimer’s disease: Implication of leptin and glutamate. Front. Neurosci..

[444] Kent S.A., Spires-Jones T.L., Durrant C.S. (2020). The physiological roles of tau and Ab: Implications for Alzheimer’s disease pathology and therapeutics. Acta Neuropathol..

[445] Iadanza M.G., Jackson M.P., Hewitt E.W., Ranson N.A., Radford S.E. (2018). A new era for understanding amyloid structures and disease. Nat. Rev..

[446] Cairns B.J., Balkwill A., Canoy D., Green J., Reeves G.K., Beral V. (2015). Variations in vascular mortality trends, 2001–2010, among 1.3 million women with different lifestyle risk factors for the disease. Eur. J. Prev. Cardiol.

[447] Chen H., Zhou Y., Huang L., Xu X., Yuan C. (2023). Multimorbidity burden and developmental trajectory in relation to later-life dementia: A prospective study. Alzheimer’s Dement..

[448] Leoutsakos J.M.S., Han D., Mielke M.M., Forrester S.N., Tschanz J.T., Corcoran C.D., Green R.C., Norton M.C., Welsh-Bohmer K.A., Lyketsos C.G. (2012). Effect of general medical health on Alzheimer’ progression: The cache County Dementia Progression Study. Int. Psychogeriatr..

[449] Göbel A., Göttlich M., Reinwald J., Rogge B., Uter J.C., Heldmann M., Sartorius A., Brabant G., Münte T.F. (2020). The influence of thyroid hormones on brain structure and function in humans. Exp. Clin. Endocrinol. Diabetes.

[450] Aydin N., Ramazanoglu L., Onen M.R., Yilmaz I., Aydin M.D., Altinkaynak K., Calik M., Kanat A. (2017). Rationalization of the irrational neuropathologic basis of hypothyroidism-olfaction disorders paradox: Experimental study. World Neurosurg..

[451] Chaudhuri J., Mukherjee A., Chakravarty A. (2023). Hashimoto’s encephalopathy: Case series and literature review. Curr. Neurol. Neurosci. Rep..

[452] Michiels C. (2004). Physiological and pathological responses to hypoxia. Am. J. Pathol..

[453] Pruzinyh J.J., Nelson P.T., Abner E.L., Arvanitakis Z. (2018). Relationship of type 2 diabetes to human brain pathology [review]. Neuropathol. Appl. Neurobiol..

[454] McCrimmon R.J. (2021). Consequences of recurrent hypoglycaemia on brain function in diabetes. Diabetologia.

[455] He C., Li Q., Cui Y., Gao P., Shu W., Zhou Q., Wang L., Li L., Lu Z., Zhao Y. (2022). Recurrent moderate hypoglycemia accelerates the progression of Alzheimer’s disease through impairment of the TRPC6/GLUT3 pathway. JCI Insight.

[456] Auer R.N. (2018). Hypoglycemic brain damage. Acute Neuronal Injury.

[457] Czempik P.F., Pluta M.P., Krzych Ł.J. (2020). Sepsis-associated brain dysfunction: A review of current literature. Int. J. Environ. Res. Public Health.

[458] Zhao L.Y., Zhou X.L. (2022). Association of chronic obstructive pulmonary disease with mild cognitive impairment and dementia risk: A systematic review and meta-analysis. World J. Clin. Cases.

[459] Morris G.P., Clark I.A., Vissel B. (2018). Questions concerning the role of amyloid-b in the definition, aetiology and diagnosis of Alzheimer’s disease. Acta Neuropathol..

[460] Wu Z., Wang Z.H., Liu X., Zhang Z., Gu X., Yu S.P., Keene C.D., Cheng L., Ye K. (2020). Traumatic brain injury triggers APP and Tau cleavage by delta-secretase, mediating Alzheimer’s disease pathology. Prog. Neurobiol..

[461] Pluta R., Ułamek-Kozioł M., Januszewski S., Czuczwar S.J. (2020). Participation of amyloid and tau protein in neuronal death and neurodegeneration after brain ischemia. Int. J. Mol. Sci..

[462] Sengupta U., Kayed R. (2022). Amyloid β, Tau, and α-Synuclein aggregates in the pathogenesis, prognosis, and therapeutics for neurodegenerative diseases. Prog. Neurobiol..

[463] Attems J., Jellinger K. (2013). Neuropathological correlates of cerebral multimorbidity. Curr. Alzheimer Res..

[464] Stirland L.E., Russ T.C., Ritchie C.W., Muniz-Terrera G., EPAD Consortium (2019). Associations between multimorbidity and cerebrospinal fluid amyloid: A cross-sectional analysis of the European Prevention of Alzheimer’s Dementia (EPAD) V500.0 Cohort. J. Alzheimer’s Dis..

[465] Mendes A., Du Montcel S.T., Levy M., Bertrand A., Habert M.-O., Bertin H., Dubois B., Epelbaum S., INSIGHT-PreAD study group (2018). Multimorbidity is associated with preclinical Alzheimer’s disease neuroimaging biomarkers. Dement. Geriatr. Cogn. Disord..

[466] Canevelli M., Arisi I., Bacigalupo I., Arighi A., Galimberti D., Vanacore N., D’onofrio M., Cesari M., Bruno G. (2021). Biomarkers and phenotypic expression in Alzheimer’s disease: Exploring the contribution of frailty in the Alzheimer’s disease neuroimaging initiative. Geroscience.

[467] Kim T., Yi D., Byun M.S., Ahn H., Jung J.H., Kong N., KBASE Research Group (2022). Synergistic interaction of high blood pressure and cerebral beta-amyloid on tau pathology. Alzheimer’s Res. Ther..

[468] Zhang J., Chi H., Wang T., Zhang S., Shen T., Leng B., Sun H., Li Z., Li F. (2021). Altered amyloid-β and tau proteins in neural-derived plasma exosomes of type 2 diabetes patients with orthostatic hypotension. J. Alzheimer’s Dis..

[469] Chakrabarty R., Yousuf S., Singh M.P. (2022). Contributive role of hyperglycemia and hypoglycemia towards the development of Alzheimer’s disease. Mol. Neurobiol..

[470] Jakubauskienė E., Vilys L., Pečiulienė I., Kanopka A. (2021). The role of hypoxia on Alzheimer’s disease-related APP and Tau mRNA formation. Gene.

[471] Giridharan V.V., Catumbela C.S., Catalão C.H.R., Lee J., Ganesh B.P., Petronilho F. (2023). Sepsis exacerbates Alzheimer’s disease pathophysiology, modulates the gut microbiome, increases neuroinflammation and amyloid burden. Mol. Psychiatry.

[472] Webers A., Heneka M.T., Gleeson P.A. (2020). The role of innate immune responses and neuroinflammation in amyloid accumulation and progression of Alzheimer’s disease. Immunol. Cell Biol..

[473] Li X., Sundquist J., Zöller B., Sundquist K. (2018). Dementia and Alzheimer’s disease risks in patients with autoimmune disorders. Geriatr. Gerontol. Int..

[474] de Jong F.J., Masaki K., Chen H., Remaley A.T., Breteler M.M., Petrovitch H., White L.R., Launer L.J. (2009). Thyroid function, the risk of dementia and neuropathologic changes: The Honolulu-Asia aging study. Neurobiol. Aging.

[475] Matsushita K., Yamada-Furukawa M., Kurosawa M., Shikama Y. (2020). Periodontal disease and periodontal disease-related bacteria involved in the pathogenesis of Alzheimer’s disease. J. Inflamm. Res..

[476] Wang C., Holtzman D.M. (2020). Bidirectional relationship between sleep and Alzheimer’s disease: Role of amyloid, tau, and other factors. Neuropsychopharmacology.

[477] Bhuniya S., Goyal M., Chowdhury N., Mishra P. (2022). Intermittent hypoxia and sleep disruption in obstructive sleep apnea increase serum tau and amyloid-beta levels. J. Sleep Res..

[478] Katsumoto A., Takeuchi H., Tanaka F. (2019). Tau pathology in chronic traumatic encephalopathy and Alzheimer’s disease: Similarities and differences. Front. Neurol..

[479] Roberts G.W., Gentleman S.M., Lynch A., Graham D.I. (1991). bA4 amyloid protein deposition in brain after head trauma. Lancet.

[480] Romero J.R., Demissie S., Beiser A., Himali J.J., DeCarli C., Levy D., Seshadri S. (2020). Relation of plasma β-amyloid, clusterin, and tau with cerebral microbleeds: Framingham Heart Study. Ann. Clin. Transl. Neurol..

[481] Durazzo T.C., Mattsson N., Weiner M.W. (2016). Alzheimer’s disease neuroimaging initiative: Interaction of cigarette smoking history with APOE genotype and age on amyloid level, glucose metabolism, and neurocognition in cognitive normal elders. Nicotine Tob. Res..

[482] Sánchez-Tapia M., Mimenza-Alvarado A., Granados-Domínguez L., Flores-López A., López-Barradas A., Ortiz V., Pérez-Cruz C., Sánchez-Vidal H., Hernández-Acosta J., Ávila-Funes J.A. (2023). The Gut Microbiota–Brain Axis during Aging, Mild Cognitive Impairment and Dementia: Role of Tau Protein, β-Amyloid and LPS in Serum and Curli Protein in Stool. Nutrients.

[483] Huang L., Zhang Y., Wang Y., Lan Y. (2021). Relationship between chronic noise exposure, cognitive impairment, and degenerative dementia: Update on the experimental and epidemiological evidence and prospects for further research. J. Alzheimer’s Dis..

[484] 496 Alemany S., Crous-Bou M., Vilor-Tejedor N., Mila-Aloma M., Suárez-Calvet M., Salvadó G., Cirach M., Arenaza-Urquijo E.M., Sanchez-Benavides G., Grau-Rivera O. (2021). Associations between air pollution and biomarkers of Alzheimer’s disease in cognitively unimpaired individuals. Environ. Int..

[485] Regev A., Teichmann S.A., Lander E.S., Amit I., Benoist C., Birney E., Bodenmiller B., Campbell P., Carninci P., Clatworthy M. (2017). The human cell atlas. eLIfe.

[486] Mattson M.P. (2015). Late-onset dementia: A mosaic of prototypical pathologies modifiable by diet and lifestyle. npj Aging Mech. Dis..

[487] Hansen J., Meretzky D., Woldesenbet S., Stolovitzky G., Iyengar R. (2017). A flexible ontology for inference of emergent whole cell function from relationships between subcellular processes. Sci. Rep..

[488] Abyadeh M., Gupta V., Paulo J.A., Mahmoudabad A.G., Shadfar S., Mirshahvaladi S., Gupta V., Nguyen C.T., Finkelstein D.I., You Y. (2024). Amyloid-beta and tau protein beyond Alzheimer’s disease. Neural Regen. Res..

[489] Chau D.D.L., Ng L.L.H., Zhai Y., Lau K.F. (2023). Amyloid precursor protein and its interacting proteins in neurodevelopment. Biochem. Soc. Trans..

[490] Nishida K., Matsumura K., Tamura M., Nakamichi T., Shimamori K., Kuragano M., Kabir A.M.R., Kakugo A., Kotani S., Nishishita N. (2023). Effects of three microtubule-associated proteins (MAP2, MAP4, and Tau) on microtubules’ physical properties and neurite morphology. Sci. Rep..

[491] Bok E., Leem E., Lee B.R., Lee J.M., Yoo C.J., Lee E.M., Kim J. (2021). Role of the lipid membrane and membrane proteins in tau pathology. Front. Cell Dev. Biol..

[492] Schütze S., Drevets D.A., Tauber S.C., Nau R. (2023). Septic encephalopathy in the elderly–biomarkers of potential clinical utility. Front. Cell. Neurosci..

[493] Feng L., Gao L. (2024). The role of neurovascular coupling dysfunction in cognitive decline of diabetes patients. Front. Neurosci..

[494] AlAnazi F.H., Al-Kuraishy H.M., Alexiou A., Papadakis M., Ashour M.H.M., Alnaaim S.A., Elhussieny O., Saad H.M., Batiha G.E.-S. (2023). Primary hypothyroidism and Alzheimer’s Disease: A tale of two. Cell. Mol. Neurobiol..

[495] Morrone C.D., Raghuraman R., Hussaini S.A., Yu W.H. (2023). Proteostasis failure exacerbates neuronal circuit dysfunction and sleep impairments in Alzheimer’s disease. Mol. Neurodegener..

[496] Lista S., González-Domínguez R., López-Ortiz S., González-Domínguez Á., Menéndez H., Martín-Hernández J. (2023). Integrative metabolomics science in Alzheimer’s disease: Relevance and future perspectives. Ageing Res. Rev..

[497] Batra R., Arnold M., Wörheide M.A., Allen M., Wang X., Blach C., Levey A.I., Seyfried N.T., Ertekin-Taner N., Bennett D.A. (2023). The landscape of metabolic brain alterations in Alzheimer’s disease. Alzheimer’s Dement..

[498] Cheng X., Wei Y., Qian Z., Han L. (2023). Autophagy balances neuroinflammation in Alzheimer’s disease. Cell. Mol. Neurobiol..

[499] Pereira C.F., Santos A.E., Moreira P.I., Pereira A.C., Sousa F.J., Cardoso S.M., Cruz M.T. (2019). Is Alzheimer’s disease an inflammasomopathy?. Ageing Res. Rev..

[500] Yoon J.H., Hwang J., Son S.U., Choi J., You S.W., Park H., Cha S.Y., Maeng S. (2023). How can insulin resistance cause Alzheimer’s disease?. Int. J. Mol. Sci..

[501] Saroja S.R., Sharma A., Hof P.R., Pereira A.C. (2022). Differential expression of tau species and the association with cognitive decline and synaptic loss in Alzheimer’s disease. Alzheimer’s Dement..

[502] Torres A.K., Jara C., Park-Kang H.S., Polanco C.M., Tapia D., Alarcón F., de la Peña A., Llanquinao J., Vargas-Mardones G., Indo J.A. (2021). Synaptic mitochondria: An early target of amyloid-β and tau in Alzheimer’s disease. J. Alzheimer’s Dis..

[503] Abdul-Rahman T., Ghosh S., Kalmanovich J.B., Awuah A.W., Zivcevska M., Khalifa S., Bassey E.E., Ali N.A., Ferreira M.M.d.S., Umar T.P. (2024). The role of membrane trafficking and retromer complex in Parkinson’s and Alzheimer’s disease. J. Neurosci. Res..

[504] Ekundayo B.E., Obafemi T.O., Adewale O.B., Obafemi B.A., Oyinloye B.E., Ekundayo S.K. (2024). Oxidative Stress, Endoplasmic Reticulum Stress and Apoptosis in the Pathology of Alzheimer’s Disease. Cell Biochem. Biophys..

[505] Rahman S.O., Khan T., Iqubal A., Agarwal S., Akhtar M., Parvez S., Shah Z.A., Najmi A.K. (2023). Association between insulin and Nrf2 signalling pathway in Alzheimer’s disease: A molecular landscape. Life Sci..

[506] Maiese K. (2023). The metabolic basis for nervous system dysfunction in Alzheimer’s disease, Parkinson’s disease, and Huntington’s disease. Curr. Neurovasc. Res..

[507] Li Q.Y., Li X.M., Hu H.Y., Ma Y.H., Ou Y.N., Wang A.Y., Tan L., Yu J.-T. (2023). Associations of Lung Function Decline with Risks of Cognitive Impairment and Dementia: A Meta-Analysis and Systematic Review. J. Alzheimer’s Dis..

[508] Wang M., Wang Y., Wang Z., Ren Q. (2023). The Abnormal Alternations of Brain Imaging in Patients with Chronic Obstructive Pulmonary Disease: A Systematic Review. J. Alzheimer’s Dis. Rep..

[509] Pluta R., Ouyang L., Januszewski S., Li Y., Czuczwar S.J. (2021). Participation of amyloid and tau protein in post-ischemic neurodegeneration of the hippocampus of a nature identical to Alzheimer’s disease. Int. J. Mol. Sci..

[510] Salminen A., Kaarniranta K., Kauppinen A. (2020). ER stress activates immunosuppressive network: Implications for aging and Alzheimer’s disease. J. Mol. Med..

[511] Josephs K.A., Tosakulwong N., Weigand S.D., Murray M.E., Whitwell J.L., Parisi J.E., Dickson D.W., Petersen R.C. (2017). Brain tau deposition linked to systemic causes of death in normal elderly. Neurobiol. Aging.

[512] Wang Y.Y., Sun Y.P., Luo Y.M., Peng D.H., Li X., Yang B.Y., Wang Q.-H., Kuang H.-X. (2021). Biomarkers for the clinical diagnosis of Alzheimer’s disease: Metabolomics analysis of brain tissue and blood. Front. Pharmacol..

[513] Ganeshpurkar A., Swetha R., Kumar D., Gangaram G.P., Singh R., Gutti G., Jana S., Kumar D., Kumar A., Singh S.K. (2019). Protein-protein interactions and aggregation inhibitors in Alzheimer’s disease. Curr. Top. Med. Chem..

[514] Badhwar A., McFall G.P., Sapkota S., Black S.E., Chertkow H., Duchesne S., Masellis M., Li L., Dixon R.A., Bellec P. (2020). A multiomics approach to heterogeneity in Alzheimer’s disease: Focused review and roadmap. Brain.

[515] Pumo A., Legeay S. (2024). The dichotomous activities of microglia: A potential driver for phenotypic heterogeneity in Alzheimer’s disease. Brain Res..

[516] Presa J.L., Saravia F., Bagi Z., Filosa J.A. (2020). Vasculo-neuronal coupling and neurovascular coupling at the neurovascular unit: Impact of hypertension. Front. Physiol..

[517] Filley C. (2012). Cobalamin deficiency. The Behavioral Neurology of White Matter.

[518] Gallart-Palau X., Serra A., Hase Y., Tan C.F., Chen C.P., Kalaria R.N., Sze S.K. (2019). Brain-derived and circulating vesicle profiles indicate neurovascular unit dysfunction in early Alzheimer’s disease. Brain Pathol..

[519] Canavan M., O’Donnell M.J. (2022). Hypertension and cognitive impairment: A review of mechanisms and key concepts. Front. Neurol..

[520] Li D., Liu C. (2022). Conformational strains of pathogenic amyloid proteins in neurodegenerative diseases. Nat. Rev. Neurosci..

[521] Scheres S.H.W., Zhang W., Falcon B., Goedert M. (2020). Cryo-EM structures of tau filaments. Curr. Opin. Struct. Biol..

[522] Yang Y., Arseni D., Zhang W., Huang M., Lövestam S., Schweighauser M., Kotecha A., Murzin A.G., Peak-Chew S.Y., Macdonald J. (2022). Cryo-EM structures of amyloid-β 42 filaments from human brains. Science.

[523] Eisenmenger L.B., Peret A., Famakin B.M., Spahic A., Roberts G.S., Bockholt J.H., Johnson K.M., Paulsen J.S. (2023). Vascular contributions to Alzheimer’s disease. Transl. Res..

[524] Shi Y., Murzin A.G., Falcon B., Epstein A., Machin J., Tempest P., Newell K.L., Vidal R., Garringer H.J., Sahara N. (2021). Cryo-EM structures of tau filaments from Alzheimer’s disease with PET ligand APN-1607. Acta Neuropathol..

[525] Jiménez J.S. (2023). Macromolecular structures and proteins interacting with the microtubule associated tau protein. Neuroscience.

[526] Vogel J.W., Initiative T.A.D.N., Young A.L., Oxtoby N.P., Smith R., Ossenkoppele R., Strandberg O.T., La Joie R., Aksman L.M., Grothe M.J. (2021). Four distinct trajectories of tau deposition identified in Alzheimer’s disease. Nat. Med..

[527] Kaddurah-Daouk R., Rozen S., Matson W., Han X., Hulette C.M., Burke J.R., Doraiswamy P.M., Welsh-Bohmer K.A. (2011). Metabolomic changes in autopsy-confirmed Alzheimer’s disease. Alzheimer’s Dement..

[528] Johnson D.R., Sherry C.L., York J.M., Freund G.G. (2008). Acute hypoxia, diabetes, and neuroimmune dysregulation: Converging mechanisms in the brain. Neuroscientist.

[529] Piva S., McCreadie V.A., Latronico N. (2015). Neuroinflammation in sepsis: Sepsis associated delirium. Cardiovasc. Hematol. Disord. Drug Targets.

[530] Pooler A.M., Polydoro M., Maury E.A., Nicholls S.B., Reddy S.M., Wegmann S., William C., Saqran L., Cagsal-Getkin O., Pitstick R. (2015). Amyloid accelerates tau propagation and toxicity in a model of early Alzheimer’s disease. Acta Neuropathol. Commun..

[531] Sinha M.S., Ansell-Schultz A., Cavatelli L., Hildesjö C., Larsson M., Lannfelt L., Ingelsson M., Hallbeck M. (2018). Alzheimer’s disease pathology propagation by exosomes containing toxic amyloid-beta oligomers. Acta Neuropathol..

[532] Reiss A.B., Arain H.A., Stecker M.M., Siegart N.M., Kasselman L.J. (2018). Amyloid toxicity in Alzheimer’s disease. Rev. Neurosci..

[533] Johnson E.O., Kamilaris T.C., Calogero A.E., Konstandi M., Chrousos G.P. (2013). Effects of short- and long-duration hypothyroidism of function of the rat hypothalamic-pituitary-adrenal axis. J. Endocrinol. Investig..

[534] He X.S., Ma N., Pan Z.L., Wang Z.-X., Li N., Zhang X.-C., Zhou J.-N., Zhu D.-F., Zhang D.-R. (2011). Functional magnetic resource imaging assessment of altered brain function in hypothyroidism during working memory processing. Eur. J. Endocrinol..

[535] Baril A.A., Martineau-Dussault M.È., Sanchez E., André C., Thompson C., Legault J., Gosselin N. (2021). Obstructive sleep apnea and the brain: A focus on gray and white matter structure. Curr. Neurol. Neurosci. Rep..

[536] Ng A.S.L., Wang J., Ng K.K., Chong J.S.X., Qian X., Lim J.K.W., Tan Y.J., Yong A.C.W., Chander R.J., Hameed S. (2021). Distinct network topology in Alzheimer’s disease and behavioral variant frontotemporal dementia. Alzheimer’s Res. Ther..

[537] Wang Z., Alzheimer’s Disease Neuroimaging Initiative (2020). Brain entropy mapping in healthy aging and Alzheimer’s disease. Front. Aging Neurosci..

[538] Garnier-Crussard A., Cotton F., Krolak-Salmon P., Chételat G. (2023). White matter hyperintensities in Alzheimer’s sdisease: Beyond vascular contribution. Alzheimer’s Dement..

[539] Gobel A., Göttlich M., Heldmann M., Georges R., Nieberding R., Rogge B., Sartorius A., Brabant G., Münte T.F. (2019). Experimentally induced subclinical hypothyroidism causes decreased functional connectivity of the cuneus: A resting state fMRI study. Psychoneuroendocrinology.

[540] Marr D. (1982). A Computational Investigation into the Human Representation and Processing of Visual Information.

